# Overcoming the blood–brain barrier by Annexin A1-binding peptide to target brain tumours

**DOI:** 10.1038/s41416-020-01066-2

**Published:** 2020-09-14

**Authors:** Motohiro Nonaka, Misa Suzuki-Anekoji, Jun Nakayama, Hideaki Mabashi-Asazuma, Donald L. Jarvis, Jiunn-Chern Yeh, Kazuhiko Yamasaki, Tomoya O. Akama, Chun-Teng Huang, Alexandre Rosa Campos, Masato Nagaoka, Toshio Sasai, Itsuko Kimura-Takagi, Yoichi Suwa, Takashi Yaegashi, Toshiaki K. Shibata, Kazuhiro Sugihara, Chizuko Nishizawa-Harada, Minoru Fukuda, Michiko N. Fukuda

**Affiliations:** 1 Cancer Center, Sanford-Burnham-Prebys Medical Discovery Institute, La Jolla, CA 92037 USA; 2 Laboratory for Drug Discovery, National Institute of Advanced Industrial Science and Technology, Tsukuba, Ibaraki, 305-8568 Japan; 3 Department of Biological Chemistry, Human Health Sciences, Graduate School of Medicine, Kyoto University, Kyoto, 606-8507 Japan; 4 Department of Molecular Pathology, Shinshu University School of Medicine, Matsumoto, 390-8621 Japan; 5 Department of Molecular Biology, University of Wyoming, Laramie, WY 82071 USA; 6 Biomedical Research Institute, National Institute of Advanced Industrial Science and Technology, Tsukuba, Ibaraki, 305-8566 Japan; 7 Department of Pharmacology, Kansai Medical University, Hirakata, Osaka 573-1010 Japan; 8 Yakult Central Institute, Kunitachi, Tokyo, 186-8650 Japan; 9 Department of Gynecology and Obstetrics, Hamamatsu University School of Medicine, Hamamatsu, 431-3192 Japan

**Keywords:** CNS cancer, Cancer therapy

## Abstract

**Background:**

Annexin A1 is expressed specifically on the tumour vasculature surface. Intravenously injected IF7 targets tumour vasculature via annexin A1. We tested the hypothesis that IF7 overcomes the blood–brain barrier and that the intravenously injected IF7C(RR)-SN38 eradicates brain tumours in the mouse.

**Methods:**

(1) A dual-tumour model was generated by inoculating luciferase-expressing melanoma B16 cell line, B16-Luc, into the brain and under the skin of syngeneic C57BL/6 mice. IF7C(RR)-SN38 was injected intravenously daily at 7.0 μmoles/kg and growth of tumours was assessed by chemiluminescence using an IVIS imager. A similar dual-tumour model was generated with the C6-Luc line in immunocompromised SCID mice. (2) IF7C(RR)-SN38 formulated with 10% Solutol HS15 was injected intravenously daily at 2.5 μmoles/kg into two brain tumour mouse models: B16-Luc cells in C57BL/6 mice, and C6-Luc cells in nude mice.

**Results:**

(1) Daily IF7C(RR)-SN38 injection suppressed tumour growth regardless of cell lines or mouse strains. (2) Daily injection of Solutol-formulated IF7C(RR)-SN38 led into complete disappearance of B16-Luc brain tumour in C57BL/6 mice, whereas this did not occur in C6-Luc in nude mice.

**Conclusions:**

IF7C(RR)-SN38 crosses the blood–brain barrier and suppresses growth of brain tumours in mouse models. Solutol HS15-formulated IF7C(RR)-SN38 may have promoted an antitumour immune response.

## Background

Cancers of the central nervous system (CNS) are the most devastating among human malignancies:^1^ after surgical resection of glioblastoma followed by chemo- or radiotherapy, median survival time is ~12 months.^2^ In addition to primary CNS tumours, brain metastasis occurs in patients with carcinoma of the lung, breast, kidney and colon and in melanoma, and these metastases are difficult to eradicate with currently available chemotherapeutics.^3,4^

Although brain tumour cells cultured in vitro respond to several anticancer drugs, brain tumours in vivo do not due to the blood–brain barrier (BBB), which under healthy conditions protects the CNS from pathogens and toxic materials.^5,6^ Numerous investigators have attempted to overcome this hurdle using brain vasculature surface receptor-mediated proteins,^7^ tumour-penetrating peptides^8–10^ or nanoparticles.^11^ On the other hand, tumour vasculature surrounding a brain tumour is chaotic, allowing large molecules and nanoparticles to overcome the BBB by passing through gaps between endothelial cells.^12^ However, drugs targeting the brain often rely on passive diffusion and are therefore used at maximum tolerable dose, causing adverse side effects. Thus, efficient treatment of brain tumours requires both tumour vasculature targeting and penetration by a therapeutic to overcome the BBB.^13^

Previously, we identified several linear 7-mer carbohydrate mimetic peptides by screening a peptide-displaying phage library with an anticarbohydrate antibody^14^ Subsequently, we identified a peptide that we designate IF7 that binds to annexin A1 (Anxa1), a specific endothelial cell-surface marker of malignant tumours.^15^ Upon intravenous injection into tumour-bearing mice, IF7-conjugated anticancer drugs suppressed growth of colon, melanoma, breast, prostate and lung tumours in mouse models.^16,17^ Others tested successfully IF7-conjugated taxol nanoparticles on subcutaneous tumours in mouse.^18^ Our data suggest that, after binding to Anxa1 on tumour vasculature surface, IF7 is transported across endothelial cells from the luminal surface to the basal membrane via transcytosis and released to the tumour stroma; thus, we hypothesised that an IF7-conjugated chemotherapeutics could cross the BBB to deliver drug to brain stroma.

## Methods

### Materials

Unless otherwise noted, peptides used in this study including IF7C(RR) or IFLLWQRCRR and C(RR) or CRR, were synthesised by GenScript (Piscataway, NJ). Human and mouse MC16 (the N-terminal 15 residues of ANXA1 and Anxa1 plus a cysteine residue, MAMVSEFLKQAWFIEC and MAMVSEFLKQARFLEC) and their mutants were synthesised by Bio-Synthesis (Lewisville, TX). Rabbit anti-Annexin 1 antibody was from Invitrogen, and rabbit anti-CD34 antibody was from Abcam. FITC-conjugated anti-CD8α antibody was from BD Pharmingen, and unconjugated anti-CD8α was from Abcam. IF7C(RR)-SN38 and C(RR)-SN38 were synthesised at CalChem Synthesis, San Diego, CA. Irinotecan was from Sigma (St. Louis, MO).

### Cell lines

All cell lines, including rat glioma C6, mouse melanoma B16F10 and mouse Lewis lung carcinoma LL/2, were from American Type Cell culture, and were cultured in Dulbecco’s-Modified Eagles + F2 medium supplemented with 10% foetal bovine serum and 100 units each/mL penicillin and streptomycin. C6-Luc cells, B16F1-Luc cells and LL/2-Luc cells were produced by infection with lentivirus harbouring firefly luciferase. The lentiviral vector PGK-Luc was produced at the Virus Core Facility of the Sanford-Burnham-Prebys Medical Discovery Institute (SBP).

### Preparation of fluorescence-labelled IF7C(RR) and C(RR)

Alexa Fluor 488 C5-maleimide (Molecular probe) was used for the fluorescence labelling of IFLLQARC(RR) and C(RR) peptides. Each peptide (1 mg) was reacted with the molar equivalent of Alexa Fluor 488 C5-maleimide in 500 μl DMSO. After incubation for 4 h, the sample was applied to a Bond Elut SCX column (100 mg, Agilent Technology, Japan) prewashed with water, methanol and dimethylformamide (DMF). After washing with 3 ml of 50% DMF in water, A488-labeled peptide was eluted with 3 ml of 50% DMF in water containing 1 M NaCl. The eluate was dried in vacuo, and the dried sample was suspended in 2 ml chloroform. The solution was then washed four times with 2 ml water, and chloroform layer was dried in vacuo.

### Preparation of multivalent MC16

Wild-type or mutant forms of MC16 peptide (0.95 mg) were reacted with 3.6 mg of sunbright PTE-200MA in 3.5 ml DMSO at room temperature for 5 h. Dimeric MC16 was purified by gel filtration chromatography using Sephacryl S-200HR, lyophilised, and dissolved at 2 mg/ml with TBSCC.

### Fluorescence correlation spectroscopy (FCS)

A mixture of IF7C-A546 with Anxa1 or MC16 (50 μl) was loaded into each well of a 96-well plate and incubated at 37 °C for 1 h. FCS was performed with the MF20 analytical system (Olympus) using a 543 nm He–Ne laser for illumination. Each sample was measured five times with a data acquisition time of 10 sec per measurement. IF7C(RR) binding was defined as an increase in translational diffusion time relative to a control.

### Kinetics of IF7CRR binding to Anxa1 or MC16 tetramer

Kinetic analysis was performed with a 27-MHz QCM (Affinix QN μ, Initium Inc).^19^ The synthetic peptide IF7C(RR) was immobilised through maleimide-thiol coupling to a self-assembled monolayer (SAM) formed on the gold electrode of the sensor cell as follows. SAM was incubated with 100 μl of 40% ethanol containing 1 mM PEG_6_NH_2_ undecanethiol and 9 mM PEG_3_OH undecanethiol at room temperature for 1 h. After washing the cell with MilliQ water, 100 μl of 9 mM HEPES buffer, pH 7.6, containing 2 mM sulfo-GMBS was applied onto the cell to introduce a maleimide group to the SAM NH_2_-moiety. Tris-HCl, pH 7.6 (50 mM, 450 μl) was added to the cell, and then IF7C(RR) was immobilised to the SAM by injecting peptide solution. Unreacted maleimides were blocked with 50 mM Tris-HCl buffer, pH 7.6, containing 6.5 mM 2-aminoethanethiol. The sensor cell was equilibrated with 466 μl saline buffer (150 mM NaCl, 5 mM CaCl_2_, 0.05% CHAPS, 5 mM 2-aminoethanethiol, 50 mM Tris-HCl, pH 7.6). Various concentrations of Anxa1 or MC16 tetramer were injected into the sensor cell and reduction in QCM signals at 25 °C was recorded over time. ∆F was calculated as: ∆F = B_max_ (final conc.)/(*K*_d_ + final conc), where the final concentration of each (Anxa1 or MC16 tetramers) is nM. Kinetic analysis was performed by AQUA ver 2.0 (Initium Inc.).

### Expression and purification of recombinant ANXA1

Recombinant, full-length ANXA1 and corresponding deletion mutants (Δ4, Δ6–9, Δ12, Δ13, Δ15 and Δ28) were expressed using the baculovirus insect cell expression system,^20^ with following modifications. Briefly, recombinant baculoviruses were prepared by recombining BacPAK6Δchi/cath baculovirus DNA with pAcP(−)-based baculovirus transfer vectors, which encode the transgene under control of the baculovirus *p6.9* promoter. All recombinant ANXA1s were expressed with N-terminal honeybee melittin signal peptide followed by a His_8_-tag and the enterokinase recognition sequence, DDDDR. Proteins were purified from Sf9 culture supernatants harvested 42 h after infection using HisPur Ni-NTA resin (Pierce). Untagged ANXA1 and deletion mutants were isolated by His-tagged enterokinase (Genscript) treatment followed by Ni-affinity chromatography.

### Use of vertebrate animals

Mouse protocols adhered to the NIH Guide for the Care and Use of Laboratory Animals and were approved by the Institutional Review Committees at SBP, at Advanced Industrial Science and Technology (AIST), and Kyoto University. One in vivo biotinylation experiment was conducted at ExploreBiolabs (San Diego, CA).

### Generation of brain tumour models in the mouse

C6-Luc cells (5 × 10^4^ suspended in 2 μl PBS) were injected into C57BL/6 mouse brain striatum using a stereotaxic frame as described previously.^21^ Seven days later, mice underwent imaging for luciferase-expressing tumours. To do so, 100 μl luciferin (30 mg/ml PBS) was injected peritoneally. Mice were then anesthetised under isoflurane gas (20 ml/min) supplemented with oxygen (1 ml/min) and placed under a camera equipped with a Xenogen IVIS 200 imager at both the SBP and AIST animal facilities. Photon numbers were measured for 1–10 sec or for 1 min. For the dual-tumour model, C6-Luc cells were similarly injected into the brain of NOD-SCID mice. When a brain tumour became detectable by IVIS imager, C6-Luc cells (2 × 10^5^ suspended in 100 μl PBS) were injected subcutaneously into the dorsal flank of the same mice. Photon numbers in brain and subcutaneous tumours were measured using the IVIS imager. The dual-tumour model was also produced using B16-Luc cells in the isogeneic C57BL/6 mouse. B16-Luc cells (5 × 10^4^) were injected into brains of 8–10-weeks-old C57BL/6 female mice as described above for C6-Luc cells. When brain tumours became detectable, B16-Luc cells (2 × 10^5^ cells) were injected subcutaneously into the dorsal flank of the same mouse.

### Monoclonal anti-MC16 antibody

Anti-MC16 monoclonal antibody (clone 31–8D, mouse IgG2b) was prepared by ITM Co. Ltd (Matsumoto, Japan) by immunising Balb/c mice with human MC16 peptide conjugated to BSA and emulsified in Freund’s incomplete adjuvant. Lymphocytes harvested from splenic tissue were fused with mouse myeloma SP2/0-Ag14 cells by suspending in media containing 50% polyethylene glycol. Resultant hybridoma cells were cloned by a limited dilution. Culture supernatants were assessed for anti-MC16 antibody by ELISA for MC16 peptide binding activity.

### In vivo biotinylation

The method described by Rajotte^22^ was followed. Briefly, a C57/BL6 mouse was anesthetised with avertin. One hundred μl of NHS-LC-biotin (Pierce, Rockford, IL; 1 mg/ml) in PBS was injected intravenously through the tail vein. Fifteen minutes later, the animal was perfused with 15 ml PBS through the heart. Brain tumour and representative organs were isolated, and tissue lysates prepared by homogenisation following solubilisation with PBS containing 1% NP-40 and protease inhibitors (Roche, Indianapolis, IN). In vivo biotinylated tissue lysates were subjected to immunoprecipitation using monoclonal anti-MC16 antibody, anti-CD34 antibody (a positive control for membrane proteins), anti-α-tubulin antibody (a negative control for cytoplasmic proteins) and mouse IgG2b (an isotype-matched mouse IgG control for MC16 antibody) plus protein A-conjugated agarose beads. Biotinylated immunoprecipitates were quantitated using peroxidase avidin and the peroxidase colour reaction. Biotinylated immunoprecipitates with anti-MC16 antibody were eluted from protein A beads with 0.2 M glycine-HCl buffer, pH 2.4, and neutralised with 2 M Tris-HCL buffer, pH 8.0. Samples were analysed by a peroxidase-avidin blot or by proteomics after recapturing with avidin-magnetic beads.

### Proteomics mass spectrometry (MS)

Following immunoprecipitation, proteins were digested overnight directly on-beads using mass spec grade Trypsin/Lys-C mix (Promega, Madison, WI) following cysteine reduction with 10 mM tris(2-carboxyethyl) phosphine (TCEP) and alkylation with 30 mM iodoacetamide (IAA). The digested samples were desalted using a C18 TopTip (PolyLC, Columbia, MD). Peptide samples were then analysed by LC-MS/MS using a Proxeon EASY nanoLC system (Thermo Fisher Scientific) coupled to an Orbitrap Elite mass spectrometer (Thermo Fisher Scientific). Peptides were separated using an analytical C_18_ Acclaim PepMap column 0.075 × 500 mm, 2 µm particles (Thermo Scientific) in a 90-min gradient of 2–28% solvent B at a flow rate of 300 nL/min. The mass spectrometer was operated in positive data-dependent acquisition mode. MS1 spectra were measured with a resolution of 60,000, an AGC target of 1e6 and a mass range from 350 to 1400 *m/z*. Up to 10 MS2 spectra per duty cycle were triggered, fragmented by CID and acquired in the ion trap with an AGC target of 1e4, an isolation window of 2.0 *m/z* and a normalised collision energy of 35. Dynamic exclusion was enabled with duration of 30 sec.

### MS data analysis

All mass spectra were analysed with MaxQuant software version 1.5.5.1. MS/MS spectra were searched against the *Homo sapiens* Uniprot protein sequence database (version July 2016) and GPM cRAP sequences (commonly known protein contaminants). Precursor mass tolerance was set to 20 ppm and 4.5 ppm for the first search where initial mass recalibration was completed and for the main search, respectively. Product ions were searched with a mass tolerance 0.5 Da. The maximum precursor ion charge state used for searching was 7. Carbamidomethylation of cysteines was searched as a fixed modification, while oxidation of methionines and acetylation of protein N-terminal were searched as variable modifications. Enzyme was set to trypsin in a specific mode and a maximum of two missed cleavages was allowed for searching. The target-decoy-based false discovery rate (FDR) filter for spectrum and protein identification was set to 1%.

### Fluorescence microscopy

Brain tissues from mice intravenously injected with fluorescent Alexa 488-labeled IF7 peptide or control peptide were cryo-sectioned and immunostained with anti-CD31 antibody and mounted with medium containing Hoechst 33342. Tissue sections were observed under a Keyence BZ-X 710 microscope (Osaka, Japan).

### Histology and immunohistochemistry

Tissue microarray slides (US Biomax GL806e, GL807a, PR807c, BC000120, BC081120c and BC041115d) of tumours from human patients, including those harbouring brain tumours, were stained using anti-Anxa1 antibodies (rabbit IgG) and mouse monoclonal anti-MC16 antibody. Before immunostaining, antigen retrieval was carried out by microwaving deparaffinised tissue sections in 10 mM Tris-HCl buffer, pH 8.0, containing 1 mM EDTA. Peroxidase-conjugated anti-rabbit IgG or anti-mouse IgG were used as secondary antibody. Peroxidase activity was visualised with diaminobenzidine-H_2_O_2_ and slides were counter-stained with Haematoxylin. For an isotype control antibody, mouse IgG2b (Medical & Biological Laboratories, Nagoya, Japan) was used in place of anti-MC16.

### Treatment of tumour-bearing mice with IF7C(RR)-SN38

IF7C(RR)-SN38 was injected intravenously through the tail vein into tumour-bearing mice once photon number of C6-Luc brain tumour reached 5 × 10^4^, which usually occurred after 2 weeks. IF7C(RR)-SN38 (142 μg or 0.0567 μmoles) dissolved in 10 μl dimethylsulfoxide (DMSO) diluted with 100 μl of 50% Cremophore EL in ethanol plus 90 μl PBS was injected intravenously through the tail vein. As the average body weight of a mouse was 18 g, IF7C(RR)-SN38 dose per injection was 3.15 μmoles/kg. For B16-Luc brain tumours, IF7C(RR)-SN38 formulated with Cremophore EL was used at the dosage 7.0 μmoles/kg. Irinotecan (CPT-11), a water soluble SN38 prodrug, was dissolved in PBS and intravenously injected into mice at 50 μmoles /kg. In another set of experiments, brain tumour-bearing mice were treated with IF7C(RR)-SN38 (100 μg or 0.0412 μmoles) dissolved in 10 μl DMSO followed by dilution with 100 μl 10% Solutol HS15 in water. In that case, drug dosage was 2.5 μmoles/kg. Similarly formulated C(RR)-SN38 served as control.

### Statistical analysis

Statistical analyses were performed using GraphPad Prism program. Datasets were compared using Student’s unpaired *t*-test (two-tailed). A *p* value ≤ 0.05 was considered significant.

## Results

### Presence of Anxa1 N-terminal domain in the tumour vasculature surface

**Fig. 1:**
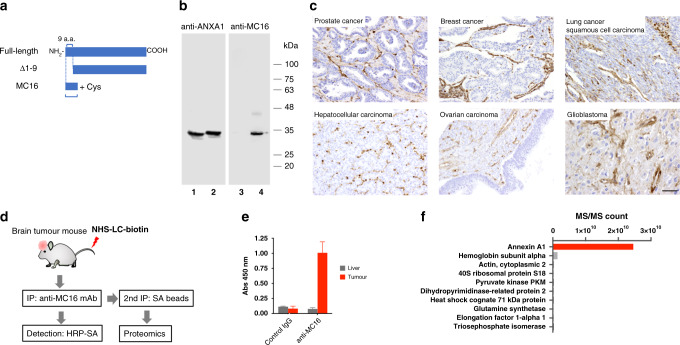
Presence of the Anxa1 N-terminal domain on the vasculature surface of malignant tumours. **a** Schematic of full-length ANXA1, N-terminal deletion mutant Δ9, and N-terminal domain peptide MC16. **b** Western blot of recombinant human ANXA1 proteins either lacking the N-terminal nine amino acid residues Δ9 mutant (lanes 1 and 3) or of full-length protein (lanes 2 and 4). Proteins were reacted with polyclonal rabbit anti-human Anxa1 antibody (left panel) or with monoclonal anti-MC16 antibody (right panel). **c** Immunohistochemistry of human tumour tissue sections using anti-MC16 antibody. Tumour tissues are as indicated. Scale bars, 100 μm. **d** Procedures of in vivo biotinylation of vascular surface proteins following immunoprecipitation by anti-MC antibody and recapturing the biotinylated peptides for proteomics analysis. **e** Levels of in vivo biotinylated vasculature surface proteins immunoprecipitated by anti-MC16 antibody or isotype-matched mouse IgG from a mouse brain tumour model. Rat C6 glioma cells were inoculated into the brain of SCID mice. The brain tumour model mouse was in vivo biotinylated, and brain tumour and liver tissues were subjected to immunoprecipitation. Levels of biotinylated immunoprecipitates were measured by a peroxidase-conjugated avidin and peroxidase colour reaction, as indicated by the OD450. **f** Quantitative proteomics of tryptic fragments of in vivo biotinylated proteins immunoprecipitated by anti-MC16 antibody.

We showed previously that IF7 binds to the N-terminal domain of Anxa1 in vitro.^16^ However, one study suggest that Anxa1 protein on the tumour vasculature surface may lack the N-terminal domain.^23^ Thus, we asked whether the N-terminal domain is present on the tumour vasculature surface. To do so, we raised a mouse monoclonal antibody specific to a linear 16-mer peptide designated as MC16, corresponding to the N-terminal domain of human ANXA1: human MC16 peptide includes 15 amino acids from Met^1^ to Glu,^15^ plus a C-terminal Cys (Fig. 1a). Anti-MC16 antibody bound to recombinant full-length human ANXA1 protein but not to an ANXA1 deletion mutant lacking the N-terminal nine residues (Fig. 1b) (Supplementary Fig. 1). Immunohistochemical analysis of various clinical specimens with this antibody revealed positive signals located at endothelial cells lining malignant tumour tissues in specimens from prostate, breast, lung, liver, ovary and brain cancers (Fig. 1c, Supplementary Figs. 2–4). These observations strongly suggest that the ANXA1 N-terminus is present on endothelial cells in many types of malignant human tumours, although immunostaining alone did not reveal whether the antigen was on the cell surface or in the cytoplasm.

To determine whether the MC16 domain is present on the luminal side of the plasma membrane, we employed in vivo biotinylation (Fig. 1d) of mouse brain tumours by injecting NHS-LC-biotin into tumour-bearing mice, as described previously.^24^ After perfusing mice with PBS, we isolated brain tumours and immunoprecipitated detergent-solubilised membrane proteins using the anti-MC16 antibody and protein A-conjugated beads. Plate binding assays of precipitates indicated high levels of biotinylated materials bound by the anti-MC16 antibody in brain tumour lysates, whereas biotinylated materials in tumour lysates treated with control antibody or those from liver lysates showed significantly lower levels biotinylated material (Fig. 1e). We obtained similar results for subcutaneous tumours in mouse (Supplementary Fig. 4). Analysis of controls, namely the endothelial cell-surface membrane protein CD34 and cytosolic α-tubulin, showed comparable positive CD34 staining and negative α-tubulin staining in both tumour and normal tissues (Supplementary Fig 5). However, when immunoprecipitates from these tumours were analysed by a protein gel following avidin blot, we did not detect biotinylated proteins (data not shown). We speculate that those biotinylated materials were short peptide fragments that were poorly retained on the blot filter. Thus, we analysed biotinylated immunoprecipitates by proteomics after eluting materials from protein A beads with acid and recapturing them on avidin-magnetic beads (Fig. 1d). That analysis revealed that those fragments were predominantly Anxa1 fragments, (Fig. 1f, Supplementary Table 1) and identified 30 Anxa1-derived fragments, including the full-length peptide (346 aa) (Table 2). These results collectively indicate that the Anxa1 N-terminal domain is cleaved from the rest of Anxa1 protein and displayed on the vasculature surface in our mouse brain tumour model. This conclusion is consistent with the model that this domain interacts with the cell membrane, as revealed by ANXA1 X-ray crystallography.^25^

### Binding of IF7 peptide to ANXA1 and MC16 in vitro

**Fig. 2:**
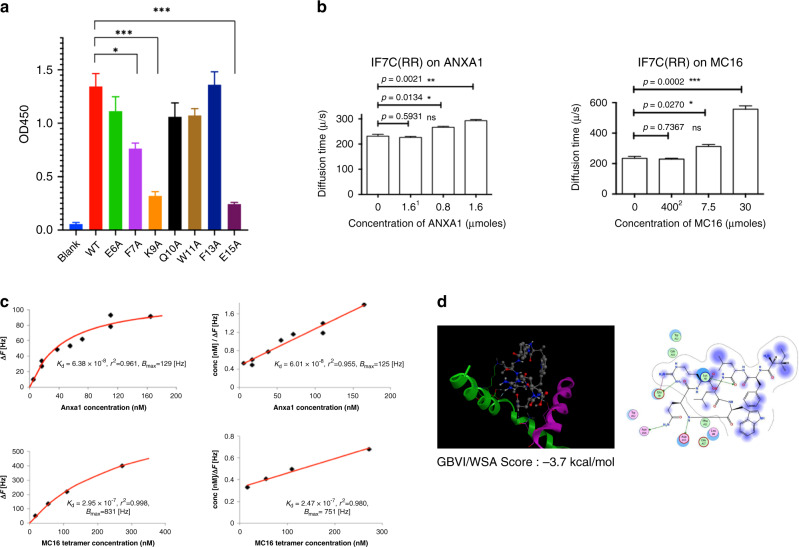
Binding of IF7 to the ANXA1 N-terminal domain and full-length ANXA1 protein in vitro. **a** Plate binding assay of N-terminal biotinylated IF7C(RR) to human MC16 peptide and its mutants. Each synthetic MC16 and its mutant peptide (10 nmoles) was bound to maleimide-activated plastic well through its C-terminal cysteine residue. Biotinylated IF7C(RR) peptide (1 μg/mL) was added to each well and binding of biotinylated peptide was detected by peroxidase-conjugated streptavidin and peroxidase colour reaction. **b** FCS analysis of IF7 with ANXA1 (left) and with MC16 peptides (right) in solution. Note that the diffusion time of IF7C(RR) with N-terminally His-tagged ANXA1 did not significantly increase whereas that with untagged ANXA1 increased significantly compared to the control, suggesting IF7 binding to untagged ANXA1 protein in solution. Note that the diffusion time of IF7 with MC16 monomer did not increase whereas that with tetrameric MC16 increased significantly compared to control, suggesting IF7 binding to multimeric MC16 in solution. **c** Kinetics of IF7 binding to Anxa1 or MC16 by QCM, which determines mass per unit area by measuring change in frequency of an IF7-coated sensor. **d** Computer simulated structural model for IF7 binding to the N-terminal domain of ANXA1. Proposed model was deduced by our previous study suggested that IF7 binds to an Anxa1 dimer^16^ and results shown here suggesting that MC16 polymerisation is required for IF7 binding. The ANXA1 dimer structure was constructed by the Zdock module for protein–protein docking^26^ and the 1HM6 X-ray structure of full-length ANXA1 added to the 1MCX core domain at residue 40.^25, 57^ The modelled structure was then hydrogenated using the Protonate 3D module in MOE. After partial charges were assigned using the AMBER99 force field,^58^ hydrogen atoms were minimised. The dimer structure proposed here was ranked 37th in the top 2000 structures by this program. To identify a potential IF7 binding pocket within the dimer, the Alpha Site Finder module in MOE was used. The proposed model was further validated by dG scoring, which was calculated using MOE software with GBVI/WSA, a program allows us to compare calculated and observed energetics.^59^ The IF7 docking pose was calculated to be −3.7 kcal/mol.

We next assessed IF7 binding to recombinant human ANXA1 protein and human MC16 peptide in three ways (Fig. 2a-c). First, we added biotinylated IF7 in solution to plastic wells coated with monomeric MC16 and determined IF7 binding by peroxidase-conjugated streptavidin and a peroxidase colour reaction (Fig. 2a). IF7 bound to WT MC16; however, when we used MC16 mutant peptides F7A, K9A and E15A, binding was significantly reduced, suggesting that F7, K9 and E15 residues function in IF7 binding. Second, we assessed binding of IF7 and MC16 in solution by fluorescence correlation spectrometry (FCS) (Fig. 2b) and found that IF7 bound to ANXA1 exhibiting a free N-terminus but not to N-terminally His-tagged ANXA1. This last result was consistent with our previously reported plate binding analysis showing that IF7 bound ANXA1 with a free N-terminus but not N-terminally His-Tagged ANXA1.^16^ FCS analysis also showed that IF7 bound to tetrameric rather than monomeric MC16, suggesting that MC16 polymerisation is required for efficient binding in solution. Finally, we determined binding kinetics of ANXA1 and tetrameric MC16 by quartz-crystal microbalance (QCM) and found that the Kd for ANXA1 was 6.01 × 10^–8^M and was 2.47 × 10^–7^M for tetrameric MC16 (Fig. 2c). Based on these results plus our previous finding that IF7 binds to an ANXA1 dimer (10), we hypothesised that ANXA1 N-termini dimerise through IF7 binding. Thus, we modelled the ANXA1 dimer using the Zdock module for protein–protein docking.^26^ The IF7 docking pose was calculated to be −3.7 kcal/mol (Fig. 2d).

### IF7 peptide targeting and penetration to the brain tumour in vivo in the mouse

**Fig. 3:**
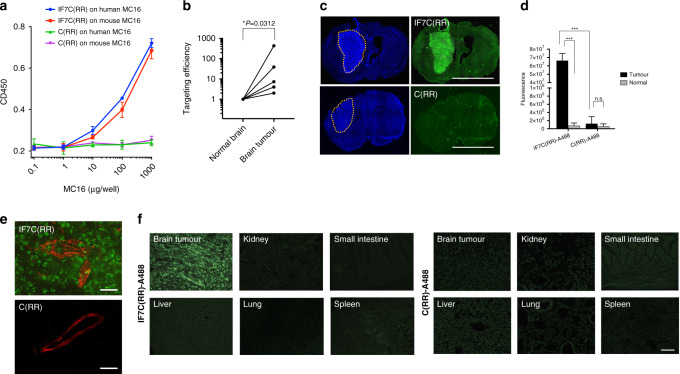
Targeting of IF7 to the brain tumour vasculature in vivo in the mouse. **a** Binding of IF7 and control peptides to mouse and human MC16 peptides by plate assay. Biotinylated IF7C(RR) and control C(RR) was assessed for their binding to MC16 in a similar manner as in Fig. 2a. **b** Targeting of fluorescent-labelled IF7 peptide to the brain tumour vasculature in the mouse. Alexa 488-tagged IF7C(RR) was injected intravenously to brain tumour-bearing mice. Thirty minutes later, the brain tumour tissues and normal tissues in the same mouse were solubilised and fluorescence signals of detergent-solubilised tissues were measured. **c** Alexa 488-tagged IF7C(RR) or control C(RR) peptide was injected through the tail vein of brain tumour-bearing mice harbouring C6-Luc glioma tumours. Blue fluorescence (left panels) represents nuclear Hoechst 33342 and green fluorescence (right) represents Alexa 488. Scale bars: 5 μm. **d** Quantitative image analysis of green fluorescence inside (Tumour) and outside (Normal) brain tumours. Error bars denote means ± SEM. Statistical analysis was assessed by Student’s *t*-test. **e** High magnification images of tumour tissue sections from IF7C(RR)-A488- or C(RR)-A488-injected mice. Sections were immunostained for the endothelial cell-surface marker CD31 using an Alexa 594 (red)-conjugated anti-rat IgG antibody. Scale bars: 40 μm. **f** Fluorescence micrographs of representative organs from brain tumour-bearing mice injected with IF7C(RR)-A488 (left) and C(RR)-A488 (right).

Prior to assess IF7 capacity to target brain tumours in vivo in the mouse, IF7 binding to N-terminal domain of mouse MC16 was confirmed (Fig. 3a). We injected brain tumour-bearing mice intravenously through the tail vein with the IF7C(RR)-A488 construct. Twenty minutes after intravenous injection, brain tumour and surrounding normal brain tissues were isolated. Fluorescence of detergent-solubilised tissues showed higher value in tumours than normal tissues (Fig. 3b). Fluorescence microscopy of sections of mouse brain tissue prepared 20 min later showed bright fluorescence in the tumour (Fig. 3c, upper panel). Tissue sections from a mouse with brain tumour injected with control C(RR)-A488 construct showed no fluorescence signals in the brain tumour (Fig. 3c, lower panel). Quantitative analysis of fluorescence in IF7C(RR)-A488-injected brain tumours showed signals significantly greater than those seen in normal brain tissue, differences not seen when we evaluated control C(RR)-A488-injected tumours (Fig. 3d). Analysis of brain tumour tissue at higher magnification revealed IF7C(RR)-A488 fluorescent signals in cytoplasm and/or nuclei of cancer cells, whereas green fluorescence in brain stroma was not seen when we used control C(RR)-A488 (Fig. 3e). Micrographs of representative organs from the same mouse showed no significant fluorescence signal (Fig. 3f, left). Brain tumours and representative organs from an animal injected with control C(RR)-A488 showed background fluorescence (Fig. 3f, right). These results strongly suggest that intravenously injected IF7 targets the brain tumour vasculature and penetrates the tumour vasculature, thus overcoming the BBB, to reach cancer cells.

### Therapeutic activity of IF7C(RR)-SN38 on brain tumours in the mouse

To assess potential therapeutic activity of IF7-conjugated drugs against brain tumours, we chose SN38, the active component of irinotecan (CPT-11), which is used clinically to treat brain cancer.^27–29^ Treatment of C6 cells and mouse melanoma B16 cells with IF7C(RR)-SN38 or control C(RR)-SN38 in vitro revealed that cytotoxic activities of IF7C(RR)-SN38 were comparable to those of free SN38 (Supplementary Fig. 5).

**Fig. 4:**
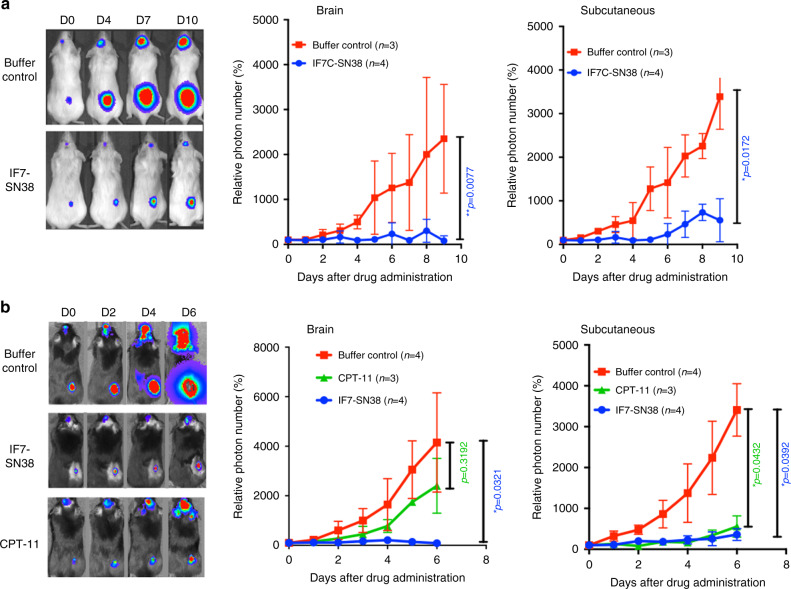
Effect of IF7C(RR)-SN38 on the dual-tumour model mice. **a** Dual-tumour model was established with C6-Luc cells in NOD-SCID mice. C6-Luc cells were grown both in brain and under the skin of mice. When tumours in brain and subcutaneous grew by showing photon number more than 2×10^4, IF7C(RR)-SN38 (3.15 μmoles/kg) was injected intravenously daily through tail vein for 9 days. **b** Effect of IF7C(RR)-SN38 on a dual-tumour model with B16-Luc melanoma cells in C57BL/6 mice. Growth of B16-Luc cells were monitored as in a similar manner as in **a**, followed by intravenous injection with either IF7C(RR)-SN38 (7.0 μmoles/kg), irinotecan (CPT-11) (50 μmoles/kg) or buffer control daily for 6 days. The drug was diluted in Cremophore EL. In each **a** and **b**, error bars denote means ± SEM. Statistical analysis was assessed by Student’s *t*-test. Note that effect of IF7C(RR)-SN38 on brain tumours is comparable to that on subcutaneous tumours regardless of tumour cell line and mouse strain.

Next, to compare drug dosages required to suppress growth of brain versus subcutaneous tumours in vivo, we undertook experiments with a dual-tumour model, in which a single SCID mouse received C6-Luc cells both in brain and under the skin (Fig. 4a, Supplementary Fig. 8A). Mice were then injected intravenously with IF7C(RR)-SN38 and tumour growth in both locations was assessed in vivo by photon number. Treatment with IF7C(RR)-SN38 significantly suppressed tumour growth relative to buffer controls, both in brain and under the skin, at a dosage of 3.15 μmoles/kg.

To confirm these observations, we employed a comparable dual-tumour model using melanoma B16-Luc cells implanted in isogeneic C57BL/6 mice (Fig. 4b, Supplementary Fig. 8B). Intravenously injected IF7C(RR)-SN38 significantly antagonised growth of both brain and subcutaneous tumours compared to buffer controls at a dosage of 7.0 μmoles/kg. When we used the SN38 prodrug irinotecan in the absence of tumour vasculature-targeting peptide at dosages as high as 50 μmoles/kg, irinotecan suppressed subcutaneous tumour growth but minimally suppressed brain tumour growth. Overall, these results suggest that, at least in these mice dual-tumour models shown here, IF7C(RR)-SN38 suppresses brain tumour growth as effectively as subcutaneous tumour growth.

**Fig. 5:**
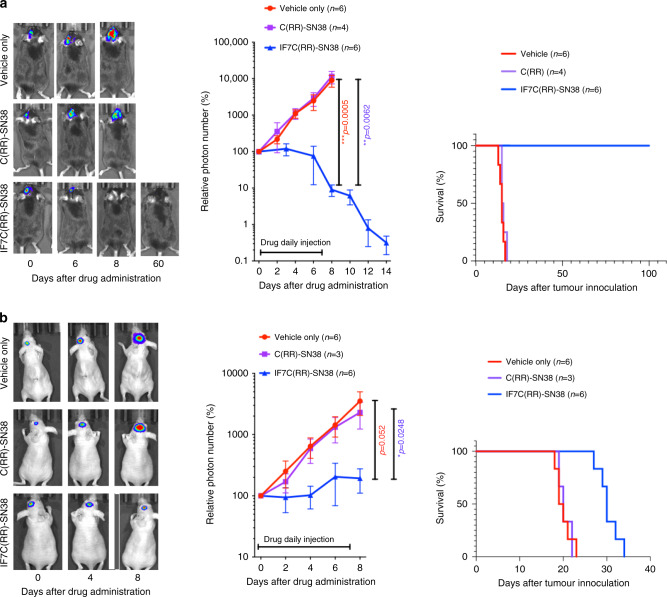
Therapeutic effect of IF7C(RR)-SN38 formulated with Solutol HS15. **a** Effect of IF7C(RR)-SN38 on B16-Luc brain tumours in C57BL/6 mice. **b** Effect of IF7C(RR)-SN38 on C6-Luc brain tumours in nude mice. In these experiments, drug dosage was 2.5 μmoles/kg each for IF7C(RR)-SN38 or C(RR)-SN38 (control), diluted with 10% Solutol HS15 in water and administered daily for 7 days. Note that the B16 brain tumours continued shrinking after ceasing administration of IF7C(RR)-SN38 in C57BL/6 mice. In each **a** and **b**, error bars denote means ± SEM. Statistical analysis was assessed by Student’s *t*-test.

In experiments shown in Fig. 4, we dissolved IF7C(RR)-SN38 in Cremophore EL prior to injection. This non-ionic detergent is used clinically to administer taxol, although some have voiced concerns about potential inflammatory effects.^30,31^ Thus, we conducted experiments in which we dissolved IF7C(RR)-SN38 with Solutol HS15, a non-ionic surfactant with low toxicity. The therapeutic effect of IF7C(RR)-SN38 with 10% Solutol HS15 in water against B16-Luc brain tumours in isogenic C57BL/6 mice was significantly improved (Fig. 5a, Supplementary Fig. 9A) relative to administration in Cremophore EL (Fig. 4b): tumours began shrinking during the first week of daily injections at dosages as low as 2.5 μmoles/kg, continued shrinking during the second week without drug injection, and then completely disappeared. Mice survived after cessation of drug treatment without showing signs of B16-Luc cell growth in brain or other parts of their body for more than 3 months, suggesting complete remission (Fig. 5a, right). Similar treatment of nude mice harbouring of C6-Luc tumours suppressed the tumour growth, however, did not promote complete disappearance of brain tumours (Fig. 5b, Supplementary Fig. 9B). These results suggest an involvement of immune response for complete disappearance of brain tumours in C57BL/6 mice.

### Immune response to brain tumours by IF7C(RR)-SN38-treated mice

**Fig. 6:**
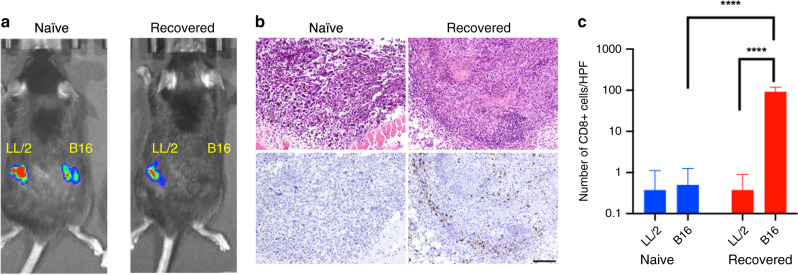
Immunological rejection of the cancer cells from the mouse recovered from brain tumour by IF7C(RR)-SN38 therapy. **a** Growth of two syngeneic cancer lines in naïve and brain tumour-recovered mice 4 days after subcutaneous injection of LL/2-Luc or B16-Luc cells. **b** Immunohistochemistry with anti-CD8 antibody of B16-Luc cells at subcutaneous injection sites, 20 h after B16-Luc cell injection. Scale bars: 100 μm. **c** Quantitative analysis of CD8^+^ cells in subcutaneous injection sites.

The presence of tumour-infiltrating lymphocytes, especially CD8^+^ cytotoxic T cells, is correlated with better prognosis of various cancers.^32,33^ To assess possible involvement of cytotoxic T cells in an antitumour response by host mice, we injected two isogenic cell lines, B16-Luc and LL/2-Luc, into naïve C57BL/6 mice and also into C57BL/6 mice that recovered from brain B16-Luc tumours following IF7C(RR)-SN38 treatment. When these cells were injected subcutaneously in a naïve mouse, both lines formed tumours (Fig. 6a, left). By contrast, when cells were injected into mice that had recovered from brain B16-Luc tumours, LL/2-Luc tumours grew but B16-Luc tumours did not appear (Fig. 6a, right).

Immunohistochemistry of subcutaneous tissues using anti-CD8 antibody 20 h after injection of naïve C57BL/6 mice with B16-Luc cells revealed minimum CD8^+^ T cells at challenged sites (0.2 cells/high power field (HPF)) (Fig. 6b, left and Fig. 6c). By contrast, significant CD8^+^ cell infiltration (92.2 cells/HPF) was seen at injection sites of B16-Luc cells in C57BL/6 mice that had recovered from B16-Luc brain tumours following IF7C(RR)-SN38 treatment (Fig. 6b, right; Fig. 6c). These results strongly suggest that IF7C(RR)-SN38 therapy not only leads to complete remission from brain tumours but also promotes immunological rejection of tumour cells by the host, preventing tumour recurrence elsewhere in the body.

## Discussion

Here we show that the highly effective tumour vasculature-targeting chemotherapeutic IF7C(RR)-SN38 likely promotes an immune response, leading to activation of cytotoxic T cells against brain tumours. We feel our study strengthens the case for IF7C(RR)-SN38 as a candidate for clinical trials against brain malignancies.

We confirmed our previously reported result,^16,17^ the existence of the Anxa1 N-terminal domain on the surface of tumour vasculature surface in the mouse. However, as reported by others,^23^ this domain is not expressed as a full-length Anxa1 but exist as peptide fragment (Fig. 1a, Supplemental Table 1 and Table 2). We also found that first 15 amino acid residues of this protein are sufficient for IF7 binding (Fig. 2). IF7 bound both human and mouse MC16 peptides equally (Fig. 3a), suggesting that results obtained by IF7 in the mouse tumour model is relevant to humans. Based on these findings, we proposed that an intravenously injected IF7-conjugated compound would be delivered to the tumour vasculature and become concentrated in tumours. Data reported in this (Fig. 3) and previous^16,17^ studies suggest that IF7 and IF7-conjugated chemicals are transported across endothelial cells by transcytosis from the luminal surface to basal stroma. Anxa1-mediated endocytosis/transcytosis has also been reported by Oh et al. in a lung cancer model.^23^ They reported that when Anxa1 expressed on caveoli of endothelial cells was bound by anti-Anxa1 antibody, it became internalised and was transported to the basal surface. We conclude that IF7 binding to some, if not all, Anxa1 fragments containing MC16 domain mediates transcytosis in a manner similar to Anxa1 binding to anti-Anxa1 antibody. We hypothesised that transcytosis of IF7 may enable an intravenously injected IF7-conjugated chemotherapeutic to overcome the BBB and reach brain tumours. When we developed IF7, we added cysteine to the C-terminus for drug conjugation, which we extended by two arginines to increase hydrophilicity (IF7C(RR)). The resulting IFLLWQRCRR sequence resembles R/KXXR/K, a consensus for the C-end rule (CendR) for internalisation and tissue penetration via neutrophin-1.^34,35^ However, we conjugated IF7C(RR) with A488 or SN38 through the C within that motif, which likely inactivates that activity. Instead, we hypothesise that transcytosis of IF7-constructs occurs at caveolae on the endothelial cell surface, as shown for transcytosis of anti-ANXA1 antibody.^23,36^ As multiple BBB transcytosis pathways are known,^37,38^ IF7/ANXA1 may overcome the BBB via multiple strategies.

We reported previously that in model mice harbouring subcutaneous colon tumours, 15 min after intravenous injection of fluorescently labelled IF7, fluorescent signals are seen in vesicles at mostly the basal side of vasculature, while 40 min later, fluorescence is seen in the stroma and in tumour cells.^16^ In our brain tumour model mice, fluorescence-labelled IF7 intravenously injected became concentrated in the tumour at levels 900-fold greater than in surrounding normal brain tissue (Fig. 3c, d). Appearance of IF7 fluorescence in brain tumour stroma is evidence that IF7 crossed the BBB (Fig. 3e). The presence of fluorescent signals in tumour cells also suggests that IF7 is actively taken up by cancer cells, further suggesting that the IF7 receptor is expressed on the surface of brain tumour cells. Our unpublished data suggests that the MC16 domain is expressed not only on the tumour vasculature surface but also on tumour cell surface as well. We hypothesise that IF7 in tumour stroma actively targets Anxa1 peptides on the tumour cell surface exhibiting the MC16 domain. However, our LC-MS/MS analysis did not detect SN38 in brain tumours from mice intravenously injected with IF7C(RR)-SN38, possibly because we administered low levels of IF7-SN38. Nonetheless, we conclude that levels of SN38 that reach tumour cells are high enough to be cytotoxic. As gene expression data indicates *ANXA1* overexpression in brain malignancies^39,40^ and our immunohistochemistry for the Anxa1 N-terminal domain showed strong signals in brain tumours (Fig. 1c, Supplementary Fig. 3), the IF7C(RR)-SN38 conjugate may be clinically relevant to treatment of brain tumours in humans.

We found in this study that IF7C(RR)-SN38 not only suppressed tumour growth but also triggered tumour recognition by the host’s immune system (Fig. 5 and Fig. 6). We speculate that high efficacy of IF7C(RR)-SN38 against the brain tumour allowed the undamaged host’s immune cells to infiltrate the tumour and recognise antigens displayed by partially damaged cancer cells. Emergence of cytotoxic CD8 + T cells against a tumour in vivo leads to tumour eradication, and observations reported here suggest that IF7C(RR)-SN38 may induce an immunotherapeutic response by the host. In PD-1/PD-L1 blocking therapy, tumour infiltration by cytotoxic T cells is critical for clinical success.^41^ In a successful anti-PD-1 antibody therapy on melanoma patients, CD8 + T cells infiltrated melanoma tumours.^42^ In a mouse ovarian tumour model, T-cell infiltration of tumours was induced in response to PD-L1 blocking therapy.^43^ Highly selective cytotoxicity of IF7C(RR)-SN38 to cancer cells may allow an intact inflammatory T-cell response to brain tumours. Although brain tissue was thought to be immunologically privileged, recent studies show that antigens in brain induce immune responses.^44^ Thus, development of IF7C(RR)-SN38 could extend application of checkpoint immunotherapies to patients with brain malignancies.

This study suggests clinical relevance of IF7C(RR)-SN38 by following reasons. First, IF7 binds to human and mouse MC16 (Fig. 3a), suggesting that that results obtained in the mouse brain tumour models will be applicable to human brain tumours. Second, our monoclonal anti-MC16 antibody reacted positively with human tumour vasculatures including brain tumours (Fig. 1c and Supplemental Figs. 2, 3). Finally, analyses of gene expression in human cancer specimens indicates that *ANXA1* is overexpressed in CNS tumours, particularly in glioblastoma.^39,40,45^

As the Anxa1 N-terminus exerts various anti-inflammatory effects,^46–48^ we assume that IF7(RR)-SN38 antagonises these effects and thus increases inflammation, as described in Fig. 6. However, our histological analysis of tumours from mice injected with IF7 did not reveal signs of inflammation (data not shown). Since Anxa1 is expressed on the cell surface following activation of macrophages^49^ or after apoptosis of many cell types,^50,51^ IF7-SN38 therapy may promote unknown effects by targeting activated macrophages or apoptotic cells. Further study of IF7C(RR)-conjugated drugs is needed to assess these possibilities. Moreover, although we recognise that it would be ideal to analyse many of these phenotypes in *Anxa1*-deficient mutant mice, we were unable to pursue this strategy because tumours do not grow well in these mice, due to impaired tumour angiogenesis, as we reported previously.^17^

Present study showed mice received daily injection of IF7C(RR)-SN38 at 2.5 μmoles (5.35 mg)/kg led to complete disappearance of brain tumours (Fig. 5a). This dosage in mouse translates into a human equivalent dose (HED) of 0.43 mg/kg.^52^ Thus, we may see signs of a therapeutic effect in humans when the dose is escalated to 0.43 mg/kg. Recommended CPT-11 dosages to human cancer patients are 120–200 mg/m^2^.^53,54^ Assuming that body surface area of normal adult is 1.7 m^2^ and body weight is 70 kg, these values are calculated to be 2.91–4.85 mg/kg. Given that CPT-11 molecular weight (MW) is 586 Da and that of SN38 is 392 Da, these recommended dosages of CPT-11 are equivalent at SN38 1.95–3.24 mg/kg. IF7C(RR)-SN38 (2141 Da) 0.43 mg contains 0.079 mg SN38. These values of IF7C(RR)-SN38 predict few SN38-associated toxicities in humans at pharmacologically active dose.

Cancer treatments are becoming more expensive due to development of increasingly sophisticated diagnostics and therapies. Our drug, which consists of a short peptide plus an anticancer reagent, can be chemically synthesised cost effectively. Given the predicted extremely efficient tumour targeting activity, IF7C(RR)-SN38 should be effective at low dosage, minimising side effects and further reducing costs. As clinical trials with tumour vasculature homing peptides are beginning,^55,56^ we will soon be able to evaluate these strategies for efficacy in cancer patients, including brain malignancies.

## Supplementary information

Supplemental figures

Supplementary Table 1

Supplemental Table 2

## Data Availability

All data supporting the results reported in the article are available upon request.
