# Supplementary material for: Overcoming the blood–brain barrier by Annexin A1-binding peptide to target brain tumours
Source: Br J Cancer. 2020 Sep 14;123(11):1633–43. doi: 10.1038/s41416-020-01066-2 (PMC7686308; doi:10.1038/s41416-020-01066-2)
Supplement: Supplementary file 1 — Supplemental figures [file 41416_2020_1066_MOESM1_ESM.pdf]

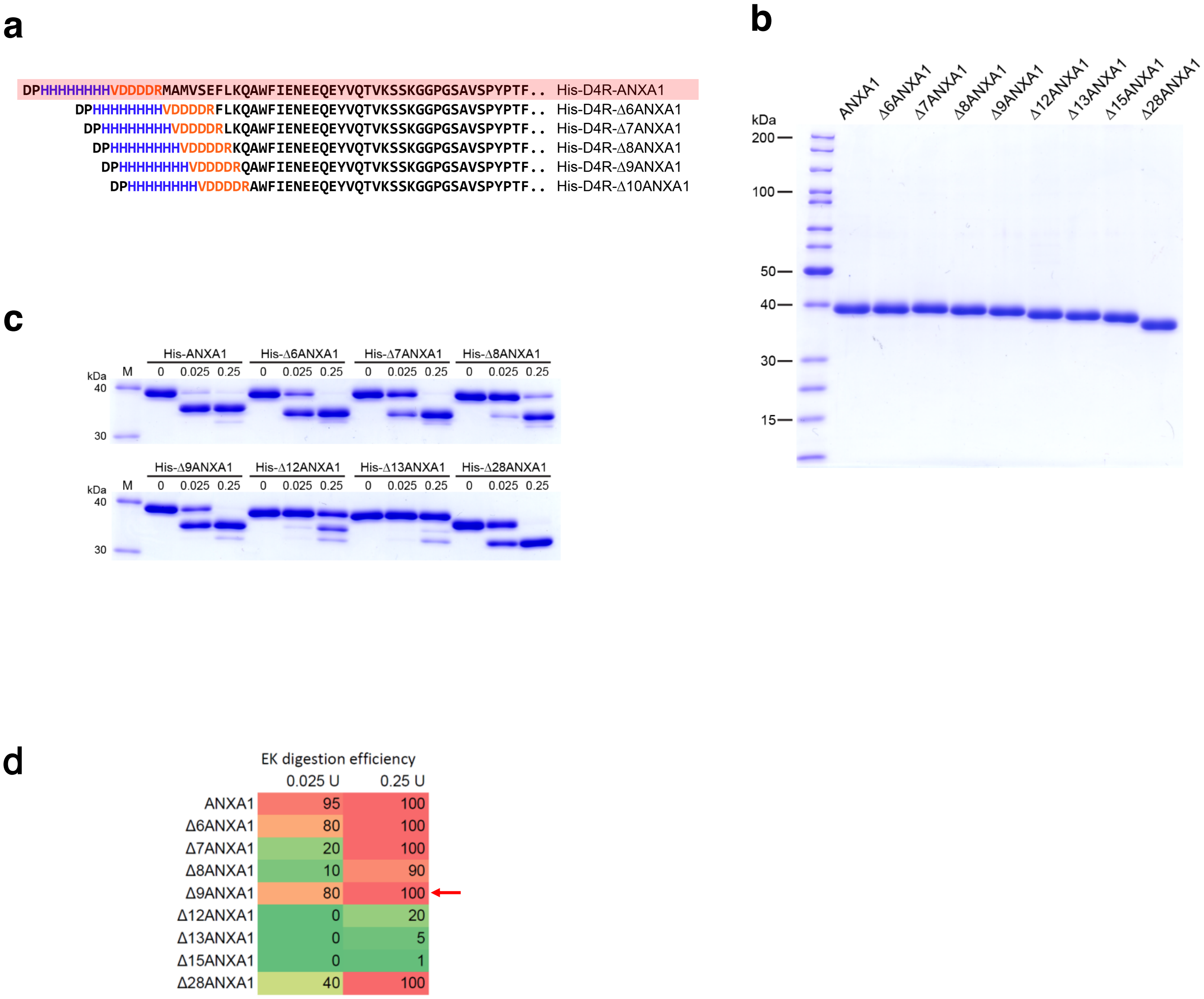

Supplementary Fig. 1. Purification of full-length ANXA1 and N-terminal deletion mutants expressed in insect cells using a baculovirus expression system. Methods described by Jarvis *(3)* adopted. (**a**) Schematic representation of N-terminal region of ANXA1peptide sequences for full-length protein and deletion mutants (Δ4~Δ28). Recombinant ANXA1 proteins were expressed and purified as described in Extended Data Materials and Methods. (**b**) Coomassie blue-stained SDS-polyacrylamide gel of indicated purified His_8-tagged proteins. Recombinant proteins were purified by Ni-affinity chromatography. (**c**) Digestion by enterokinase (EK) of purified proteins. (**d**) Heat map of EK digestion efficiency. Analysis indicates that the Δ9 mutant (red arrow) is the longest deletion susceptible to EK.

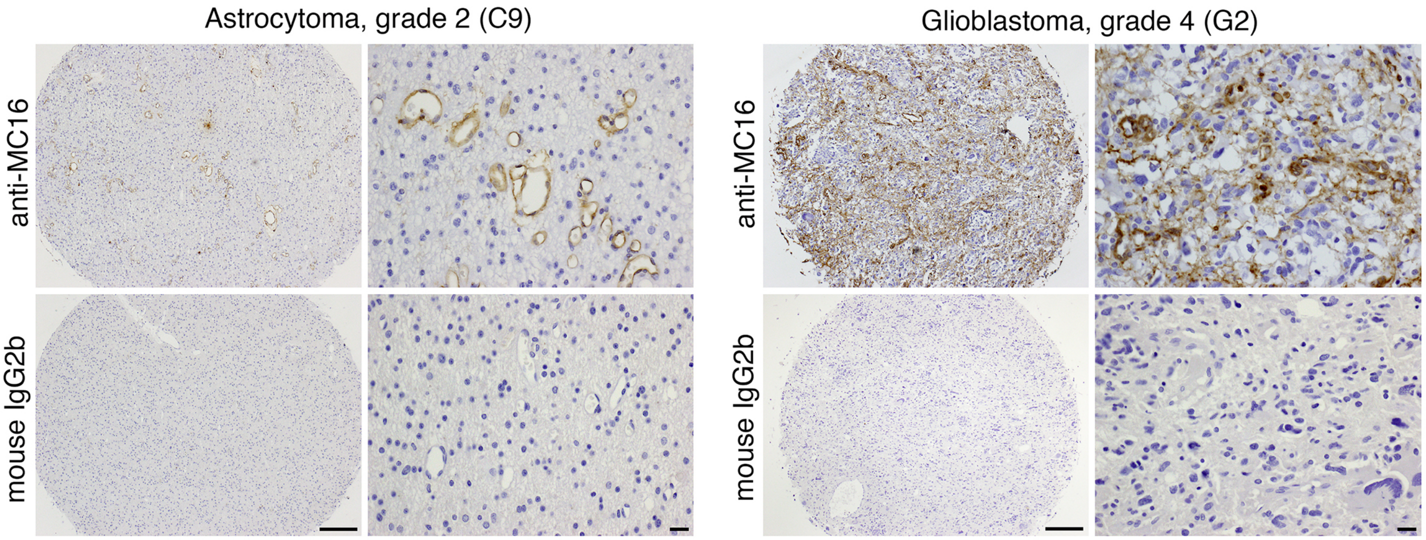

Supplementary Fig. 2. Immunohistochemistry of brain tumors with anti-MC16 antibody and control mouse IgG2b protein. Shown are brain tumor tissue sections on tissue microarray slides, US Biomax GL807a core C9 (astrocytoma) and core G2 (glioblastoma), stained as indicated. Note no specific staining on control isotype-matched mouse IgG2b-stained slides. Scale bars in low- and high-magnifications are 200 μm and 20 μm, respectively.

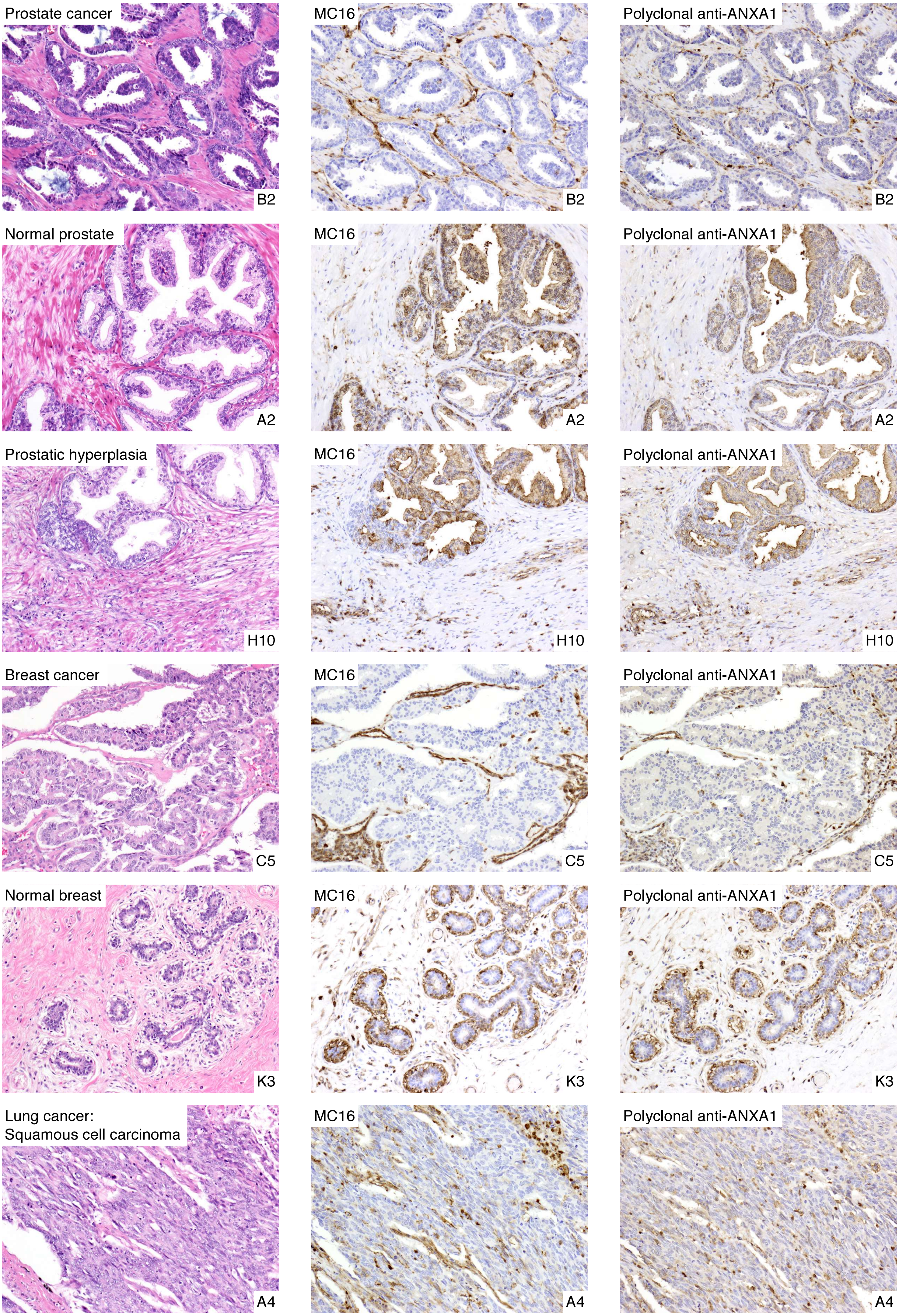
Prostate cancer
MC16
Polyclonal anti-ANXA1
B2
Normal prostate
A2
Prostatic hyperplasia
H10
Breast cancer
C5
Normal breast
K3
Lung cancer:
Squamous cell carcinoma
A4

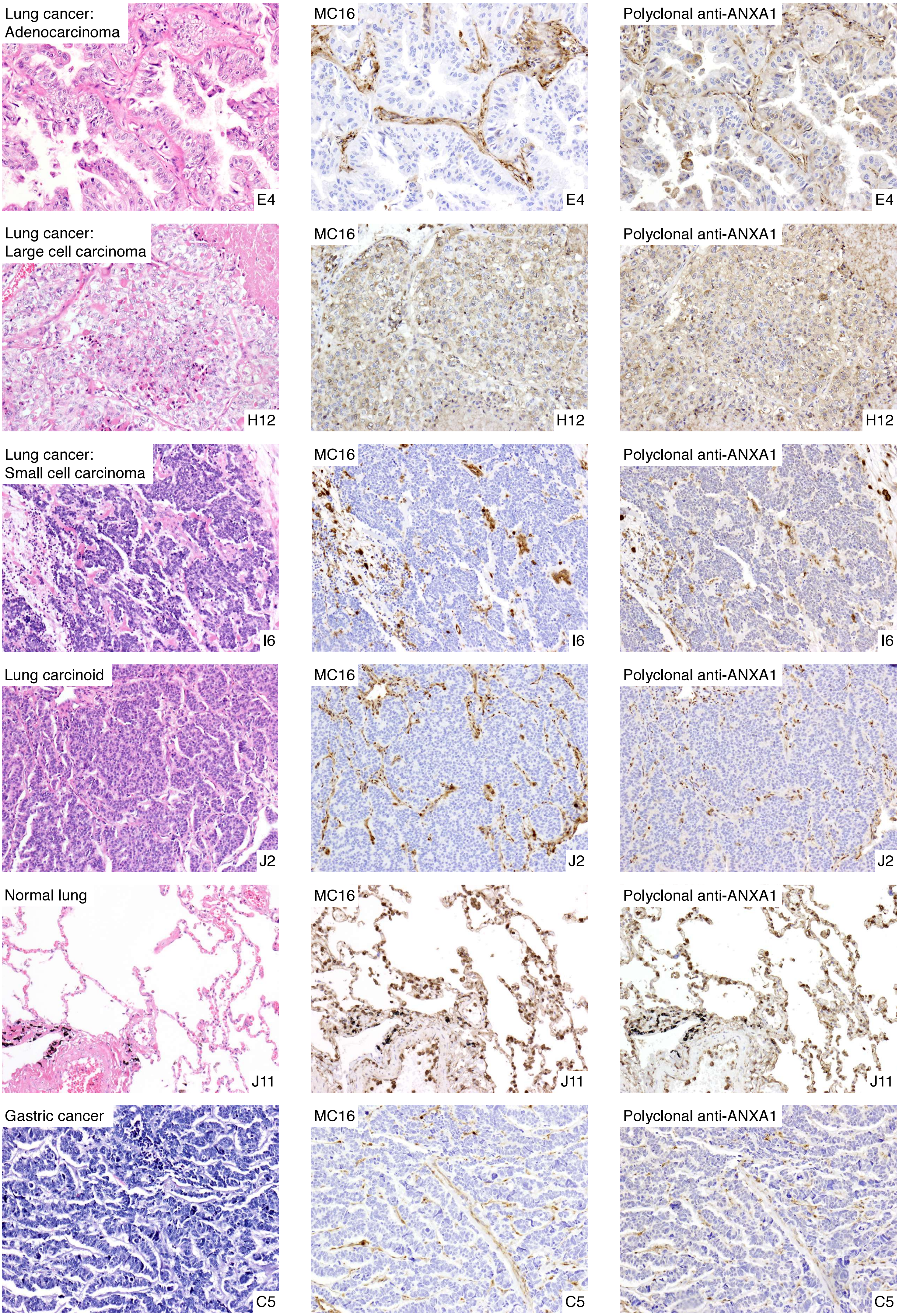

Lung cancer: Adenocarcinoma
MC16
Polyclonal anti-ANXA1
E4
E4
E4
Lung cancer: Large cell carcinoma
MC16
Polyclonal anti-ANXA1
H12
H12
H12
Lung cancer: Small cell carcinoma
MC16
Polyclonal anti-ANXA1
I6
I6
I6
Lung carcinoid
MC16
Polyclonal anti-ANXA1
J2
J2
J2
Normal lung
MC16
Polyclonal anti-ANXA1
J11
J11
J11
Gastric cancer
MC16
Polyclonal anti-ANXA1
C5
C5
C5

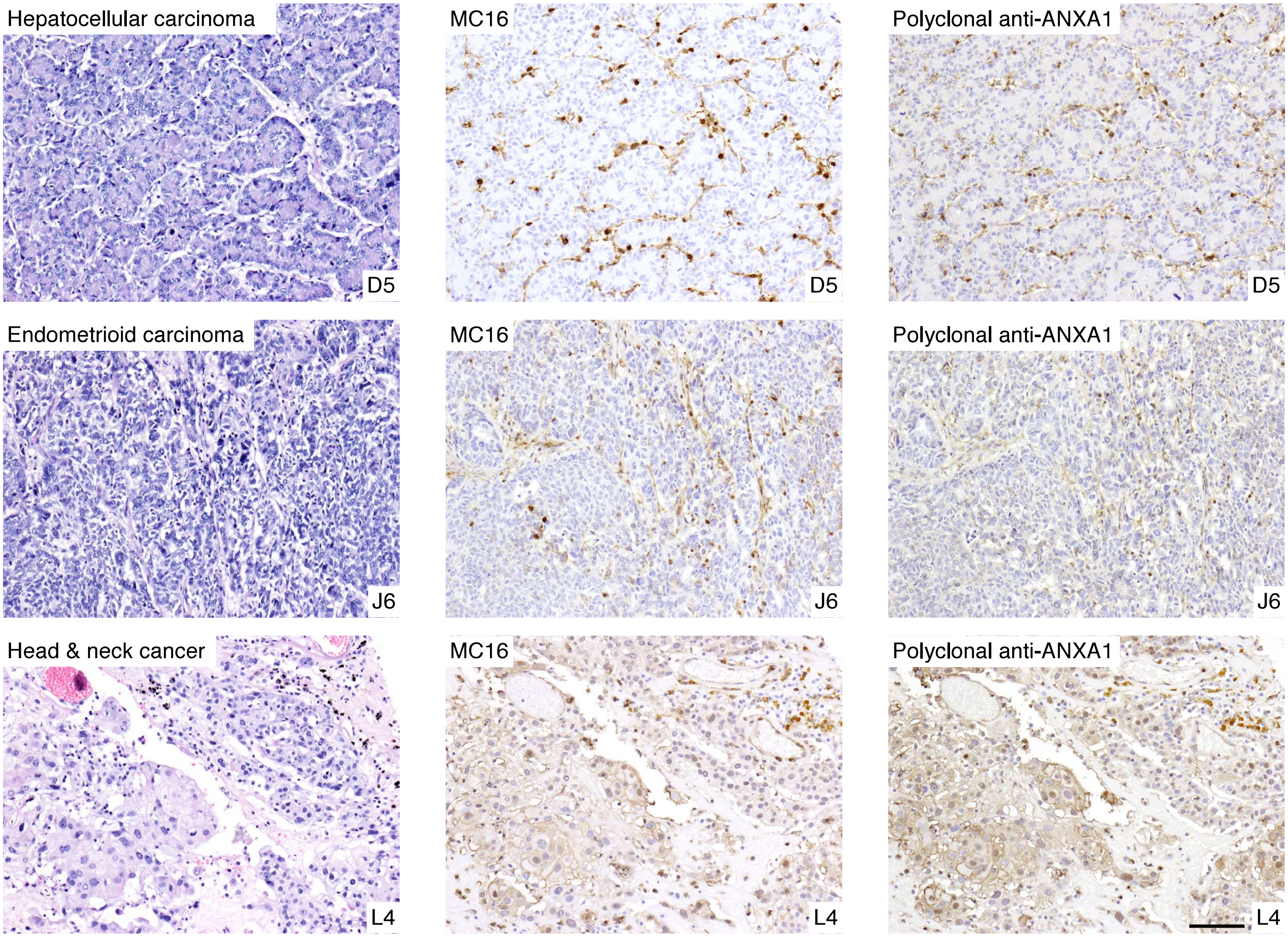

Supplementary Fig. 3. Immunohistochemistry of indicated tumor types using anti-MC16 versus polyclonal rabbit anti-ANXA1 antibodies. Cancer tissue microarray slides from US Biomax were stained with H and E (left panels), with an anti-MC16 antibody generated here (middle panels) or with a polyclonal rabbit anti-ANXA1 antibody (Invitrogen, 71-3400). Tissue ID numbers for each microarray are indicated at lower right corner. Some images overlap with those shown in main Fig. 1C. Scale bar=100 μm.

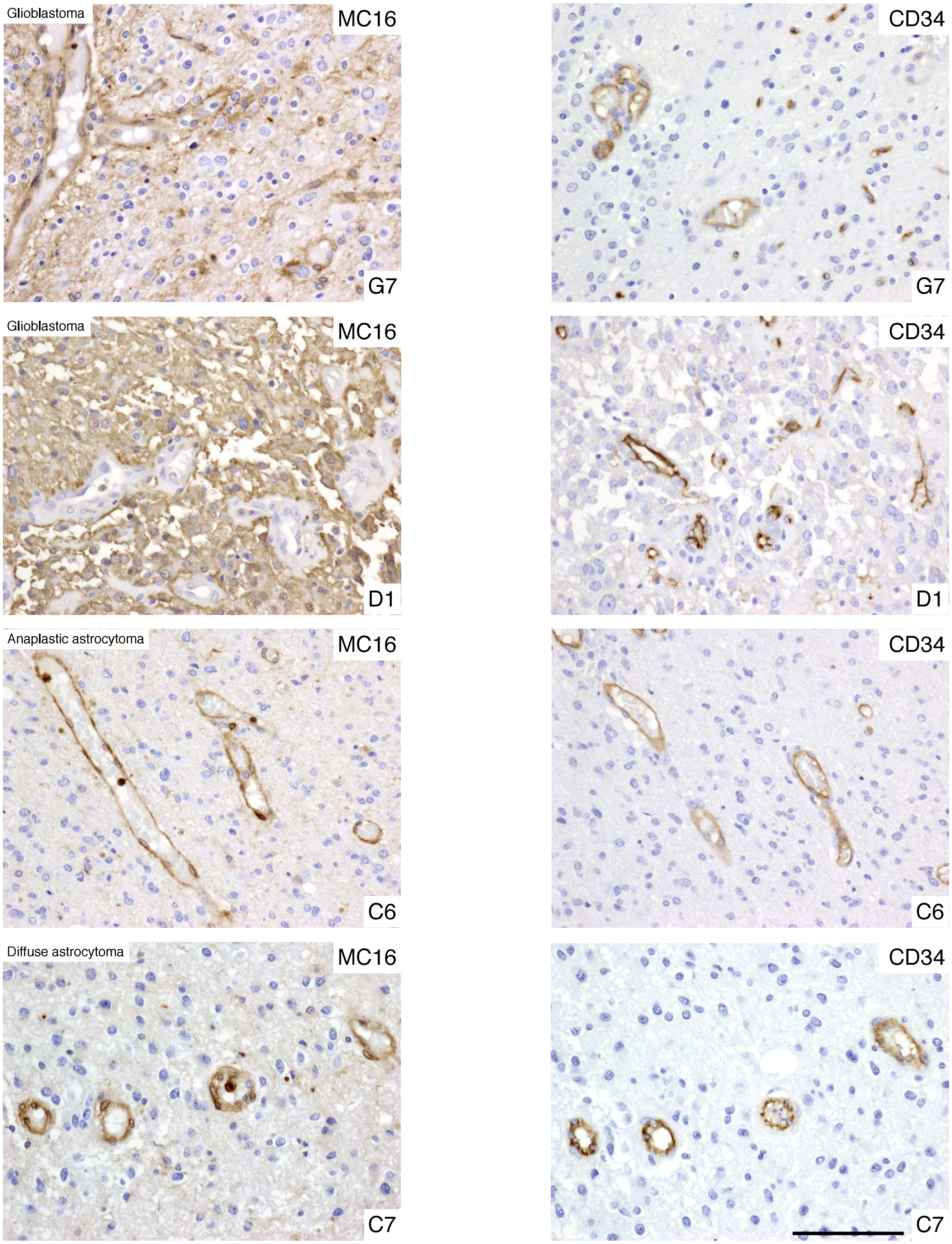

Supplementary Fig. 4. Immunohistochemistry of brain tumor specimens. Tissue microarray slides (GL806e) for various brain tumors stained with anti-MC16 antibody (left panels) and mouse monoclonal anti-CD34 (a vascular marker, QBEnd-10, Agilent, Santa Clara, CA, USA) (right panels). Tissue ID numbers in the microarray are indicated in the lower right corner. Scale bar=100 μm.

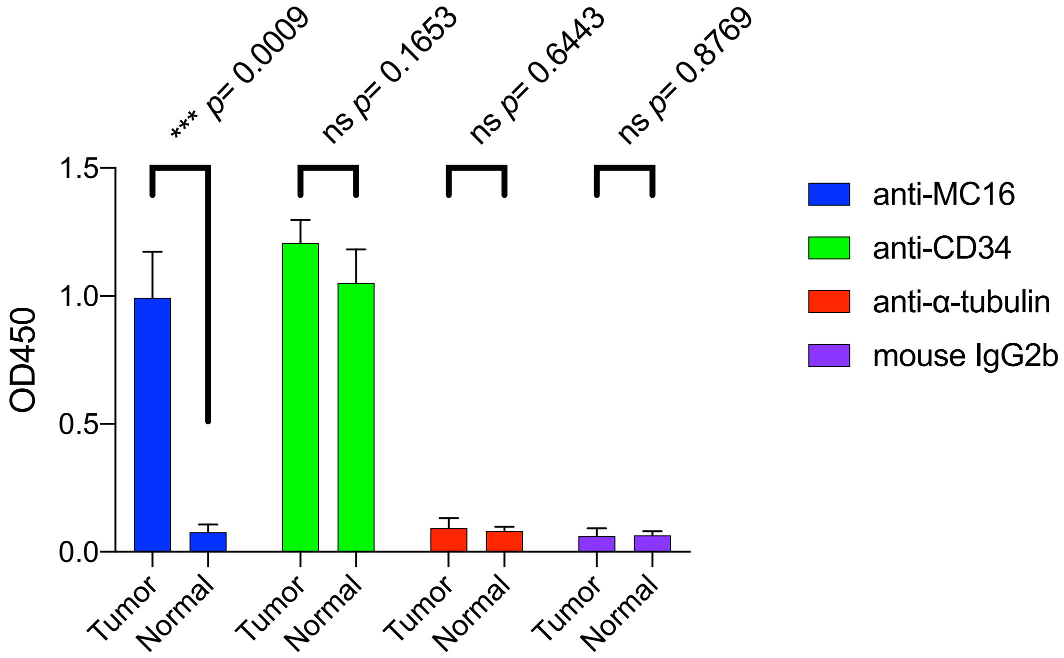

Supplementary Fig. 5. Detection of MC16 antigen on the vasculature surface in subcutaneous B16 tumors and normal subcutaneous tissue. Three tumor bearing mice were subjected to *in vivo* biotinylation procedures followed as those described in Material and Methods and main body Fig. 1E. Solubilized proteins (3 mg from tumor and adjacent normal subcutaneous tissues from three mice) were IP'd using anti-MC16 antibody, or one of three control antibodies, anti-CD34 antibody for vasculature surface membrane protein, and anti-α-tubulin antibody for soluble cytoplasmic protein, and isotype matched mouse IgG2b, and then treated with protein A-bound magnetic beads. Biotinylated proteins in captured immunoprecipitates were detected using peroxidase-conjugated avidin followed by a peroxidase color reaction. Error bars denote means ± SEM. Statistical analysis was assessed by Student's t-test.

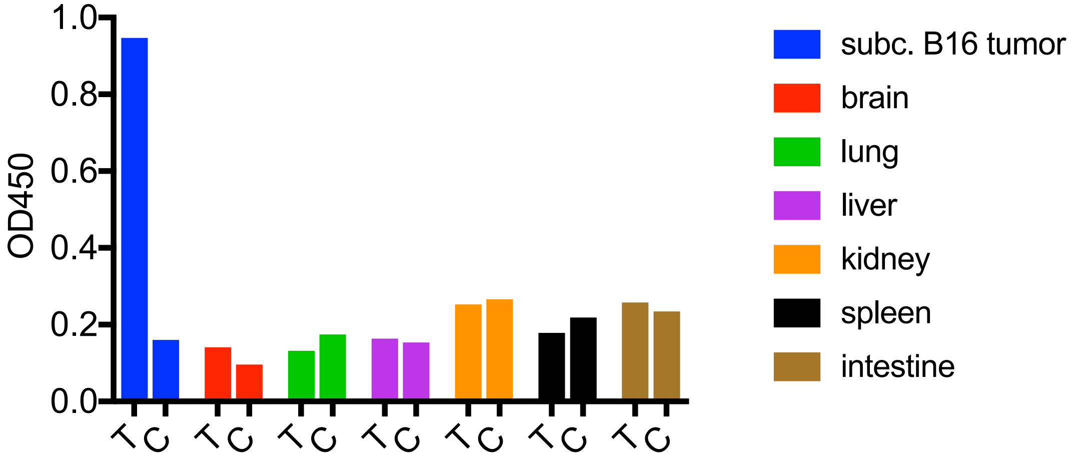

Supplementary Fig. 6. Detection of MC16 antigen on the vasculature surface in subcutaneous B16 tumors and normal tissues in mouse. A tumor-bearing mouse generated by subcutaneous injection of B16 cells into C57BL/6 mice was biotinylated *in vivo* by intravenous injection with NHS-LC-biotin. Fifteen minutes later, the mouse was perfused with PBS through the heart, and tissues isolated and homogenized with PBS containing 1% NP-40. Solubilized proteins, 3 mg from each homogenate, were subjected to immunoprecipitation by anti-MC16 antibody (T) or isotype matched mouse IgG2b (C) using protein A-bound magnetic beads. Biotinylated proteins in immunoprecipitates were measured by reacting beads with peroxidase-conjugated avidin followed by a peroxidase color reaction.

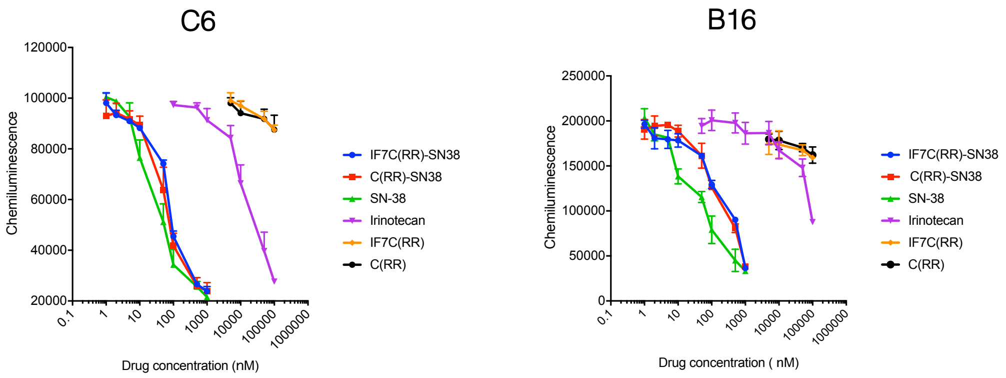

C6	IF7C(RR)-SN38	C(RR)-SN38	SN-38	Irinotecan	IF7C(RR)	C(RR)
IC50	64.84	56.09	27.13	12691	N.D.	N.D.
logIC50	1.812	1.749	1.433	4.103	N.D.	N.D.
R square	0.9767	0.9787	0.9783	0.9689	N.D.	N.D.

B16	IF7C(RR)-SN38	C(RR)-SN38	SN-38	Irinotecan	IF7C(RR)	C(RR)
IC50	148.1	139.2	31.57	27726	N.D.	N.D.
logIC50	2.171	2.144	1.499	4.443	N.D.	N.D.
R square	0.938	0.9687	0.9612	0.8401	N.D.	N.D.

Supplementary Fig 7. Cytotoxicity of peptide-conjugated SN38, free peptides and unconjugated SN-38 and irinotecan (CPT-11) on C6 and B16 cells cultured *in vitro*. Rat glioma C6 and mouse melanoma B16 cells were treated with reagents shown along the top panels at the concentration indicated and cultured for 2 days. Cell viability was determined by a CellTiter Glo (Promega) assay. The IC50 of each reagent was determined using GraphPad Prism program. N.D.: not determined.

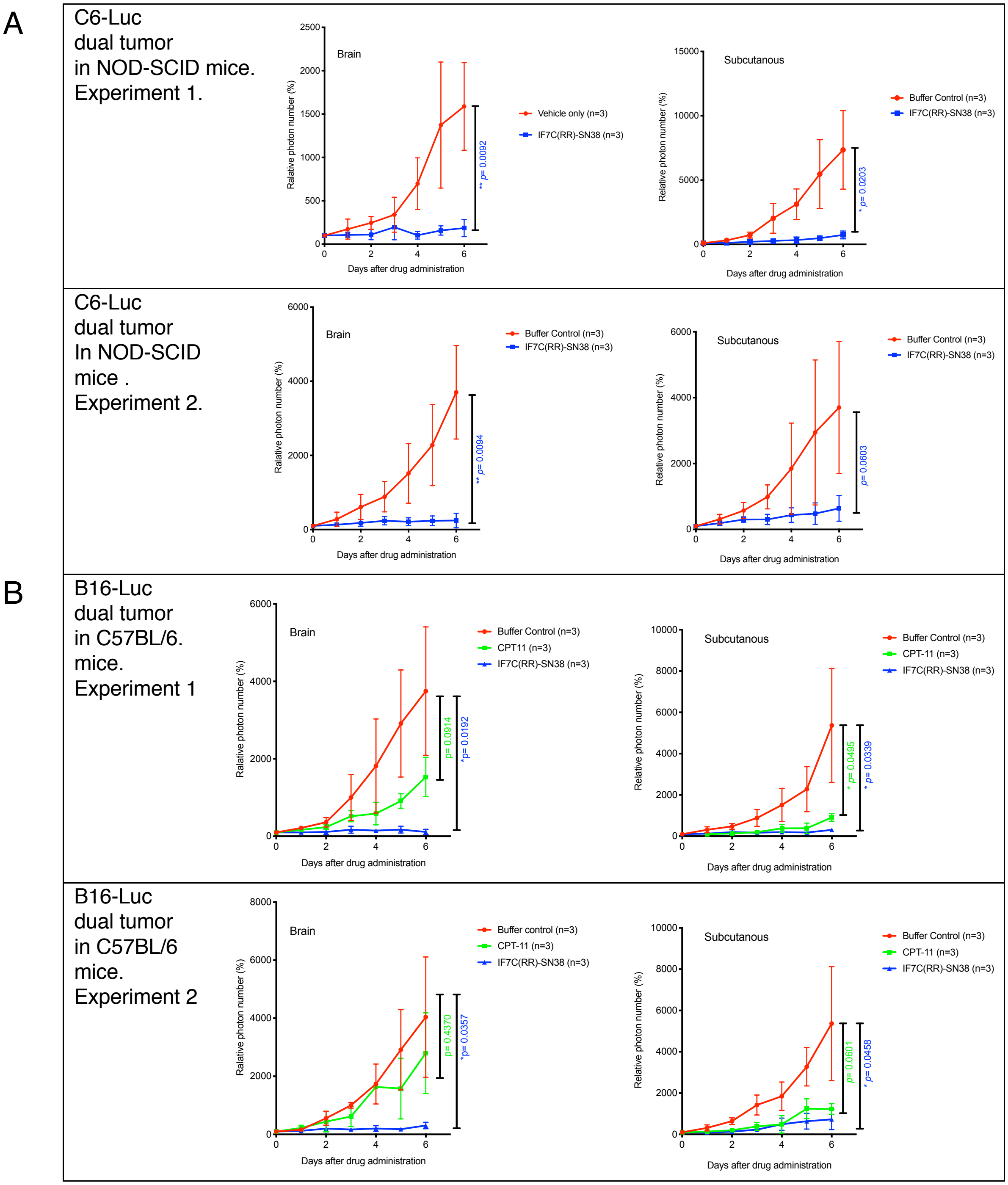

Supplementary Fig. 8. Effect of IF7C(RR)-SN38 treatment in dual tumor mouse models. Dual tumor model was established with C6-Luc cells in NOD-SCID mice (**A**) and B16-Luc in C57BL/6 mice (**B**). When tumors in brain and subcutaneous grew, IF7C(RR)-SN38 (3.15 µmoles/kg dissolved with Cremophore EL) was injected intravenously daily through tail vein for 7 days. In each graph, error bars denote means ± SEM. Statistical analysis was assessed by Student's t-test. Note that effect of IF7C(RR)-SN38 on brain tumors is comparable to that on subcutaneous tumors regardless of tumor cell line and mouse strain. Each pair represents additional data (total three experiments) presented relevant to main Fig. 4.

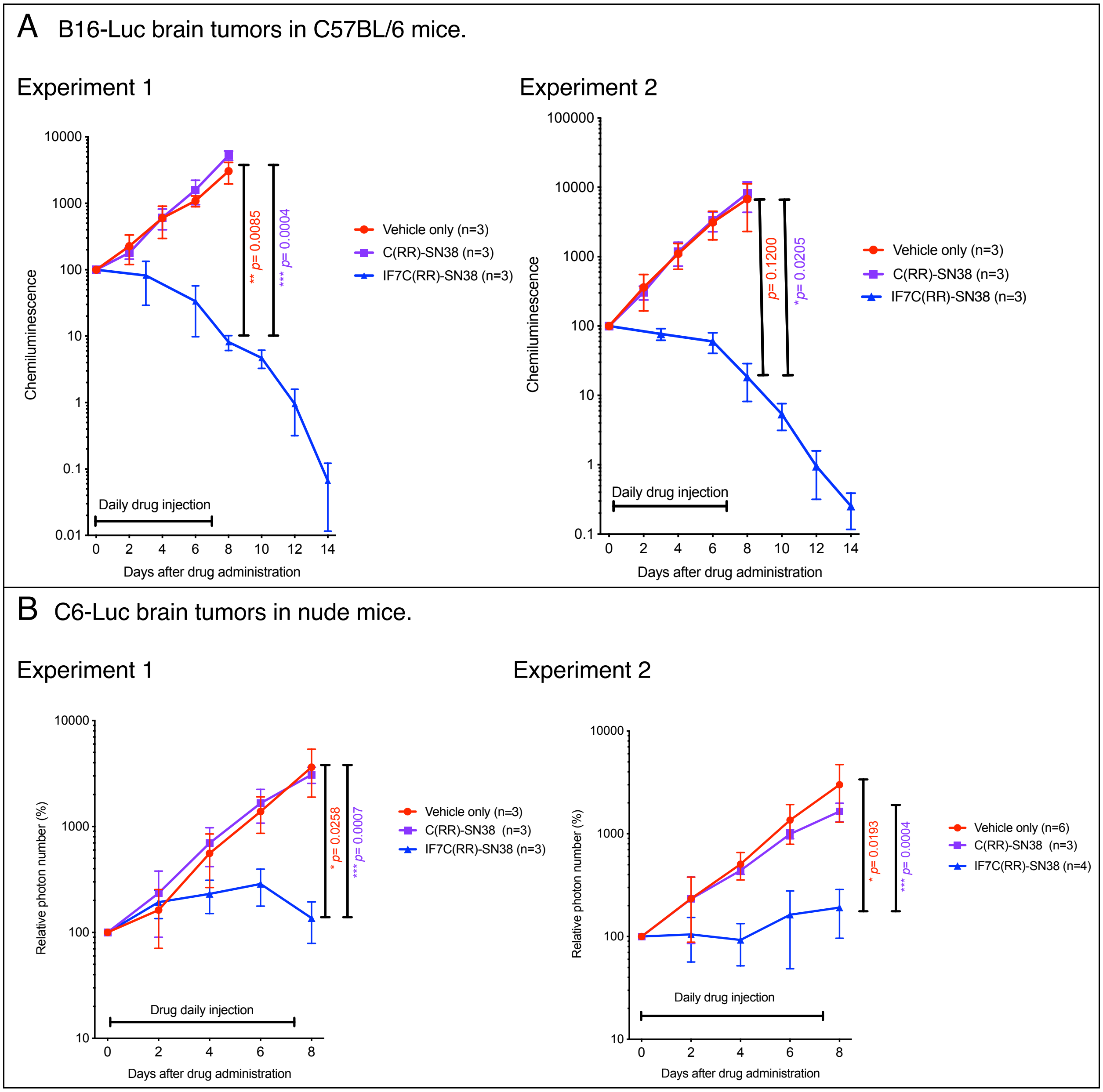

Supplementary Fig. 9. Effect of treatment with IF7C(RR)- SN38 formulated with 10% Solutol HS15 on brain tumors. Effect of IF7C(RR)-SN38 on B16-Luc brain tumors in C57BL/6 mice (**A**) and that on C6-Luc brain tumors in nude mice (**B**). In these experiments, drug dosage was 2.5 µmoles/kg each for IF7C(RR)-SN38 or C(RR)-SN38 (control), diluted with 10% Solutol HS15 in water and administered daily for 7 days. Note that the B16 brain tumors continued shrinking after ceasing administration of IF7C(RR)-SN38 in C57BL/6 mice. In each graph, error bars denote means ± SEM. Statistical analysis was assessed by Student's t-test. Each set of figures represents additional data presented relevant to main Fig. 5.
