# Supplementary material for: Overcoming the blood–brain barrier by Annexin A1-binding peptide to target brain tumours
Source: Br J Cancer. 2020 Sep 14;123(11):1633–43. doi: 10.1038/s41416-020-01066-2 (PMC7686308; doi:10.1038/s41416-020-01066-2)
Supplement: Supplementary file 2 — Supplementary Table 1 [file 41416_2020_1066_MOESM2_ESM.pdf]

| Protein IDs | Majority protein IDs | Protein names | Gene names | Intensity | MS/MS count | Potential contaminant | Fasta headers | Number of proteins | Peptide counts (all) |
|---|---|---|---|---|---|---|---|---|---|
| P04083 | P04083 | Annexin A1 | ANXA1 | 2.46E+10 | 206 | | Annexin A1 ( | 1 | 30 |
| P69905;P02008 | P69905 | Hemoglobin subunit | HBA1 | 1.44E+09 | 34 | | Hemoglobin | 2 | 8;1 |
| P63261;P60709;P63267 | P63261;P60709;P63267 | Actin, cytoplasmic 2 | ACTG1;ACTB;A( | 2.98E+08 | 29 | | Actin, cytopla | 12 | 9;9;5;5;5;5;4; |
| P62269 | P62269 | 40S ribosomal prote | RPS18 | 2.77E+08 | 18 | | 40S ribosoma | 1 | 7 |
| P14618 | P14618 | Pyruvate kinase PKM | PKM | 2.51E+08 | 35 | | Pyruvate kina | 1 | 16 |
| Q16555 | Q16555 | Dihydropyrimidinase | DPYSL2 | 2.38E+08 | 57 | | Dihydropyrim | 1 | 14 |
| P11142;P54652;P34931 | P11142 | Heat shock cognate | HSPA8 | 1.98E+08 | 36 | | Heat shock c | 5 | 20;7;2;1;1 |
| P15104 | P15104 | Glutamine syntheta | GLUL | 1.84E+08 | 13 | | Glutamine sy | 1 | 4 |
| Q5VTE0;P68104 | Q5VTE0;P68104 | Putative elongation | EEF1A1P5;EEF1 | 1.73E+08 | 35 | | Putative elor | 2 | 7;7 |
| P60174 | P60174 | Triosephosphate iso | TPI1 | 1.29E+08 | 4 | | Triosephosph | 1 | 4 |
| P08559;P29803 | P08559 | Pyruvate dehydroge | PDHA1 | 1.26E+08 | 20 | | Pyruvate deh | 2 | 12;4 |
| P30626 | P30626 | Sorcin | SRI | 1.25E+08 | 17 | | Sorcin OS=Hc | 1 | 7 |
| P07355;A6NMY6 | P07355;A6NMY6 | Annexin A2;Putative | ANXA2;ANXA2P | 1.16E+08 | 14 | | Annexin A2 ( | 2 | 9;7 |
| P02042 | P02042 | Hemoglobin subunit | HBD | 1.12E+08 | 6 | | Hemoglobin | 1 | 3 |
| P34932;O95757 | P34932 | Heat shock 70 kDa p | HSPA4 | 1.11E+08 | 26 | | Heat shock 7 | 2 | 16;3 |
| P51659 | P51659 | Peroxisomal multifu | HSD17B4 | 1.03E+08 | 11 | | Peroxisomal | 1 | 7 |
| P12277 | P12277 | Creatine kinase B-ty | CKB | 97581000 | 12 | | Creatine kina | 1 | 6 |
| P36543;Q96A05 | P36543 | V-type proton ATPas | ATP6V1E1 | 87174000 | 9 | | V-type protoı | 2 | 7;3 |
| Q92734 | Q92734 | Protein TFG | TFG | 83232000 | 10 | | Protein TFG ( | 1 | 7 |
| P40926 | P40926 | Malate dehydrogena | MDH2 | 70780000 | 8 | | Malate dehy | 1 | 7 |
| P04075 | P04075 | Fructose-bisphospha | ALDOA | 62929000 | 8 | | Fructose-bis | 1 | 5 |
| P62873;P62879;Q9HAV | P62873 | Guanine nucleotide- | GNB1 | 57476000 | 11 | | Guanine nucl | 4 | 7;3;2;1 |
| P30050 | P30050 | 60S ribosomal prote | RPL12 | 57198000 | 8 | | 60S ribosoma | 1 | 5 |
| P39019 | P39019 | 40S ribosomal prote | RPS19 | 54563000 | 7 | | 40S ribosoma | 1 | 6 |
| O14556 | O14556 | Glyceraldehyde-3-pl | GAPDHS | 48439000 | 6 | | Glyceraldehy | 1 | 2 |
| P62805 | P62805 | Histone H4 | HIST1H4A | 47679000 | 7 | | Histone H4 C | 1 | 4 |
| P10606 | P10606 | Cytochrome c oxidas | COX5B | 47646000 | 5 | | Cytochrome ( | 1 | 3 |
| P61158;Q9P1U1 | P61158 | Actin-related protei | ACTR3 | 45493000 | 10 | | Actin-relatec | 2 | 9;1 |
| P05091 | P05091 | Aldehyde dehydroge | ALDH2 | 44238000 | 7 | | Aldehyde del | 1 | 5 |
| P62241 | P62241 | 40S ribosomal prote | RPS8 | 38383000 | 10 | | 40S ribosoma | 1 | 5 |
| P62701;Q8TD47;P2209 | P62701 | 40S ribosomal prote | RPS4X | 38004000 | 13 | | 40S ribosoma | 3 | 6;1;1 |
| P21291 | P21291 | Cysteine and glycine | CSRP1 | 37991000 | 11 | | Cysteine and | 1 | 7 |
| P62263 | P62263 | 40S ribosomal prote | RPS14 | 37845000 | 4 | | 40S ribosoma | 1 | 2 |
| P84103 | P84103 | Serine/arginine-rich | SRSF3 | 36756000 | 4 | | Serine/argini | 1 | 4 |

| | | | | | | | | |
|---|---|---|---|---|---|---|---|---|
| P17066;P48741 | P17066;P48741 | Heat shock 70 kDa p | HSPA6;HSPA7 | 35579000 | 1 | Heat shock 7 | 2 | 3;2 |
| P26373 | P26373 | 60S ribosomal prote | RPL13 | 34932000 | 2 | 60S ribosom: | 1 | 2 |
| P42766 | P42766 | 60S ribosomal prote | RPL35 | 34924000 | 2 | 60S ribosom: | 1 | 1 |
| P11021 | P11021 | 78 kDa glucose-regu | HSPA5 | 34367000 | 12 | 78 kDa gluco | 1 | 12 |
| Q99798 | Q99798 | Aconitate hydratase | ACO2 | 34188000 | 7 | Aconitate hy | 1 | 6 |
| P62316 | P62316 | Small nuclear ribonu | SNRPD2 | 33476000 | 7 | Small nuclea | 1 | 4 |
| P00492 | P00492 | Hypoxanthine-guani | HPRT1 | 32520000 | 3 | Hypoxanthine | 1 | 3 |
| Q08209 | Q08209 | Serine/threonine-pr | PPP3CA | 32322000 | 10 | Serine/threo | 1 | 7 |
| P62851 | P62851 | 40S ribosomal prote | RPS25 | 31934000 | 4 | 40S ribosom: | 1 | 4 |
| P17600 | P17600 | Synapsin-1 | SYN1 | 31701000 | 13 | Synapsin-1 C | 1 | 6 |
| P15924 | P15924 | Desmoplakin | DSP | 30273000 | 22 | Desmoplakin | 1 | 19 |
| Q00839 | Q00839 | Heterogeneous nucl | HNRNPU | 29368000 | 8 | Heterogeneo | 1 | 5 |
| P09622 | P09622 | Dihydrolipoyl dehydı | DLD | 29171000 | 6 | Dihydrolipoyl | 1 | 4 |
| P46779 | P46779 | 60S ribosomal prote | RPL28 | 28144000 | 2 | 60S ribosom: | 1 | 2 |
| O75340 | O75340 | Programmed cell de | PDCD6 | 28082000 | 6 | Programmec | 1 | 3 |
| P61764 | P61764 | Syntaxin-binding pro | STXBP1 | 27694000 | 11 | Syntaxin-binc | 1 | 9 |
| P62306 | P62306 | Small nuclear ribonu | SNRPF | 27550000 | 20 | Small nuclea | 1 | 2 |
| P04632 | P04632 | Calpain small subun | CAPNS1 | 27250000 | 7 | Calpain smal | 1 | 4 |
| Q06830 | Q06830 | Peroxiredoxin-1 | PRDX1 | 27187000 | 4 | Peroxiredoxi | 1 | 3 |
| P22061 | P22061 | Protein-L-isoasparta | PCMT1 | 27081000 | 7 | Protein-L-iso | 1 | 3 |
| P02686 | P02686 | Myelin basic proteir | MBP | 26575000 | 10 | Myelin basic | 1 | 4 |
| Q15366;Q15365;P5772 | Q15366;Q15365;P5772 | Poly(rC)-binding pro | PCBP2;PCBP1;P | 26293000 | 4 | Poly(rC)-bind | 3 | 4;2;2 |
| P63098;Q96LZ3 | P63098 | Calcineurin subunit | PPP3R1 | 25556000 | 4 | Calcineurin s | 2 | 3;1 |
| Q9BQE3;Q71U36;P683 | Q9BQE3;Q71U36;P683 | Tubulin alpha-1C ch | TUBA1C;TUBA1 | 25124000 | 7 | Tubulin alpha | 8 | 7;7;6;4;3;2;2; |
| P62249 | P62249 | 40S ribosomal prote | RPS16 | 24754000 | 6 | 40S ribosom: | 1 | 3 |
| P11177 | P11177 | Pyruvate dehydroge | PDHB | 24201000 | 4 | Pyruvate deh | 1 | 3 |
| P47755 | P47755 | F-actin-capping prot | CAPZA2 | 23588000 | 6 | F-actin-cappi | 1 | 6 |
| Q9NQ29 | Q9NQ29 | Putative RNA-bindir | LUC7L | 23510000 | 9 | Putative RNA | 1 | 5 |
| Q9Y383 | Q9Y383 | Putative RNA-bindir | LUC7L2 | 23284000 | 4 | Putative RNA | 1 | 4 |
| P25705 | P25705 | ATP synthase subun | ATP5A1 | 22727000 | 12 | ATP synthase | 1 | 9 |
| Q07955 | Q07955 | Serine/arginine-rich | SRSF1 | 22630000 | 8 | Serine/argini | 1 | 5 |
| P0DN76;Q01081 | P0DN76;Q01081 | Splicing factor U2AF | U2AF1 | 22624000 | 7 | Splicing facto | 2 | 3;3 |
| P04040 | P04040 | Catalase | CAT | 21987000 | 3 | Catalase OS= | 1 | 3 |
| P35268 | P35268 | 60S ribosomal prote | RPL22 | 21311000 | 6 | 60S ribosom: | 1 | 3 |
| Q14195 | Q14195 | Dihydropyrimidinase | DPYSL3 | 21018000 | 6 | Dihydropyrim | 1 | 8 |
| P07195 | P07195 | L-lactate dehydroge | LDHB | 20824000 | 2 | L-lactate deh | 1 | 2 |
| P04406 | P04406 | Glyceraldehyde-3-pl | GAPDH | 20628000 | 13 | Glyceraldehy | 1 | 5 |
| P30084 | P30084 | Enoyl-CoA hydratase | ECHS1 | 20563000 | 5 | Enoyl-CoA hy | 1 | 4 |

| | | | | | | | | | |
|---|---|---|---|---|---|---|---|---|---|
| P48047 | P48047 | ATP synthase subun | ATP5O | 20402000 | 2 | ATP synthase | 1 | | 2 |
| Q86X55 | Q86X55 | Histone-arginine me | CARM1 | 20220000 | 5 | Histone-argi | 1 | | 5 |
| Q13148 | Q13148 | TAR DNA-binding pr | TARDBP | 20214000 | 7 | TAR DNA-bir | 1 | | 6 |
| P06576 | P06576 | ATP synthase subun | ATP5B | 20205000 | 9 | ATP synthase | 1 | | 8 |
| P62753 | P62753 | 40S ribosomal prote | RPS6 | 20080000 | 4 | 40S ribosom | 1 | | 3 |
| P31040 | P31040 | Succinate dehydroge | SDHA | 19707000 | 3 | Succinate de | 1 | | 2 |
| P84077;P61204;P84085 | P84077;P61204;P84085 | ADP-ribosylation fac | ARF1;ARF3;ARF | 19428000 | 4 | ADP-ribosyla | 3 | 4;4;2 | |
| P23528;Q9Y281 | P23528 | Cofilin-1 | CFL1 | 19229000 | 4 | Cofilin-1 OS= | 2 | 3;1 | |
| Q01130 | Q01130 | Serine/arginine-rich | SRSF2 | 19182000 | 3 | Serine/argini | 1 | | 2 |
| P63104 | P63104 | 14-3-3 protein zeta/ | YWHAZ | 19105000 | 6 | 14-3-3 protei | 1 | | 4 |
| P07954 | P07954 | Fumarate hydratase | FH | 19016000 | 7 | Fumarate hy | 1 | | 4 |
| P08621 | P08621 | U1 small nuclear rib | SNRNP70 | 17973000 | 3 | U1 small nuc | 1 | | 2 |
| Q13162 | Q13162 | Peroxiredoxin-4 | PRDX4 | 17666000 | 1 | Peroxiredoxi | 1 | | 1 |
| Q99536 | Q99536 | Synaptic vesicle me | VAT1 | 16442000 | 5 | Synaptic vesi | 1 | | 5 |
| P22695 | P22695 | Cytochrome b-c1 co | UQCRC2 | 16414000 | 2 | Cytochrome | 1 | | 2 |
| Q99878;Q96KK5;Q9BTI | Q99878;Q96KK5;Q9BTI | Histone H2A type 1- | HIST1H2AJ;HIST | 16203000 | 2 | Histone H2A | 13 | 1;1;1;1;1;1;1; | |
| P22626 | P22626 | Heterogeneous nucl | HNRNPA2B1 | 15970000 | 6 | Heterogeneo | 1 | | 5 |
| P08708 | P08708 | 40S ribosomal prote | RPS17 | 15968000 | 5 | 40S ribosom | 1 | | 3 |
| P17540 | P17540 | Creatine kinase S-ty | CKMT2 | 15558000 | 6 | Creatine kina | 1 | | 6 |
| P13639 | P13639 | Elongation factor 2 | EEF2 | 15370000 | 7 | Elongation fa | 1 | | 6 |
| Q14194 | Q14194 | Dihydropyrimidinase | CRMP1 | 15063000 | 9 | Dihydropyrim | 1 | | 9 |
| P46776 | P46776 | 60S ribosomal prote | RPL27A | 14947000 | 2 | 60S ribosom | 1 | | 2 |
| P10809 | P10809 | 60 kDa heat shock p | HSPD1 | 14304000 | 7 | 60 kDa heat | 1 | | 7 |
| P61978 | P61978 | Heterogeneous nucl | HNRNPK | 14193000 | 3 | Heterogeneo | 1 | | 3 |
| O43809 | O43809 | Cleavage and polyac | NUDT21 | 13967000 | 4 | Cleavage and | 1 | | 4 |
| P61247 | P61247 | 40S ribosomal prote | RPS3A | 13793000 | 4 | 40S ribosom | 1 | | 3 |
| P23396 | P23396 | 40S ribosomal prote | RPS3 | 13154000 | 5 | 40S ribosom | 1 | | 3 |
| P09972 | P09972 | Fructose-bisphospha | ALDOC | 12835000 | 6 | Fructose-bis | 1 | | 6 |
| P08865 | P08865 | 40S ribosomal prote | RPSA | 12816000 | 3 | 40S ribosom | 1 | | 3 |
| P62314 | P62314 | Small nuclear riboni | SNRPD1 | 12739000 | 1 | Small nuclea | 1 | | 1 |
| Q9NYI0 | Q9NYI0 | PH and SEC7 domain | PSD3 | 12652000 | 7 | PH and SEC7 | 1 | | 5 |
| P09104;P13929 | P09104 | Gamma-enolase | ENO2 | 11765000 | 6 | Gamma-eno | 2 | 6;1 | |
| P83731 | P83731 | 60S ribosomal prote | RPL24 | 11747000 | 3 | 60S ribosom | 1 | | 2 |
| Q92777 | Q92777 | Synapsin-2 | SYN2 | 11695000 | 5 | Synapsin-2 C | 1 | | 5 |
| P37840 | P37840 | Alpha-synuclein | SNCA | 11533000 | 4 | Alpha-synucl | 1 | | 2 |
| P06733 | P06733 | Alpha-enolase | ENO1 | 11466000 | 7 | Alpha-enolas | 1 | | 5 |
| Q14165 | Q14165 | Malectin | MLEC | 11370000 | 5 | Malectin OS= | 1 | | 4 |
| P07437;Q9BUF5;CON_ | P07437 | Tubulin beta chain | TUBB | 11305000 | 8 | Tubulin beta | 6 | 8;2;1;1;1;1 | |

| | | | | | | | | | |
|---|---|---|---|---|---|---|---|---|---|
| P21281;P15313 | P21281;P15313 | V-type proton ATPa: | ATP6V1B2;ATP( | 11245000 | 5 | V-type protoı | 2 | 4;3 | |
| P07384 | P07384 | Calpain-1 catalytic s | CAPN1 | 11228000 | 3 | Calpain-1 cat | 1 | | 3 |
| P60866 | P60866 | 40S ribosomal prote | RPS20 | 11209000 | 2 | 40S ribosom: | 1 | | 2 |
| O75323 | O75323 | Protein NipSnap hor | GBAS | 11188000 | 4 | Protein NipSı | 1 | | 2 |
| O43837 | O43837 | Isocitrate dehydroge | IDH3B | 10931000 | 4 | Isocitrate del | 1 | | 2 |
| P62841 | P62841 | 40S ribosomal prote | RPS15 | 10782000 | 1 | 40S ribosom: | 1 | | 1 |
| P61160 | P61160 | Actin-related protei | ACTR2 | 10648000 | 5 | Actin-relatec | 1 | | 5 |
| P61163 | P61163 | Alpha-centractin | ACTR1A | 10564000 | 3 | Alpha-centra | 1 | | 4 |
| Q13126 | Q13126 | S-methyl-5-thioadeı | MTAP | 10414000 | 2 | S-methyl-5-t | 1 | | 1 |
| P38646 | P38646 | Stress-70 protein, n | HSPA9 | 10384000 | 8 | Stress-70 prc | 1 | | 8 |
| P42025 | P42025 | Beta-centractin | ACTR1B | 10322000 | 6 | Beta-centrac | 1 | | 6 |
| P11940;Q13310;Q9H36 | P11940;Q13310;Q9H36 | Polyadenylate-bindi | PABPC1;PABPC | 10234000 | 7 | Polyadenylat | 5 | 5;4;3;2;1 | |
| P01617;P01614;P0630! | P01617;P01614;P0630! | Ig kappa chain V-II region TEW;Ig ka | | 10168000 | 2 | Ig kappa chai | 4 | 1;1;1;1 | |
| P62854;Q5JNZ5 | P62854;Q5JNZ5 | 40S ribosomal prote | RPS26;RPS26P1 | 10119000 | 2 | 40S ribosom: | 2 | 2;1 | |
| Q02413 | Q02413 | Desmoglein-1 | DSG1 | 10066000 | 12 | Desmoglein- | 1 | | 7 |
| P61224;A6NIZ1;P6283 | P61224;A6NIZ1;P6283 | Ras-related protein | RAP1B;RAP1A | 10017000 | 4 | Ras-related ı | 3 | 4;4;2 | |
| P32969 | P32969 | 60S ribosomal prote | RPL9 | 9846400 | 4 | 60S ribosom: | 1 | | 3 |
| Q9UBV8 | Q9UBV8 | Peflin | PEF1 | 9783500 | 3 | Peflin OS=Hc | 1 | | 3 |
| P63000;P60763;P1515: | P63000;P60763;P1515: | Ras-related C3 botu | RAC1;RAC3;RA( | 9725100 | 1 | Ras-related ( | 3 | 2;2;2 | |
| P78559 | P78559 | Microtubule-associa | MAP1A | 9527400 | 5 | Microtubule- | 1 | | 3 |
| O75077 | O75077 | Disintegrin and met | ADAM23 | 9506000 | 3 | Disintegrin a | 1 | | 3 |
| Q05639 | Q05639 | Elongation factor 1- | EEF1A2 | 9401600 | 4 | Elongation fa | 1 | | 6 |
| Q9UBS4 | Q9UBS4 | DnaJ homolog subfa | DNAJB11 | 9367600 | 3 | DnaJ homolo | 1 | | 3 |
| P62829 | P62829 | 60S ribosomal prote | RPL23 | 9353600 | 3 | 60S ribosom: | 1 | | 2 |
| P60953;P17081;P8409! | P60953;P17081;P8409! | Cell division control | CDC42;RHOQ;R | 9217100 | 3 | Cell division ( | 4 | 2;2;1;1 | |
| P62244 | P62244 | 40S ribosomal prote | RPS15A | 9216100 | 2 | 40S ribosom: | 1 | | 2 |
| P51991 | P51991 | Heterogeneous nucl | HNRNPA3 | 9212000 | 5 | Heterogeneo | 1 | | 4 |
| P04220;P01871 | P04220;P01871 | Ig mu heavy chain d | IGHM | 8880500 | 2 | Ig mu heavy | 2 | 2;2 | |
| P61513 | P61513 | 60S ribosomal prote | RPL37A | 8862400 | 2 | 60S ribosom: | 1 | | 1 |
| P59768 | P59768 | Guanine nucleotide- | GNG2 | 8760300 | 1 | Guanine nucl | 1 | | 1 |
| P26368 | P26368 | Splicing factor U2AI | U2AF2 | 8717200 | 4 | Splicing factc | 1 | | 3 |
| P50213 | P50213 | Isocitrate dehydroge | IDH3A | 8562800 | 3 | Isocitrate del | 1 | | 3 |
| P61981;Q04917 | P61981 | 14-3-3 protein gamı | YWHAG | 8467800 | 4 | 14-3-3 protei | 2 | 4;1 | |
| P67775 | P67775 | Serine/threonine-pr | PPP2CA | 8095600 | 6 | Serine/threo | 1 | | 6 |
| P62760 | P62760 | Visinin-like protein : | VSNL1 | 7923200 | 5 | Visinin-like p | 1 | | 4 |
| P60660;P14649 | P60660;P14649 | Myosin light polypeı | MYL6;MYL6B | 7911800 | 5 | Myosin light | 2 | 4;2 | |
| P23284 | P23284 | Peptidyl-prolyl cis-tr | PPIB | 7801900 | 4 | Peptidyl-prol | 1 | | 3 |
| P40925 | P40925 | Malate dehydrogen: | MDH1 | 7734600 | 3 | Malate dehy | 1 | | 2 |

| | | | | | | | | |
|---|---|---|---|---|---|---|---|---|
| P62888 | P62888 | 60S ribosomal prote | RPL30 | 7732700 | 4 | 60S ribosom | 1 | 4 |
| Q99873;Q9NR22 | Q99873 | Protein arginine N-r | PRMT1 | 7719100 | 4 | Protein argin | 2 | 4;1 |
| Q13151 | Q13151 | Heterogeneous nucl | HNRNPA0 | 7620000 | 2 | Heterogeneo | 1 | 2 |
| P23526 | P23526 | Adenosylhomocyste | AHCY | 7602000 | 4 | Adenosylhon | 1 | 4 |
| Q13642 | Q13642 | Four and a half LIM | FHL1 | 7482300 | 2 | Four and a h | 1 | 2 |
| Q7L014 | Q7L014 | Probable ATP-deper | DDX46 | 7424000 | 6 | Probable ATI | 1 | 5 |
| Q9UJZ1 | Q9UJZ1 | Stomatin-like protei | STOML2 | 7420600 | 6 | Stomatin-like | 1 | 6 |
| P62899 | P62899 | 60S ribosomal prote | RPL31 | 7411500 | 2 | 60S ribosom | 1 | 2 |
| O60506 | O60506 | Heterogeneous nucl | SYNCRIP | 7314200 | 1 | Heterogeneo | 1 | 1 |
| P62847 | P62847 | 40S ribosomal prote | RPS24 | 7312400 | 3 | 40S ribosom | 1 | 2 |
| P46821 | P46821 | Microtubule-associa | MAP1B | 7300800 | 2 | Microtubule- | 1 | 2 |
| P53999 | P53999 | Activated RNA polyr | SUB1 | 7292800 | 1 | Activated RN | 1 | 1 |
| P00352 | P00352 | Retinal dehydrogena | ALDH1A1 | 7249800 | 2 | Retinal dehy | 1 | 2 |
| P31943 | P31943 | Heterogeneous nucl | HNRNPH1 | 7096000 | 4 | Heterogeneo | 1 | 4 |
| P14923 | P14923 | Junction plakoglobir | JUP | 7045400 | 6 | Junction plak | 1 | 6 |
| O43865;Q96HN2 | O43865 | Putative adenosylho | AHCYL1 | 6986800 | 3 | Adenosylhon | 2 | 3;1 |
| Q16851 | Q16851 | UTP--glucose-1-pho | UGP2 | 6971600 | 2 | UTP--glucose | 1 | 3 |
| P61254;Q9UNX3 | P61254;Q9UNX3 | 60S ribosomal prote | RPL26;RPL26L1 | 6936900 | 2 | 60S ribosom | 2 | 2;1 |
| P49821 | P49821 | NADH dehydrogenas | NDUFV1 | 6902900 | 5 | NADH dehydi | 1 | 5 |
| Q9ULR3 | Q9ULR3 | Protein phosphatase | PPM1H | 6799500 | 6 | Protein phos | 1 | 4 |
| P29401 | P29401 | Transketolase | TKT | 6577300 | 2 | Transketolas | 1 | 1 |
| O60869 | O60869 | Endothelial differen | EDF1 | 6550100 | 3 | Endothelial c | 1 | 3 |
| P47756 | P47756 | F-actin-capping prot | CAPZB | 6542000 | 6 | F-actin-cappi | 1 | 4 |
| Q9BY11 | Q9BY11 | Protein kinase C anc | PACSIN1 | 6406200 | 4 | Protein kinas | 1 | 3 |
| P56385 | P56385 | ATP synthase subun | ATP5I | 6382100 | 1 | ATP synthase | 1 | 1 |
| P28331 | P28331 | NADH-ubiquinone o: | NDUFS1 | 6375200 | 4 | NADH-ubiqui | 1 | 4 |
| P62995 | P62995 | Transformer-2 prote | TRA2B | 6202700 | 2 | Transformer- | 1 | 2 |
| Q92747 | Q92747 | Actin-related protei | ARPC1A | 6172400 | 4 | Actin-relatec | 1 | 3 |
| P09471;P11488;P6309( | P09471 | Guanine nucleotide- | GNAO1 | 6090500 | 3 | Guanine nucl | 9 | 3;1;1;1;1;1;1; |
| P00338;Q6ZMR3;P078( | P00338;Q6ZMR3;P078( | L-lactate dehydroge | LDHA;LDHAL6A; | 6064800 | 1 | L-lactate deh | 3 | 2;1;1 |
| O75390 | O75390 | Citrate synthase, mi | CS | 6029900 | 2 | Citrate synth | 1 | 2 |
| Q9BPX5 | Q9BPX5 | Actin-related protei | ARPC5L | 5992700 | 3 | Actin-relatec | 1 | 2 |
| Q9NRW1;P20340;Q14( | Q9NRW1;P20340;Q14( | Ras-related protein | RAB6B;RAB6A; | 5866600 | 1 | Ras-related p | 9 | 1;1;1;1;1;1;1; |
| P18621 | P18621 | 60S ribosomal prote | RPL17 | 5760700 | 1 | 60S ribosom | 1 | 1 |
| P62826 | P62826 | GTP-binding nuclear | RAN | 5755500 | 2 | GTP-binding | 1 | 2 |
| P27348 | P27348 | 14-3-3 protein theta | YWHAQ | 5738900 | 2 | 14-3-3 protei | 1 | 2 |
| P62424 | P62424 | 60S ribosomal prote | RPL7A | 5715400 | 3 | 60S ribosom | 1 | 3 |
| Q92598 | Q92598 | Heat shock protein 1 | HSPH1 | 5622100 | 8 | Heat shock p | 1 | 6 |

| | | | | | | | | | |
|---|---|---|---|---|---|---|---|---|---|
| Q5T0D9 | Q5T0D9 | Tumor protein p63-r | TPRG1L | 5598200 | 2 | Tumor protei | 1 | | 2 |
| Q13247 | Q13247 | Serine/arginine-rich | SRSF6 | 5556000 | 3 | Serine/argini | 1 | | 3 |
| P52907 | P52907 | F-actin-capping prot | CAPZA1 | 5479800 | 4 | F-actin-cappi | 1 | | 5 |
| Q16181 | Q16181 | Septin-7 | 7-Sep | 5475600 | 5 | Septin-7 OS= | 1 | | 4 |
| P13804 | P13804 | Electron transfer fla | ETFA | 5388700 | 3 | Electron tran | 1 | | 3 |
| P29218 | P29218 | Inositol monophospl | IMPA1 | 5362100 | 1 | Inositol mon | 1 | | 1 |
| P00558 | P00558 | Phosphoglycerate ki | PGK1 | 5359900 | 3 | Phosphoglyce | 1 | | 2 |
| Q16629 | Q16629 | Serine/arginine-rich | SRSF7 | 5315700 | 3 | Serine/argini | 1 | | 1 |
| A6NDG6 | A6NDG6 | Phosphoglycolate pl | PGP | 5286600 | 2 | Glycerol-3-pl | 1 | | 2 |
| P31937 | P31937 | 3-hydroxyisobutyrat | HIBADH | 5211400 | 4 | 3-hydroxyisol | 1 | | 3 |
| Q15084 | Q15084 | Protein disulfide-iso | PDIA6 | 5171300 | 3 | Protein disul | 1 | | 3 |
| P62304 | P62304 | Small nuclear ribonu | SNRPE | 5167000 | 1 | Small nuclea | 1 | | 1 |
| P47813;O14602 | P47813;O14602 | Eukaryotic translatic | EIF1AX;EIF1AY | 5078000 | 2 | Eukaryotic tr | 2 | 2;2 | |
| Q14103 | Q14103 | Heterogeneous nucl | HNRNPD | 4997300 | 3 | Heterogeneo | 1 | | 3 |
| P60981 | P60981 | Destrin | DSTN | 4984000 | 0 | Destrin OS=H | 1 | | 1 |
| P59998 | P59998 | Actin-related protei | ARPC4 | 4939500 | 2 | Actin-relatec | 1 | | 2 |
| Q969T9 | Q969T9 | WW domain-binding | WBP2 | 4924600 | 1 | WW domain | 1 | | 1 |
| P62318 | P62318 | Small nuclear ribonu | SNRPD3 | 4882000 | 3 | Small nuclea | 1 | | 2 |
| Q9BPW8 | Q9BPW8 | Protein NipSnap hor | NIPSNAP1 | 4855700 | 2 | Protein NipS | 1 | | 2 |
| P62258 | P62258 | 14-3-3 protein epsil | YWHAE | 4731900 | 3 | 14-3-3 protei | 1 | | 3 |
| P05198 | P05198 | Eukaryotic translatic | EIF2S1 | 4673700 | 5 | Eukaryotic tr | 1 | | 4 |
| P20839 | P20839 | Inosine-5-monophos | IMPDH1 | 4667100 | 1 | Inosine-5-mo | 1 | | 1 |
| P63167 | P63167 | Dynein light chain 1, | DYNLL1 | 4652300 | 4 | Dynein light | 1 | | 1 |
| P15880 | P15880 | 40S ribosomal prote | RPS2 | 4618300 | 3 | 40S ribosoma | 1 | | 2 |
| P62633 | P62633 | Cellular nucleic acid | CNBP | 4579200 | 2 | Cellular nucle | 1 | | 2 |
| P53597 | P53597 | Succinyl-CoA ligase | SUCLG1 | 4445500 | 1 | Succinyl-CoA | 1 | | 1 |
| P62937 | P62937 | Peptidyl-prolyl cis-tr | PPIA | 4432600 | 1 | Peptidyl-prol | 1 | | 1 |
| O15143 | O15143 | Actin-related protei | ARPC1B | 4423800 | 1 | Actin-relatec | 1 | | 1 |
| Q8TEA8 | Q8TEA8 | D-tyrosyl-tRNA(Tyr) | DTD1 | 4409600 | 2 | D-tyrosyl-tRN | 1 | | 2 |
| O95670 | O95670 | V-type proton ATPas | ATP6V1G2 | 4406600 | 3 | V-type proto | 1 | | 2 |
| Q9UN36 | Q9UN36 | Protein NDRG2 | NDRG2 | 4400400 | 3 | Protein NDR( | 1 | | 2 |
| P62917 | P62917 | 60S ribosomal prote | RPL8 | 4374300 | 2 | 60S ribosoma | 1 | | 2 |
| P46781 | P46781 | 40S ribosomal prote | RPS9 | 4374200 | 3 | 40S ribosoma | 1 | | 3 |
| Q8IXJ6 | Q8IXJ6 | NAD-dependent pro | SIRT2 | 4354900 | 1 | NAD-depend | 1 | | 1 |
| O75489 | O75489 | NADH dehydrogenas | NDUFS3 | 4346900 | 2 | NADH dehydr | 1 | | 2 |
| P46783;Q9NQ39 | P46783;Q9NQ39 | 40S ribosomal prote | RPS10;RPS10P5 | 4341300 | 1 | 40S ribosoma | 2 | 1;1 | |
| P51649 | P51649 | Succinate-semialdel | ALDH5A1 | 4291200 | 2 | Succinate-se | 1 | | 1 |
| P49419 | P49419 | Alpha-aminoadipic s | ALDH7A1 | 4227300 | 3 | Alpha-amino | 1 | | 3 |

| | | | | | | | | |
|---|---|---|---|---|---|---|---|---|
| Q9UHX1 | Q9UHX1 | Poly(U)-binding-spli | PUF60 | 4188200 | 8 | Poly(U)-bindi | 1 | 5 |
| P62280 | P62280 | 40S ribosomal prote | RPS11 | 4165800 | 3 | 40S ribosoma | 1 | 2 |
| O15511 | O15511 | Actin-related protei | ARPC5 | 4154200 | 2 | Actin-relatec | 1 | 2 |
| P38117 | P38117 | Electron transfer fla | ETFB | 4138900 | 1 | Electron tran | 1 | 1 |
| Q13363 | Q13363 | C-terminal-binding | CTBP1 | 4115500 | 2 | C-terminal-b | 1 | 2 |
| P21912 | P21912 | Succinate dehydrog | SDHB | 4088700 | 1 | Succinate de | 1 | 2 |
| P61586;P08134 | P61586;P08134 | Transforming protei | RHOA;RHOC | 4070500 | 3 | Transforming | 2 | 3;3 |
| P28330 | P28330 | Long-chain specific | ACADL | 4031600 | 1 | Long-chain s | 1 | 1 |
| P55072 | P55072 | Transitional endopla | VCP | 4009000 | 4 | Transitional | 1 | 3 |
| Q6IS14;P63241;Q9GZV | Q6IS14;P63241;Q9GZV | Eukaryotic translatic | EIF5AL1;EIF5A; | 3979000 | 3 | Eukaryotic tr | 3 | 2;2;1 |
| P19338 | P19338 | Nucleolin | NCL | 3954700 | 2 | Nucleolin OS | 1 | 2 |
| O14775 | O14775 | Guanine nucleotide- | GNB5 | 3877700 | 1 | Guanine nucl | 1 | 1 |
| P52565 | P52565 | Rho GDP-dissociatic | ARHGDIA | 3876000 | 2 | Rho GDP-dis | 1 | 2 |
| P24666 | P24666 | Low molecular weig | ACP1 | 3820900 | 3 | Low molecul | 1 | 3 |
| P14625 | P14625 | Endoplasmin | HSP90B1 | 3818200 | 1 | Endoplasmin | 1 | 1 |
| P51149 | P51149 | Ras-related protein | RAB7A | 3804700 | 5 | Ras-related | 1 | 5 |
| P12532 | P12532 | Creatine kinase U-ty | CKMT1A | 3764000 | 1 | Creatine kina | 1 | 3 |
| P14927 | P14927 | Cytochrome b-c1 co | UQCRB | 3714200 | 1 | Cytochrome | 1 | 1 |
| Q99880;Q99879;Q998 | Q99880;Q99879;Q998 | Histone H2B type 1- | HIST1H2BL;HIS | 3669100 | 2 | Histone H2B | 14 | 2;2;2;2;2;2;2; |
| Q92930 | Q92930 | Ras-related protein | RAB8B | 3646700 | 1 | Ras-related | 1 | 1 |
| P63215 | P63215 | Guanine nucleotide- | GNG3 | 3645500 | 2 | Guanine nucl | 1 | 1 |
| Q99962 | Q99962 | Endophilin-A1 | SH3GL2 | 3639400 | 4 | Endophilin-A | 1 | 3 |
| P19105;O14950;P2484 | P19105;O14950 | Myosin regulatory li | MYL12A;MYL12 | 3623000 | 3 | Myosin regul | 3 | 3;3;1 |
| Q15717 | Q15717 | ELAV-like protein 1 | ELAVL1 | 3621500 | 2 | ELAV-like pro | 1 | 2 |
| P62266 | P62266 | 40S ribosomal prote | RPS23 | 3616500 | 1 | 40S ribosoma | 1 | 1 |
| Q14498 | Q14498 | RNA-binding protein | RBM39 | 3604000 | 5 | RNA-binding | 1 | 3 |
| Q9UPV7 | Q9UPV7 | Protein KIAA1045 | KIAA1045 | 3599400 | 1 | PHD finger pi | 1 | 1 |
| P61019 | P61019 | Ras-related protein | RAB2A | 3589000 | 2 | Ras-related | 1 | 2 |
| P54132 | P54132 | Bloom syndrome pr | BLM | 3586200 | 1 | Bloom syndr | 1 | 1 |
| Q96P63 | Q96P63 | Serpin B12 | SERPINB12 | 3585400 | 4 | Serpin B12 C | 1 | 4 |
| Q13303 | Q13303 | Voltage-gated pota | KCNAB2 | 3519400 | 2 | Voltage-gate | 1 | 2 |
| P04259 | P04259 | Keratin, type II cytos | KRT6B | 3498500 | 3 | Keratin, type | 1 | 12 |
| Q8NC51 | Q8NC51 | Plasminogen activat | SERBP1 | 3478600 | 4 | Plasminogen | 1 | 3 |
| Q6PUV4 | Q6PUV4 | Complexin-2 | CPLX2 | 3452100 | 2 | Complexin-2 | 1 | 1 |
| O15083 | O15083 | ERC protein 2 | ERC2 | 3417500 | 1 | ERC protein | 1 | 1 |
| P62750 | P62750 | 60S ribosomal prote | RPL23A | 3415300 | 1 | 60S ribosoma | 1 | 1 |
| P28838 | P28838 | Cytosol aminopeptic | LAP3 | 3402100 | 2 | Cytosol amin | 1 | 2 |
| O95970 | O95970 | Leucine-rich glioma- | LGI1 | 3400400 | 3 | Leucine-rich | 1 | 2 |

| | | | | | | | | | |
|---|---|---|---|---|---|---|---|---|---|
| P14174 | P14174 | Macrophage migrat | MIF | | 3368100 | 1 | Macrophage | 1 | 1 |
| P49207 | P49207 | 60S ribosomal prote | RPL34 | | 3323300 | 1 | 60S ribosom | 1 | 1 |
| O15372 | O15372 | Eukaryotic translatic | EIF3H | | 3275400 | 1 | Eukaryotic tr | 1 | 1 |
| P51553 | P51553 | Isocitrate dehydrog | IDH3G | | 3218600 | 1 | Isocitrate de | 1 | 1 |
| P84098 | P84098 | 60S ribosomal prote | RPL19 | | 3172700 | 1 | 60S ribosom | 1 | 1 |
| P63208 | P63208 | S-phase kinase-assc | SKP1 | | 3142200 | 1 | S-phase kina | 1 | 1 |
| O15075 | O15075 | Serine/threonine-pr | DCLK1 | | 3120100 | 2 | Serine/threo | 1 | 3 |
| P62913 | P62913 | 60S ribosomal prote | RPL11 | | 3116400 | 3 | 60S ribosom | 1 | 2 |
| Q01469 | Q01469 | Fatty acid-binding p | FABP5 | | 3113400 | 2 | Fatty acid-bi | 1 | 2 |
| P20336 | P20336 | Ras-related protein | RAB3A | | 3085200 | 2 | Ras-related | 1 | 2 |
| P31689 | P31689 | DnaJ homolog subfa | DNAJA1 | | 2973000 | 4 | DnaJ homolo | 1 | 3 |
| P35637;Q92804 | P35637;Q92804 | RNA-binding proteir | FUS;TAF15 | | 2925900 | 1 | RNA-binding | 2 | 1;1 |
| O60861 | O60861 | Growth arrest-speci | GAS7 | | 2894700 | 1 | Growth arres | 1 | 1 |
| Q9Y333 | Q9Y333 | U6 snRNA-associate | LSM2 | | 2893300 | 2 | U6 snRNA-as | 1 | 1 |
| P19404 | P19404 | NADH dehydrogena | NDUFV2 | | 2864400 | 4 | NADH dehyd | 1 | 3 |
| P30041 | P30041 | Peroxiredoxin-6 | PRDX6 | | 2811600 | 1 | Peroxiredoxi | 1 | 1 |
| P12268 | P12268 | Inosine-5-monopho | IMPDH2 | | 2793800 | 2 | Inosine-5-mc | 1 | 2 |
| P06748 | P06748 | Nucleophosmin | NPM1 | | 2784600 | 3 | Nucleophosn | 1 | 2 |
| O95232 | O95232 | Luc7-like protein 3 | LUC7L3 | | 2764400 | 4 | Luc7-like pro | 1 | 3 |
| P62166 | P62166 | Neuronal calcium se | NCS1 | | 2733100 | 2 | Neuronal cal | 1 | 2 |
| P07339 | P07339 | Cathepsin D;Catheps | CTSD | | 2679300 | 2 | Cathepsin D | 1 | 2 |
| P67809 | P67809 | Nuclease-sensitive | YBX1 | | 2660900 | 3 | Nuclease-se | 1 | 3 |
| Q9P2R7 | Q9P2R7 | Succinyl-CoA ligase | SUCLA2 | | 2639500 | 3 | Succinyl-CoA | 1 | 3 |
| Q13595 | Q13595 | Transformer-2 prote | TRA2A | | 2622600 | 1 | Transformer | 1 | 1 |
| P09661 | P09661 | U2 small nuclear rit | SNRPA1 | | 2619800 | 2 | U2 small nuc | 1 | 2 |
| Q13228 | Q13228 | Selenium-binding pı | SELENBP1 | | 2582700 | 1 | Selenium-bir | 1 | 1 |
| Q8WUM4 | Q8WUM4 | Programmed cell de | PDCD6IP | | 2580400 | 3 | Programmec | 1 | 2 |
| P98179 | P98179 | RNA-binding proteir | RBM3 | | 2569000 | 2 | RNA-binding | 1 | 2 |
| P39023 | P39023 | 60S ribosomal prote | RPL3 | | 2544400 | 3 | 60S ribosom | 1 | 3 |
| O15144 | O15144 | Actin-related protei | ARPC2 | | 2498400 | 2 | Actin-relatec | 1 | 2 |
| P25789 | P25789 | Proteasome subunit | PSMA4 | | 2470600 | 2 | Proteasome | 1 | 2 |
| Q15019 | Q15019 | Septin-2 | | 2-Sep | 2458600 | 2 | Septin-2 OS= | 1 | 2 |
| Q6NUI6 | Q6NUI6 | Chondroadherin-like | CHADL | | 2409000 | 1 | Chondroadhe | 1 | 1 |
| P62979;P62987;P0CG4 | P62979;P62987;P0CG4 | Ubiquitin-40S ribos | RPS27A;UBA52 | | 2404800 | 2 | Ubiquitin-40 | 4 | 2;1;1;1 |
| Q9UJW0 | Q9UJW0 | Dynactin subunit 4 | DCTN4 | | 2394400 | 3 | Dynactin sub | 1 | 3 |
| Q9UI09 | Q9UI09 | NADH dehydrogena | NDUFA12 | | 2374300 | 2 | NADH dehyd | 1 | 1 |
| P78371 | P78371 | T-complex protein 1 | CCT2 | | 2342000 | 1 | T-complex pı | 1 | 1 |
| O00499 | O00499 | Myc box-dependent | BIN1 | | 2325800 | 4 | Myc box-dep | 1 | 3 |

| | | | | | | | | | |
|---|---|---|---|---|---|---|---|---|---|
| O60884 | O60884 | DnaJ homolog subfa | DNAJA2 | 2318700 | 2 | DnaJ homolo | 1 | | 1 |
| P16298;P48454 | P16298 | Serine/threonine-pr | PPP3CB | 2315000 | 2 | Serine/threo | 2 | 6;1 | |
| Q9NR46 | Q9NR46 | Endophilin-B2 | SH3GLB2 | 2309200 | 2 | Endophilin-B | 1 | | 2 |
| Q13835 | Q13835 | Plakophilin-1 | PKP1 | 2295400 | 3 | Plakophilin-1 | 1 | | 3 |
| P62308;A8MWD9 | P62308;A8MWD9 | Small nuclear riboni | SNRPG;SNRPG | 2272500 | 2 | Small nuclea | 2 | 2;2 | |
| P12429 | P12429 | Annexin A3 | ANXA3 | 2209600 | 1 | Annexin A3 ( | 1 | | 1 |
| Q14011 | Q14011 | Cold-inducible RNA- | CIRBP | 2191100 | 3 | Cold-inducibl | 1 | | 2 |
| Q6NVV1;P40429 | Q6NVV1;P40429 | Putative 60S ribosor | RPL13AP3;RPL1 | 2174600 | 1 | Putative 60S | 2 | 1;1 | |
| P31150;P50395 | P31150;P50395 | Rab GDP dissociatio | GDI1;GDI2 | 2173600 | 3 | Rab GDP diss | 2 | 3;2 | |
| P62277 | P62277 | 40S ribosomal prote | RPS13 | 2150100 | 2 | 40S ribosom | 1 | | 2 |
| Q92561 | Q92561 | Phytanoyl-CoA hydro | PHYHIP | 2121400 | 2 | Phytanoyl-Co | 1 | | 2 |
| P07305 | P07305 | Histone H1.0;Histon | H1F0 | 2119600 | 2 | Histone H1.0 | 1 | | 2 |
| P26583;B2RPK0;P0942 | P26583;B2RPK0;P0942 | High mobility group | HMGB2;HMGB | 2118500 | 2 | High mobility | 3 | 1;1;1 | |
| Q9GZT4 | Q9GZT4 | Serine racemase | SRR | 2100100 | 1 | Serine racem | 1 | | 1 |
| P49411 | P49411 | Elongation factor Tu | TUFM | 2099900 | 1 | Elongation fa | 1 | | 1 |
| P26641 | P26641 | Elongation factor 1- | EEF1G | 2098000 | 2 | Elongation fa | 1 | | 2 |
| Q9BUL8 | Q9BUL8 | Programmed cell de | PDCD10 | 2093800 | 2 | Programmed | 1 | | 2 |
| P04179 | P04179 | Superoxide dismuta | SOD2 | 2085000 | 1 | Superoxide d | 1 | | 1 |
| P51452 | P51452 | Dual specificity prot | DUSP3 | 2077100 | 2 | Dual specific | 1 | | 1 |
| P07237 | P07237 | Protein disulfide-iso | P4HB | 2074900 | 3 | Protein disul | 1 | | 2 |
| Q92945 | Q92945 | Far upstream eleme | KHSRP | 1969600 | 3 | Far upstream | 1 | | 3 |
| O75078 | O75078 | Disintegrin and met | ADAM11 | 1957200 | 2 | Disintegrin a | 1 | | 1 |
| Q4G0N4 | Q4G0N4 | NAD kinase 2, mitoc | NADK2 | 1945800 | 2 | NAD kinase 2 | 1 | | 2 |
| Q9Y266 | Q9Y266 | Nuclear migration p | NUDC | 1938300 | 2 | Nuclear migr | 1 | | 2 |
| P25786 | P25786 | Proteasome subunit | PSMA1 | 1938100 | 1 | Proteasome | 1 | | 1 |
| Q13765;E9PAV3 | Q13765;E9PAV3 | Nascent polypeptide | NACA | 1922500 | 2 | Nascent poly | 2 | 2;2 | |
| O60262 | O60262 | Guanine nucleotide- | GNG7 | 1914000 | 1 | Guanine nucl | 1 | | 1 |
| Q9Y3E1 | Q9Y3E1 | Hepatoma-derived g | HDGFRP3 | 1898500 | 1 | Hepatoma-d | 1 | | 1 |
| O14531 | O14531 | Dihydropyrimidinase | DPYSL4 | 1881200 | 2 | Dihydropyrim | 1 | | 2 |
| O43181 | O43181 | NADH dehydrogenas | NDUFS4 | 1878800 | 2 | NADH dehydi | 1 | | 1 |
| Q9Y371 | Q9Y371 | Endophilin-B1 | SH3GLB1 | 1858000 | 2 | Endophilin-B | 1 | | 2 |
| Q9Y295 | Q9Y295 | Developmentally-re | DRG1 | 1854200 | 1 | Developmen | 1 | | 3 |
| Q13825 | Q13825 | Methylglutaconyl-Co | AUH | 1851900 | 1 | Methylglutac | 1 | | 1 |
| P36871 | P36871 | Phosphoglucomutas | PGM1 | 1850300 | 1 | Phosphogluc | 1 | | 1 |
| P50990 | P50990 | T-complex protein 1 | CCT8 | 1846600 | 3 | T-complex pr | 1 | | 3 |
| P14314 | P14314 | Glucosidase 2 subur | PRKCSH | 1818000 | 1 | Glucosidase | 1 | | 1 |
| P18669;P15259;Q8N0Y | P18669 | Phosphoglycerate m | PGAM1 | 1806800 | 5 | Phosphoglyce | 3 | 3;1;1 | |
| P68371;P04350;Q1350 | P68371;P04350;Q1350 | Tubulin beta-4B cha | TUBB4B;TUBB4 | 1797200 | 1 | Tubulin beta- | 3 | 7;6;6 | |

| | | | | | | | | |
|---|---|---|---|---|---|---|---|---|
| Q15067 | Q15067 | Peroxisomal acyl-co | ACOX1 | 1793900 | 2 | Peroxisomal | 1 | 2 |
| P14866 | P14866 | Heterogeneous nucl | HNRNPL | 1775500 | 2 | Heterogeneo | 1 | 2 |
| O43175 | O43175 | D-3-phosphoglycera | PHGDH | 1768900 | 2 | D-3-phospho | 1 | 2 |
| Q9Y3U8 | Q9Y3U8 | 60S ribosomal prote | RPL36 | 1763700 | 1 | 60S ribosoma | 1 | 1 |
| P60903 | P60903 | Protein S100-A10 | S100A10 | 1701400 | 2 | Protein S100 | 1 | 2 |
| P63151 | P63151 | Serine/threonine-pr | PPP2R2A | 1700100 | 3 | Serine/threo | 1 | 2 |
| P61006 | P61006 | Ras-related protein | RAB8A | 1665500 | 3 | Ras-related p | 1 | 2 |
| Q01085 | Q01085 | Nucleolysin TIAR | TIAL1 | 1663000 | 2 | Nucleolysin T | 1 | 2 |
| Q92841 | Q92841 | Probable ATP-deper | DDX17 | 1660600 | 3 | Probable ATP | 1 | 3 |
| P49591 | P49591 | Serine--tRNA ligase | SARS | 1658000 | 1 | Serine--tRNA | 1 | 1 |
| P52209 | P52209 | 6-phosphogluconate | PGD | 1654900 | 2 | 6-phosphoglu | 1 | 2 |
| O43390 | O43390 | Heterogeneous nucl | HNRNPR | 1646000 | 2 | Heterogeneo | 1 | 2 |
| Q96DH6 | Q96DH6 | RNA-binding proteir | MSI2 | 1645700 | 2 | RNA-binding | 1 | 2 |
| P41091;Q2VIR3 | P41091;Q2VIR3 | Eukaryotic translatic | EIF2S3;EIF2S3L | 1637500 | 3 | Eukaryotic tra | 2 | 2;1 |
| Q15417 | Q15417 | Calponin-3 | CNN3 | 1622700 | 2 | Calponin-3 O | 1 | 2 |
| P48426 | P48426 | Phosphatidylinositol | PIP4K2A | 1622100 | 3 | Phosphatidyl | 1 | 2 |
| P21796 | P21796 | Voltage-dependent | VDAC1 | 1609700 | 3 | Voltage-depe | 1 | 2 |
| P55036;A2A3N6 | P55036;A2A3N6 | 26S proteasome noi | PSMD4;PIPSL | 1603700 | 2 | 26S proteaso | 2 | 2;2 |
| O94811 | O94811 | Tubulin polymerizat | TPPP | 1595500 | 1 | Tubulin polyr | 1 | 1 |
| Q16527 | Q16527 | Cysteine and glycine | CSRP2 | 1591600 | 2 | Cysteine and | 1 | 2 |
| P02751 | P02751 | Fibronectin;Anastell | FN1 | 1575000 | 4 | Fibronectin C | 1 | 3 |
| O75348 | O75348 | V-type proton ATPas | ATP6V1G1 | 1536600 | 1 | V-type proton | 1 | 1 |
| P37802 | P37802 | Transgelin-2 | TAGLN2 | 1534100 | 1 | Transgelin-2 | 1 | 1 |
| Q08554 | Q08554 | Desmocollin-1 | DSC1 | 1512000 | 2 | Desmocollin- | 1 | 2 |
| Q5T749 | Q5T749 | Keratinocyte proline | KPRP | 1494300 | 2 | Keratinocyte | 1 | 2 |
| P01764;P01777;P01766 | P01764;P01777;P01766 | Ig heavy chain V-III r | IGHV3-23 | 1472400 | 1 | Ig heavy chai | 3 | 1;1;1 |
| P25787 | P25787 | Proteasome subunit | PSMA2 | 1464000 | 3 | Proteasome | 1 | 2 |
| Q9BTE1 | Q9BTE1 | Dynactin subunit 5 | DCTN5 | 1452100 | 1 | Dynactin sub | 1 | 1 |
| P28072 | P28072 | Proteasome subunit | PSMB6 | 1450800 | 1 | Proteasome | 1 | 1 |
| Q99747 | Q99747 | Gamma-soluble NSI | NAPG | 1445700 | 2 | Gamma-solu | 1 | 1 |
| Q63HR2;Q68CZ2;Q9HB | Q63HR2;Q68CZ2;Q9HB | Tensin-2;Tensin-3;T | TNS2;TNS3;TNS | 1442000 | 1 | Tensin-2 OS= | 3 | 1;1;1 |
| P13637;P50993;P0502 | P13637;P50993;P0502 | Sodium/potassium- | ATP1A3;ATP1A | 1409500 | 1 | Sodium/pota | 4 | 1;1;1;1 |
| P43304 | P43304 | Glycerol-3-phosphat | GPD2 | 1398100 | 2 | Glycerol-3-ph | 1 | 2 |
| P40227 | P40227 | T-complex protein 1 | CCT6A | 1390700 | 2 | T-complex pr | 1 | 2 |
| P23786 | P23786 | Carnitine O-palmito | CPT2 | 1363800 | 1 | Carnitine O-p | 1 | 1 |
| Q02218;Q9ULD0 | Q02218;Q9ULD0 | 2-oxoglutarate dehy | OGDH;OGDHL | 1363300 | 3 | 2-oxoglutara | 2 | 3;2 |
| Q9Y3Y2 | Q9Y3Y2 | Chromatin target of | CHTOP | 1361500 | 1 | Chromatin ta | 1 | 1 |
| P62081 | P62081 | 40S ribosomal prote | RPS7 | 1361400 | 1 | 40S ribosoma | 1 | 1 |

| | | | | | | | | |
|---|---|---|---|---|---|---|---|---|
| P63173 | P63173 | 60S ribosomal prote | RPL38 | 1360900 | 2 | 60S ribosom | 1 | 2 |
| O75131 | O75131 | Copine-3 | CPNE3 | 1358300 | 3 | Copine-3 OS= | 1 | 2 |
| P61020;P20339;P5114ε | P61020;P20339;P5114ε | Ras-related protein | RAB5B;RAB5A; | 1346400 | 2 | Ras-related p | 3 | 2;1;1 |
| P62910 | P62910 | 60S ribosomal prote | RPL32 | 1336100 | 1 | 60S ribosom | 1 | 1 |
| Q02252 | Q02252 | Methylmalonate-se | ALDH6A1 | 1329800 | 1 | Methylmalor | 1 | 1 |
| P31946 | P31946 | 14-3-3 protein beta/ | YWHAB | 1324700 | 2 | 14-3-3 protei | 1 | 3 |
| P11766 | P11766 | Alcohol dehydrogen | ADH5 | 1295300 | 2 | Alcohol dehy | 1 | 2 |
| P18065 | P18065 | Insulin-like growth f | IGFBP2 | 1290800 | 1 | Insulin-like g | 1 | 1 |
| P49189 | P49189 | 4-trimethylaminobu | ALDH9A1 | 1286500 | 2 | 4-trimethyla | 1 | 2 |
| P41223 | P41223 | Protein BUD31 hom | BUD31 | 1275500 | 1 | Protein BUD3 | 1 | 1 |
| P09496 | P09496 | Clathrin light chain / | CLTA | 1249200 | 2 | Clathrin light | 1 | 2 |
| P62136 | P62136 | Serine/threonine-pr | PPP1CA | 1237100 | 3 | Serine/threo | 1 | 2 |
| Q9NVJ2 | Q9NVJ2 | ADP-ribosylation fac | ARL8B | 1233300 | 1 | ADP-ribosyla | 1 | 1 |
| P31944 | P31944 | Caspase-14;Caspase | CASP14 | 1230900 | 1 | Caspase-14 ( | 1 | 2 |
| P31949 | P31949 | Protein S100-A11;Pr | S100A11 | 1229800 | 1 | Protein S100 | 1 | 1 |
| Q9BVA1;Q13885 | Q9BVA1;Q13885 | Tubulin beta-2B cha | TUBB2B;TUBB2 | 1229800 | 1 | Tubulin beta- | 2 | 6;6 |
| O60547 | O60547 | GDP-mannose 4,6 d | GMDS | 1212600 | 1 | GDP-mannos | 1 | 1 |
| Q96FJ2 | Q96FJ2 | Dynein light chain 2, | DYNLL2 | 1212200 | 0 | Dynein light ( | 1 | 1 |
| P09211 | P09211 | Glutathione S-trans | GSTP1 | 1204500 | 3 | Glutathione S | 1 | 3 |
| P10768 | P10768 | S-formylglutathione | ESD | 1194600 | 1 | S-formylgluta | 1 | 1 |
| P84090 | P84090 | Enhancer of rudime | ERH | 1193600 | 1 | Enhancer of r | 1 | 1 |
| P63244 | P63244 | Guanine nucleotide- | GNB2L1 | 1178100 | 2 | Receptor of a | 1 | 2 |
| P26196 | P26196 | Probable ATP-deper | DDX6 | 1175700 | 2 | Probable ATF | 1 | 2 |
| P36406 | P36406 | E3 ubiquitin-protein | TRIM23 | 1168800 | 1 | E3 ubiquitin- | 1 | 1 |
| P49006 | P49006 | MARCKS-related prc | MARCKSL1 | 1141700 | 1 | MARCKS-rela | 1 | 1 |
| P18085 | P18085 | ADP-ribosylation fac | ARF4 | 1122700 | 1 | ADP-ribosyla | 1 | 2 |
| P10515 | P10515 | Dihydrolipoyllysine-r | DLAT | 1078000 | 2 | Dihydrolipoyl | 1 | 2 |
| P29692 | P29692 | Elongation factor 1- | EEF1D | 1056200 | 1 | Elongation fa | 1 | 1 |
| Q14558 | Q14558 | Phosphoribosyl pyro | PRPSAP1 | 1049700 | 1 | Phosphoribos | 1 | 1 |
| Q8WVD5 | Q8WVD5 | RING finger protein | RNF141 | 1049100 | 1 | RING finger p | 1 | 1 |
| P34897 | P34897 | Serine hydroxymeth | SHMT2 | 1033600 | 1 | Serine hydro: | 1 | 1 |
| P25788 | P25788 | Proteasome subunit | PSMA3 | 1030900 | 1 | Proteasome | 1 | 1 |
| P61626 | P61626 | Lysozyme C | LYZ | 1027800 | 1 | Lysozyme C ( | 1 | 1 |
| P61106 | P61106 | Ras-related protein | RAB14 | 1022700 | 1 | Ras-related p | 1 | 1 |
| P84074 | P84074 | Neuron-specific calc | HPCA | 1013300 | 1 | Neuron-spec | 1 | 1 |
| P21695 | P21695 | Glycerol-3-phosphat | GPD1 | 1009300 | 1 | Glycerol-3-pl | 1 | 1 |
| Q16718 | Q16718 | NADH dehydrogenas | NDUFA5 | 1004600 | 1 | NADH dehydr | 1 | 1 |
| Q9UPY8 | Q9UPY8 | Microtubule-associa | MAPRE3 | 994630 | 1 | Microtubule- | 1 | 1 |

| | | | | | | | | |
|---|---|---|---|---|---|---|---|---|
| Q96E39;P38159 | Q96E39;P38159 | RNA binding motif p | RBMXL1;RBMX | 992320 | 2 | RNA binding | 2 | 2;2 |
| Q9NRG1 | Q9NRG1 | Phosphoribosyltrans | PRTFDC1 | 986080 | 1 | Phosphoribos | 1 | 1 |
| P38606 | P38606 | V-type proton ATPas | ATP6V1A | 980840 | 3 | V-type protoı | 1 | 3 |
| P10644 | P10644 | cAMP-dependent pr | PRKAR1A | 980660 | 1 | cAMP-depen | 1 | 1 |
| Q96PU8 | Q96PU8 | Protein quaking | QKI | 973050 | 1 | Protein quaki | 1 | 1 |
| Q9ULC3 | Q9ULC3 | Ras-related protein | RAB23 | 954530 | 1 | Ras-related p | 1 | 1 |
| Q8IZP0 | Q8IZP0 | Abl interactor 1 | ABI1 | 947210 | 1 | Abl interacto | 1 | 1 |
| P81605 | P81605 | Dermcidin;Survival-p | DCD | 937190 | 2 | Dermcidin OS | 1 | 1 |
| Q96GK7;Q6P2I3 | Q96GK7;Q6P2I3 | Fumarylacetoacetat | FAHD2A;FAHD2 | 936160 | 1 | Fumarylacet | 2 | 1;1 |
| P35914 | P35914 | Hydroxymethylgluta | HMGCL | 933410 | 0 | Hydroxymeth | 1 | 1 |
| P45974 | P45974 | Ubiquitin carboxyl-te | USP5 | 931350 | 1 | Ubiquitin car | 1 | 1 |
| P28074 | P28074 | Proteasome subunit | PSMB5 | 919600 | 1 | Proteasome | 1 | 1 |
| P26599;O95758 | P26599;O95758 | Polypyrimidine tract | PTBP1;PTBP3 | 916360 | 3 | Polypyrimidi | 2 | 2;1 |
| P09382 | P09382 | Galectin-1 | LGALS1 | 912100 | 2 | Galectin-1 O | 1 | 2 |
| Q8WXA9 | Q8WXA9 | Splicing regulatory g | SREK1 | 908790 | 1 | Splicing regu | 1 | 2 |
| Q8WXF0;O75494 | Q8WXF0;O75494 | Serine/arginine-rich | SRSF12;SRSF10 | 902540 | 1 | Serine/argini | 2 | 1;1 |
| O75083 | O75083 | WD repeat-containi | WDR1 | 878660 | 1 | WD repeat-c | 1 | 1 |
| Q86V81 | Q86V81 | THO complex subun | ALYREF | 871710 | 1 | THO complex | 1 | 1 |
| Q99439 | Q99439 | Calponin-2 | CNN2 | 853420 | 1 | Calponin-2 O | 1 | 2 |
| Q6UWP8 | Q6UWP8 | Suprabasin | SBSN | 823780 | 1 | Suprabasin C | 1 | 1 |
| P50452;P50453;P3074( | P50452;P50453;P3074( | Serpin B8;Serpin B9 | SERPINB8;SERP | 813790 | 1 | Serpin B8 OS | 6 | 1;1;1;1;1;1 |
| Q96EP5 | Q96EP5 | DAZ-associated prot | DAZAP1 | 807420 | 0 | DAZ-associat | 1 | 1 |
| P21579 | P21579 | Synaptotagmin-1 | SYT1 | 798300 | 1 | Synaptotagm | 1 | 1 |
| P48444 | P48444 | Coatomer subunit d | ARCN1 | 796940 | 2 | Coatomer su | 1 | 2 |
| O95218 | O95218 | Zinc finger Ran-binc | ZRANB2 | 790690 | 3 | Zinc finger R | 1 | 2 |
| P31948 | P31948 | Stress-induced-phos | STIP1 | 790000 | 1 | Stress-induce | 1 | 1 |
| O75410;Q9Y6A5 | O75410;Q9Y6A5 | Transforming acidic | TACC1;TACC3 | 779230 | 1 | Transforming | 2 | 1;1 |
| Q96FC7 | Q96FC7 | Phytanoyl-CoA hydro | PHYHIPL | 779000 | 1 | Phytanoyl-Co | 1 | 1 |
| Q15517 | Q15517 | Corneodesmosin | CDSN | 768220 | 2 | Corneodesm | 1 | 1 |
| P34096 | P34096 | Ribonuclease 4 | RNASE4 | 767770 | 1 | Ribonuclease | 1 | 1 |
| P01116;P01112 | P01116;P01112 | GTPase KRas;GTPas | KRAS;HRAS | 765570 | 1 | GTPase KRas | 2 | 1;1 |
| Q08188 | Q08188 | Protein-glutamine g | TGM3 | 763670 | 1 | Protein-gluta | 1 | 1 |
| P09651 | P09651 | Heterogeneous nucl | HNRNPA1 | 746640 | 2 | Heterogeneo | 1 | 1 |
| Q7L4I2 | Q7L4I2 | Arginine/serine-rich | RSRC2 | 739420 | 0 | Arginine/seri | 1 | 1 |
| Q9Y5L4 | Q9Y5L4 | Mitochondrial impo | TIMM13 | 736820 | 1 | Mitochondria | 1 | 1 |
| O43426 | O43426 | Synaptojanin-1 | SYNJ1 | 728730 | 2 | Synaptojanin | 1 | 1 |
| Q15185 | Q15185 | Prostaglandin E synt | PTGES3 | 727590 | 1 | Prostaglandi | 1 | 1 |
| Q96FW1 | Q96FW1 | Ubiquitin thioestera | OTUB1 | 721560 | 2 | Ubiquitin thi | 1 | 1 |

| | | | | | | | | |
|---|---|---|---|---|---|---|---|---|
| P46782 | P46782 | 40S ribosomal prote | RPS5 | 699040 | 1 | 40S ribosom | 1 | 1 |
| Q13526 | Q13526 | Peptidyl-prolyl cis-tr | PIN1 | 696080 | 2 | Peptidyl-prol | 1 | 1 |
| Q96RQ3 | Q96RQ3 | Methylcrotonoyl-Co | MCCC1 | 682320 | 1 | Methylcrotor | 1 | 1 |
| P23381 | P23381 | Tryptophan--tRNA li | WARS | 681830 | 1 | Tryptophan-- | 1 | 1 |
| Q13243 | Q13243 | Serine/arginine-rich | SRSF5 | 676640 | 3 | Serine/argini | 1 | 1 |
| P62857 | P62857 | 40S ribosomal prote | RPS28 | 674640 | 1 | 40S ribosom | 1 | 1 |
| Q14847 | Q14847 | LIM and SH3 domair | LASP1 | 674620 | 1 | LIM and SH3 | 1 | 1 |
| Q15287 | Q15287 | RNA-binding proteir | RNPS1 | 662840 | 2 | RNA-binding | 1 | 1 |
| P05455 | P05455 | Lupus La protein | SSB | 653850 | 2 | Lupus La pro | 1 | 1 |
| Q9UBX5 | Q9UBX5 | Fibulin-5 | FBLN5 | 650900 | 1 | Fibulin-5 OS= | 1 | 1 |
| P36873 | P36873 | Serine/threonine-pr | PPP1CC | 647760 | 2 | Serine/threo | 1 | 1 |
| Q16543 | Q16543 | Hsp90 co-chaperone | CDC37 | 641990 | 1 | Hsp90 co-cha | 1 | 1 |
| P37108 | P37108 | Signal recognition p | SRP14 | 639900 | 1 | Signal recogi | 1 | 1 |
| O43464 | O43464 | Serine protease HTF | HTRA2 | 636970 | 1 | Serine protea | 1 | 1 |
| O00178 | O00178 | GTP-binding protein | GTPBP1 | 636340 | 1 | GTP-binding | 1 | 1 |
| Q9H0U4;Q92928;P628 | Q9H0U4;Q92928;P628 | Ras-related protein | RAB1B;RAB1C; | 621510 | 1 | Ras-related | 3 | 1;1;1 |
| Q15369 | Q15369 | Transcription elonga | TCEB1 | 618660 | 1 | Transcriptior | 1 | 1 |
| Q9H074 | Q9H074 | Polyadenylate-bindi | PAIP1 | 616550 | 1 | Polyadenylat | 1 | 1 |
| P05386 | P05386 | 60S acidic ribosoma | RPLP1 | 609590 | 1 | 60S acidic rib | 1 | 1 |
| Q9NR31 | Q9NR31 | GTP-binding protein | SAR1A | 609250 | 1 | GTP-binding | 1 | 1 |
| Q99426 | Q99426 | Tubulin-folding cofa | TBCB | 609160 | 2 | Tubulin-foldi | 1 | 1 |
| P27635 | P27635 | 60S ribosomal prote | RPL10 | 603630 | 1 | 60S ribosom | 1 | 1 |
| P06730 | P06730 | Eukaryotic translatic | EIF4E | 582890 | 1 | Eukaryotic tr | 1 | 1 |
| O43776 | O43776 | Asparagine--tRNA li | NARS | 578310 | 1 | Asparagine-- | 1 | 1 |
| P05109 | P05109 | Protein S100-A8;Pro | S100A8 | 574680 | 1 | Protein S100 | 1 | 1 |
| P02452 | P02452 | Collagen alpha-1(I) | COL1A1 | 568060 | 1 | Collagen alpl | 1 | 1 |
| P49721 | P49721 | Proteasome subunit | PSMB2 | 565480 | 1 | Proteasome | 1 | 1 |
| P63162;P14678 | P63162;P14678 | Small nuclear ribon | SNRPN;SNRPB | 564850 | 1 | Small nuclea | 2 | 1;1 |
| P39060 | P39060 | Collagen alpha-1(XV | COL18A1 | 563840 | 1 | Collagen alpl | 1 | 1 |
| A6NMX2 | A6NMX2 | Eukaryotic translatic | EIF4E1B | 559750 | 1 | Eukaryotic tr | 1 | 1 |
| Q14677 | Q14677 | Clathrin interactor 1 | CLINT1 | 551140 | 1 | Clathrin inter | 1 | 1 |
| O43491 | O43491 | Band 4.1-like protei | EPB41L2 | 549690 | 1 | Band 4.1-like | 1 | 1 |
| Q9UHD1 | Q9UHD1 | Cysteine and histidi | CHORDC1 | 544870 | 1 | Cysteine and | 1 | 1 |
| P50440 | P50440 | Glycine amidinotrar | GATM | 540540 | 1 | Glycine amic | 1 | 1 |
| P04899 | P04899 | Guanine nucleotide- | GNAI2 | 536510 | 1 | Guanine nucl | 1 | 2 |
| Q8N684 | Q8N684 | Cleavage and polyac | CPSF7 | 534560 | 1 | Cleavage and | 1 | 1 |
| P35221;P26232 | P35221;P26232 | Catenin alpha-1;Cat | CTNNA1;CTNN | 534090 | 1 | Catenin alph | 2 | 1;1 |
| Q9Y490 | Q9Y490 | Talin-1 | TLN1 | 529230 | 1 | Talin-1 OS=H | 1 | 1 |

| | | | | | | | | | |
|---|---|---|---|---|---|---|---|---|---|
| P55145 | P55145 | Mesencephalic astro | MANF | 526990 | 2 | Mesencepha | 1 | | 2 |
| P30837 | P30837 | Aldehyde dehydroge | ALDH1B1 | 523470 | 2 | Aldehyde del | 1 | | 1 |
| Q14576 | Q14576 | ELAV-like protein 3 | ELAVL3 | 511290 | 2 | ELAV-like pro | 1 | | 2 |
| P61313 | P61313 | 60S ribosomal prote | RPL15 | 508000 | 1 | 60S ribosoma | 1 | | 1 |
| Q9C040 | Q9C040 | Tripartite motif-con | TRIM2 | 507800 | 1 | Tripartite mo | 1 | | 1 |
| Q13564 | Q13564 | NEDD8-activating er | NAE1 | 504360 | 1 | NEDD8-activa | 1 | | 1 |
| P62714 | P62714 | Serine/threonine-pr | PPP2CB | 488220 | 1 | Serine/threo | 1 | | 6 |
| Q9NZT1 | Q9NZT1 | Calmodulin-like prot | CALML5 | 487810 | 1 | Calmodulin-l | 1 | | 1 |
| Q14444 | Q14444 | Caprin-1 | CAPRIN1 | 481390 | 1 | Caprin-1 OS= | 1 | | 1 |
| P23368 | P23368 | NAD-dependent ma | ME2 | 475640 | 1 | NAD-depend | 1 | | 1 |
| Q12926;P26378 | Q12926;P26378 | ELAV-like protein 2; | ELAVL2;ELAVL4 | 474870 | 1 | ELAV-like pro | 2 | 1;1 | |
| Q99784 | Q99784 | Noelin | OLFM1 | 468580 | 1 | Noelin OS=H | 1 | | 1 |
| Q13185 | Q13185 | Chromobox protein | CBX3 | 458700 | 2 | Chromobox p | 1 | | 1 |
| Q13867 | Q13867 | Bleomycin hydrolase | BLMH | 457580 | 1 | Bleomycin hy | 1 | | 1 |
| P61289 | P61289 | Proteasome activato | PSME3 | 452830 | 1 | Proteasome | 1 | | 1 |
| P11498 | P11498 | Pyruvate carboxylas | PC | 452740 | 1 | Pyruvate carb | 1 | | 1 |
| Q99832 | Q99832 | T-complex protein 1 | CCT7 | 442240 | 1 | T-complex pr | 1 | | 1 |
| Q86SX6 | Q86SX6 | Glutaredoxin-relate | GLRX5 | 437340 | 2 | Glutaredoxin | 1 | | 2 |
| Q99719 | Q99719 | Septin-5 | 5-Sep | 432930 | 1 | Septin-5 OS= | 1 | | 1 |
| O14744 | O14744 | Protein arginine N-r | PRMT5 | 407540 | 2 | Protein argin | 1 | | 1 |
| O75223 | O75223 | Gamma-glutamylcy | GGCT | 407160 | 1 | Gamma-glut | 1 | | 1 |
| Q9UL25 | Q9UL25 | Ras-related protein | RAB21 | 401640 | 1 | Ras-related p | 1 | | 1 |
| Q9Y3F4 | Q9Y3F4 | Serine-threonine kir | STRAP | 396150 | 1 | Serine-threo | 1 | | 1 |
| P31321 | P31321 | cAMP-dependent pr | PRKAR1B | 394440 | 2 | cAMP-depen | 1 | | 1 |
| P60891;P21108;P11908 | P60891;P21108;P11908 | Ribose-phosphate p | PRPS1;PRPS1L1 | 387860 | 2 | Ribose-phos | 3 | 1;1;1 | |
| Q8WUH6 | Q8WUH6 | Transmembrane pro | TMEM263 | 367580 | 1 | Transmembr | 1 | | 1 |
| Q15560;P23193 | Q15560;P23193 | Transcription elonga | TCEA2;TCEA1 | 359310 | 1 | Transcription | 2 | 1;1 | |
| O43765 | O43765 | Small glutamine-ric | SGTA | 346820 | 1 | Small glutam | 1 | | 1 |
| Q9BUJ2 | Q9BUJ2 | Heterogeneous nucl | HNRNPUL1 | 339660 | 1 | Heterogeneo | 1 | | 1 |
| Q7RTV0 | Q7RTV0 | PHD finger-like dom | PHF5A | 334920 | 1 | PHD finger-li | 1 | | 1 |
| Q96HC4 | Q96HC4 | PDZ and LIM domain | PDLIM5 | 322910 | 1 | PDZ and LIM | 1 | | 1 |
| P60842;Q14240 | P60842;Q14240 | Eukaryotic initiation | EIF4A1;EIF4A2 | 322360 | 1 | Eukaryotic in | 2 | 1;1 | |
| Q14157 | Q14157 | Ubiquitin-associated | UBAP2L | 307370 | 2 | Ubiquitin-ass | 1 | | 1 |
| Q6ZVM7 | Q6ZVM7 | TOM1-like protein 2 | TOM1L2 | 307230 | 1 | TOM1-like pr | 1 | | 1 |
| P55735 | P55735 | Protein SEC13 homo | SEC13 | 301800 | 2 | Protein SEC1 | 1 | | 1 |
| O00116 | O00116 | Alkyldihydroxyaceto | AGPS | 277910 | 2 | Alkyldihydrox | 1 | | 2 |
| Q9BY32 | Q9BY32 | Inosine triphosphate | ITPA | 271460 | 1 | Inosine triph | 1 | | 1 |
| P27658 | P27658 | Collagen alpha-1(VI | COL8A1 | 263850 | 1 | Collagen alph | 1 | | 1 |

| | | | | | | | | |
|---|---|---|---|---|---|---|---|---|
| P17987 | P17987 | T-complex protein 1 | TCP1 | 249540 | 1 | T-complex pr | 1 | 1 |
| Q9UPQ3;Q96P47 | Q9UPQ3;Q96P47 | Arf-GAP with GTPas | AGAP1;AGAP3 | 248870 | 2 | Arf-GAP with | 2 | 1;1 |
| P55081 | P55081 | Microfibrillar-associ | MFAP1 | 244060 | 1 | Microfibrillar | 1 | 1 |
| Q9BXF6 | Q9BXF6 | Rab11 family-intera | RAB11FIP5 | 239350 | 1 | Rab11 family | 1 | 1 |
| P07900 | P07900 | Heat shock protein I | HSP90AA1 | 231110 | 1 | Heat shock p | 1 | 1 |
| Q5TAQ9 | Q5TAQ9 | DDB1- and CUL4-ass | DCAF8 | 230430 | 1 | DDB1- and Cl | 1 | 1 |
| P20073 | P20073 | Annexin A7 | ANXA7 | 230360 | 1 | Annexin A7 C | 1 | 1 |
| P49448;P00367 | P49448;P00367 | Glutamate dehydro | GLUD2;GLUD1 | 219430 | 1 | Glutamate d | 2 | 1;1 |
| Q9Y5K8 | Q9Y5K8 | V-type proton ATPas | ATP6V1D | 215940 | 1 | V-type protoi | 1 | 1 |
| P27448 | P27448 | MAP/microtubule a | MARK3 | 215870 | 1 | MAP/microtu | 1 | 1 |
| Q9UL46 | Q9UL46 | Proteasome activato | PSME2 | 178460 | 1 | Proteasome | 1 | 1 |
| O00422 | O00422 | Histone deacetylase | SAP18 | 131460 | 1 | Histone deac | 1 | 1 |
| Q96GR2 | Q96GR2 | Long-chain-fatty-aci | ACSBG1 | 121340 | 1 | Long-chain-f | 1 | 1 |
| O00233 | O00233 | 26S proteasome noi | PSMD9 | 0 | 1 | 26S proteaso | 1 | 1 |
| O15371 | O15371 | Eukaryotic translatio | EIF3D | 0 | 1 | Eukaryotic tr | 1 | 1 |
| O60613 | O60613 | 15 kDa selenoprotei | 15-Sep | 0 | 1 | 15 kDa selen | 1 | 1 |
| P00326 | P00326 | Alcohol dehydrogen | ADH1C | 0 | 1 | Alcohol dehy | 1 | 1 |
| P00450 | P00450 | Ceruloplasmin | CP | 0 | 1 | Ceruloplasmi | 1 | 1 |
| P13984 | P13984 | General transcriptio | GTF2F2 | 0 | 1 | General tran | 1 | 1 |
| P22234 | P22234 | Multifunctional prot | PAICS | 0 | 2 | Multifunction | 1 | 2 |
| P50991 | P50991 | T-complex protein 1 | CCT4 | 0 | 1 | T-complex pr | 1 | 1 |
| P55084 | P55084 | Trifunctional enzym | HADHB | 0 | 1 | Trifunctional | 1 | 1 |
| P55795 | P55795 | Heterogeneous nucl | HNRNPH2 | 0 | 1 | Heterogeneo | 1 | 3 |
| P78356 | P78356 | Phosphatidylinositol | PIP4K2B | 0 | 1 | Phosphatidyl | 1 | 2 |
| Q05519 | Q05519 | Serine/arginine-rich | SRSF11 | 0 | 1 | Serine/argini | 1 | 1 |
| Q13409 | Q13409 | Cytoplasmic dynein | DYNC1I2 | 0 | 1 | Cytoplasmic | 1 | 1 |
| Q14574 | Q14574 | Desmocollin-3 | DSC3 | 0 | 1 | Desmocollin- | 1 | 1 |
| Q8WUM0 | Q8WUM0 | Nuclear pore compl | NUP133 | 0 | 1 | Nuclear pore | 1 | 1 |
| Q92572 | Q92572 | AP-3 complex subur | AP3S1 | 0 | 1 | AP-3 comple | 1 | 1 |
| Q9UHD8 | Q9UHD8 | Septin-9 | 9-Sep | 0 | 1 | Septin-9 OS= | 1 | 1 |
| Q9UKV8 | Q9UKV8 | Protein argonaute-2 | AGO2 | 0 | 1 | Protein argoı | 1 | 1 |
| Q9Y303 | Q9Y303 | Putative N-acetylglu | AMDHD2 | 0 | 1 | N-acetylgluc | 1 | 1 |

| Peptide counts (razor+unique) | Peptide counts (unique) | Peptides | Razor + unique peptides | Unique peptides | Sequence coverage [%] | Unique + razor sequence coverage [%] | Unique sequence coverage [%] | Mol. weight [kDa] |
|---|---|---|---|---|---|---|---|---|
| 30 | 30 | 30 | 30 | 30 | 36.4 | 36.4 | 36.4 | 38.714 |
| 8;1 | 6;0 | 8 | 8 | 6 | 33.1 | 33.1 | 20.4 | 15.257 |
| 9;9;5;5;5;5;4; | 9;9;5;5;5;5;4; | 9 | 9 | 9 | 34.1 | 34.1 | 34.1 | 41.792 |
| 7 | 7 | 7 | 7 | 7 | 38.8 | 38.8 | 38.8 | 17.718 |
| 16 | 16 | 16 | 16 | 16 | 38.6 | 38.6 | 38.6 | 57.936 |
| 14 | 9 | 14 | 14 | 9 | 35.5 | 35.5 | 25 | 62.293 |
| 20;7;2;1;1 | 17;4;0;0;0 | 20 | 20 | 17 | 40.7 | 40.7 | 34.8 | 70.897 |
| 4 | 4 | 4 | 4 | 4 | 9.9 | 9.9 | 9.9 | 42.064 |
| 7;7 | 3;3 | 7 | 7 | 3 | 19.7 | 19.7 | 10.2 | 50.184 |
| 4 | 4 | 4 | 4 | 4 | 18.2 | 18.2 | 18.2 | 30.791 |
| 12;4 | 12;4 | 12 | 12 | 12 | 35.4 | 35.4 | 35.4 | 43.295 |
| 7 | 7 | 7 | 7 | 7 | 40.4 | 40.4 | 40.4 | 21.676 |
| 9;7 | 9;7 | 9 | 9 | 9 | 35.1 | 35.1 | 35.1 | 38.604 |
| 2 | 2 | 3 | 2 | 2 | 21.1 | 14.3 | 14.3 | 16.055 |
| 16;3 | 15;2 | 16 | 16 | 15 | 24.5 | 24.5 | 22.9 | 94.33 |
| 7 | 7 | 7 | 7 | 7 | 12.5 | 12.5 | 12.5 | 79.685 |
| 6 | 5 | 6 | 6 | 5 | 23.4 | 23.4 | 19.2 | 42.644 |
| 7;3 | 7;3 | 7 | 7 | 7 | 30.5 | 30.5 | 30.5 | 26.145 |
| 7 | 7 | 7 | 7 | 7 | 18 | 18 | 18 | 43.447 |
| 7 | 7 | 7 | 7 | 7 | 26.6 | 26.6 | 26.6 | 35.503 |
| 5 | 5 | 5 | 5 | 5 | 22.3 | 22.3 | 22.3 | 39.42 |
| 7;3;2;1 | 7;3;2;1 | 7 | 7 | 7 | 29.4 | 29.4 | 29.4 | 37.377 |
| 5 | 5 | 5 | 5 | 5 | 49.1 | 49.1 | 49.1 | 17.818 |
| 6 | 6 | 6 | 6 | 6 | 37.2 | 37.2 | 37.2 | 16.06 |
| 1 | 1 | 2 | 1 | 1 | 4.2 | 4.2 | 4.2 | 44.501 |
| 4 | 4 | 4 | 4 | 4 | 32 | 32 | 32 | 11.367 |
| 3 | 3 | 3 | 3 | 3 | 14 | 14 | 14 | 13.696 |
| 9;1 | 9;1 | 9 | 9 | 9 | 32.8 | 32.8 | 32.8 | 47.371 |
| 5 | 5 | 5 | 5 | 5 | 11 | 11 | 11 | 56.381 |
| 5 | 5 | 5 | 5 | 5 | 30.3 | 30.3 | 30.3 | 24.205 |
| 6;1;1 | 6;1;1 | 6 | 6 | 6 | 25.9 | 25.9 | 25.9 | 29.597 |
| 7 | 7 | 7 | 7 | 7 | 44.6 | 44.6 | 44.6 | 20.567 |
| 2 | 2 | 2 | 2 | 2 | 22.5 | 22.5 | 22.5 | 16.273 |
| 4 | 4 | 4 | 4 | 4 | 31.7 | 31.7 | 31.7 | 19.329 |

| | | | | | | | | | |
|---|---|---|---|---|---|---|---|---|---|
| 1;1 | 1;1 | | 3 | 1 | 1 | 5.1 | 1.7 | 1.7 | 71.027 |
| 2 | 2 | | 2 | 2 | 2 | 10 | 10 | 10 | 24.261 |
| 1 | 1 | | 1 | 1 | 1 | 8.1 | 8.1 | 8.1 | 14.551 |
| 11 | 11 | | 12 | 11 | 11 | 21.9 | 19.4 | 19.4 | 72.332 |
| 6 | 6 | | 6 | 6 | 6 | 11 | 11 | 11 | 85.424 |
| 4 | 4 | | 4 | 4 | 4 | 41.5 | 41.5 | 41.5 | 13.527 |
| 3 | 3 | | 3 | 3 | 3 | 17.4 | 17.4 | 17.4 | 24.579 |
| 7 | 3 | | 7 | 7 | 3 | 18.2 | 18.2 | 9.4 | 58.687 |
| 4 | 4 | | 4 | 4 | 4 | 24 | 24 | 24 | 13.742 |
| 6 | 5 | | 6 | 6 | 5 | 15 | 15 | 12.8 | 74.111 |
| 19 | 19 | | 19 | 19 | 19 | 8.5 | 8.5 | 8.5 | 331.77 |
| 5 | 5 | | 5 | 5 | 5 | 8.1 | 8.1 | 8.1 | 90.583 |
| 4 | 4 | | 4 | 4 | 4 | 11.2 | 11.2 | 11.2 | 54.177 |
| 2 | 2 | | 2 | 2 | 2 | 16.1 | 16.1 | 16.1 | 15.747 |
| 3 | 3 | | 3 | 3 | 3 | 17.8 | 17.8 | 17.8 | 21.868 |
| 9 | 9 | | 9 | 9 | 9 | 19.2 | 19.2 | 19.2 | 67.568 |
| 2 | 2 | | 2 | 2 | 2 | 15.1 | 15.1 | 15.1 | 9.7251 |
| 4 | 4 | | 4 | 4 | 4 | 16.4 | 16.4 | 16.4 | 28.315 |
| 3 | 3 | | 3 | 3 | 3 | 14.1 | 14.1 | 14.1 | 22.11 |
| 3 | 3 | | 3 | 3 | 3 | 20.3 | 20.3 | 20.3 | 24.636 |
| 4 | 4 | | 4 | 4 | 4 | 11.2 | 11.2 | 11.2 | 33.117 |
| 4;2;2 | 4;2;2 | | 4 | 4 | 4 | 16.4 | 16.4 | 16.4 | 38.58 |
| 3;1 | 3;1 | | 3 | 3 | 3 | 24.7 | 24.7 | 24.7 | 19.3 |
| 7;7;6;4;3;2;2; | 7;7;6;4;3;2;2; | | 7 | 7 | 7 | 18.9 | 18.9 | 18.9 | 49.895 |
| 3 | 3 | | 3 | 3 | 3 | 19.2 | 19.2 | 19.2 | 16.445 |
| 3 | 3 | | 3 | 3 | 3 | 12.3 | 12.3 | 12.3 | 39.233 |
| 6 | 5 | | 6 | 6 | 5 | 34.3 | 34.3 | 28.3 | 32.949 |
| 5 | 3 | | 5 | 5 | 3 | 16.4 | 16.4 | 11.1 | 43.727 |
| 2 | 2 | | 4 | 2 | 2 | 13 | 7.9 | 7.9 | 46.513 |
| 9 | 9 | | 9 | 9 | 9 | 22.4 | 22.4 | 22.4 | 59.75 |
| 5 | 5 | | 5 | 5 | 5 | 20.6 | 20.6 | 20.6 | 27.744 |
| 3;3 | 3;3 | | 3 | 3 | 3 | 22.9 | 22.9 | 22.9 | 27.872 |
| 3 | 3 | | 3 | 3 | 3 | 7.4 | 7.4 | 7.4 | 59.755 |
| 3 | 3 | | 3 | 3 | 3 | 30.5 | 30.5 | 30.5 | 14.787 |
| 5 | 5 | | 8 | 5 | 5 | 19.5 | 11.4 | 11.4 | 61.963 |
| 2 | 1 | | 2 | 2 | 1 | 6.6 | 6.6 | 3 | 36.638 |
| 5 | 4 | | 5 | 5 | 4 | 27.5 | 27.5 | 24.2 | 36.053 |
| 4 | 4 | | 4 | 4 | 4 | 14.8 | 14.8 | 14.8 | 31.387 |

| | | | | | | | | |
|---|---|---|---|---|---|---|---|---|
| 2 | 2 | 2 | 2 | 2 | 11.7 | 11.7 | 11.7 | 23.277 |
| 5 | 5 | 5 | 5 | 5 | 10.9 | 10.9 | 10.9 | 65.853 |
| 6 | 6 | 6 | 6 | 6 | 15.2 | 15.2 | 15.2 | 44.739 |
| 8 | 8 | 8 | 8 | 8 | 24.2 | 24.2 | 24.2 | 56.559 |
| 3 | 3 | 3 | 3 | 3 | 15.7 | 15.7 | 15.7 | 28.68 |
| 2 | 2 | 2 | 2 | 2 | 4.2 | 4.2 | 4.2 | 72.691 |
| 4;4;2 | 3;3;1 | 4 | 4 | 3 | 31.5 | 31.5 | 21.5 | 20.697 |
| 3;1 | 3;1 | 3 | 3 | 3 | 21.1 | 21.1 | 21.1 | 18.502 |
| 2 | 2 | 2 | 2 | 2 | 12.2 | 12.2 | 12.2 | 25.476 |
| 4 | 3 | 4 | 4 | 3 | 17.6 | 17.6 | 13.5 | 27.745 |
| 4 | 4 | 4 | 4 | 4 | 14.5 | 14.5 | 14.5 | 54.636 |
| 2 | 2 | 2 | 2 | 2 | 4.3 | 4.3 | 4.3 | 51.556 |
| 1 | 1 | 1 | 1 | 1 | 4.1 | 4.1 | 4.1 | 30.54 |
| 5 | 5 | 5 | 5 | 5 | 16.8 | 16.8 | 16.8 | 41.92 |
| 2 | 2 | 2 | 2 | 2 | 6.2 | 6.2 | 6.2 | 48.442 |
| 1;1;1;1;1;1;1; | 1;1;1;1;1;1;1; | 1 | 1 | 1 | 14.8 | 14.8 | 14.8 | 13.936 |
| 5 | 5 | 5 | 5 | 5 | 16.7 | 16.7 | 16.7 | 37.429 |
| 3 | 3 | 3 | 3 | 3 | 39.3 | 39.3 | 39.3 | 15.55 |
| 5 | 4 | 6 | 5 | 4 | 17.2 | 13.4 | 11.2 | 47.504 |
| 6 | 6 | 6 | 6 | 6 | 8.5 | 8.5 | 8.5 | 95.337 |
| 6 | 6 | 9 | 6 | 6 | 24.3 | 17.3 | 17.3 | 62.183 |
| 2 | 2 | 2 | 2 | 2 | 14.9 | 14.9 | 14.9 | 16.561 |
| 7 | 7 | 7 | 7 | 7 | 14.1 | 14.1 | 14.1 | 61.054 |
| 3 | 3 | 3 | 3 | 3 | 11.2 | 11.2 | 11.2 | 50.976 |
| 4 | 4 | 4 | 4 | 4 | 23.8 | 23.8 | 23.8 | 26.227 |
| 3 | 3 | 3 | 3 | 3 | 11.4 | 11.4 | 11.4 | 29.945 |
| 3 | 3 | 3 | 3 | 3 | 19.3 | 19.3 | 19.3 | 26.688 |
| 6 | 6 | 6 | 6 | 6 | 23.6 | 23.6 | 23.6 | 39.455 |
| 3 | 3 | 3 | 3 | 3 | 15.3 | 15.3 | 15.3 | 32.854 |
| 1 | 1 | 1 | 1 | 1 | 10.9 | 10.9 | 10.9 | 13.281 |
| 5 | 5 | 5 | 5 | 5 | 7.5 | 7.5 | 7.5 | 116.03 |
| 6;1 | 5;0 | 6 | 6 | 5 | 20.7 | 20.7 | 16.6 | 47.268 |
| 2 | 2 | 2 | 2 | 2 | 14 | 14 | 14 | 17.779 |
| 4 | 4 | 5 | 4 | 4 | 14.4 | 11.7 | 11.7 | 62.846 |
| 2 | 2 | 2 | 2 | 2 | 15.7 | 15.7 | 15.7 | 14.46 |
| 4 | 4 | 5 | 4 | 4 | 19.6 | 15.4 | 15.4 | 47.168 |
| 4 | 4 | 4 | 4 | 4 | 19.9 | 19.9 | 19.9 | 32.233 |
| 8;2;1;1;1;1 | 2;0;0;0;0;0 | 8 | 8 | 2 | 26.4 | 26.4 | 6.1 | 49.67 |

| | | | | | | | | |
|---|---|---|---|---|---|---|---|---|
| 4;3 | 4;3 | 4 | 4 | 4 | 12.9 | 12.9 | 12.9 | 56.5 |
| 3 | 3 | 3 | 3 | 3 | 5 | 5 | 5 | 81.889 |
| 2 | 2 | 2 | 2 | 2 | 12.6 | 12.6 | 12.6 | 13.373 |
| 2 | 2 | 2 | 2 | 2 | 11.2 | 11.2 | 11.2 | 33.742 |
| 2 | 2 | 2 | 2 | 2 | 8.3 | 8.3 | 8.3 | 42.183 |
| 1 | 1 | 1 | 1 | 1 | 9 | 9 | 9 | 17.04 |
| 5 | 5 | 5 | 5 | 5 | 15.2 | 15.2 | 15.2 | 44.76 |
| 2 | 2 | 4 | 2 | 2 | 15.7 | 10.4 | 10.4 | 42.613 |
| 1 | 1 | 1 | 1 | 1 | 6.4 | 6.4 | 6.4 | 31.236 |
| 8 | 8 | 8 | 8 | 8 | 16.5 | 16.5 | 16.5 | 73.68 |
| 6 | 4 | 6 | 6 | 4 | 22.3 | 22.3 | 17 | 42.293 |
| 5;4;3;2;1 | 5;4;3;2;1 | 5 | 5 | 5 | 11 | 11 | 11 | 70.67 |
| 1;1;1;1 | 1;1;1;1 | 1 | 1 | 1 | 11.5 | 11.5 | 11.5 | 12.059 |
| 2;1 | 2;1 | 2 | 2 | 2 | 20.9 | 20.9 | 20.9 | 13.015 |
| 7 | 7 | 7 | 7 | 7 | 10.8 | 10.8 | 10.8 | 113.75 |
| 4;4;2 | 4;4;2 | 4 | 4 | 4 | 29.3 | 29.3 | 29.3 | 20.825 |
| 3 | 3 | 3 | 3 | 3 | 22.4 | 22.4 | 22.4 | 21.863 |
| 3 | 3 | 3 | 3 | 3 | 13 | 13 | 13 | 30.381 |
| 1;1;1 | 1;1;1 | 2 | 1 | 1 | 10.9 | 5.2 | 5.2 | 21.45 |
| 3 | 3 | 3 | 3 | 3 | 2.1 | 2.1 | 2.1 | 305.48 |
| 3 | 3 | 3 | 3 | 3 | 4 | 4 | 4 | 91.925 |
| 2 | 2 | 6 | 2 | 2 | 17.1 | 7.6 | 7.6 | 50.47 |
| 3 | 3 | 3 | 3 | 3 | 12.8 | 12.8 | 12.8 | 40.513 |
| 2 | 2 | 2 | 2 | 2 | 25 | 25 | 25 | 14.865 |
| 2;2;1;1 | 1;1;0;0 | 2 | 2 | 1 | 11 | 11 | 5.2 | 21.258 |
| 2 | 2 | 2 | 2 | 2 | 14.6 | 14.6 | 14.6 | 14.839 |
| 4 | 4 | 4 | 4 | 4 | 16.9 | 16.9 | 16.9 | 39.594 |
| 2;2 | 2;2 | 2 | 2 | 2 | 5.6 | 5.6 | 5.6 | 43.057 |
| 1 | 1 | 1 | 1 | 1 | 19.6 | 19.6 | 19.6 | 10.275 |
| 1 | 1 | 1 | 1 | 1 | 22.5 | 22.5 | 22.5 | 7.8501 |
| 3 | 3 | 3 | 3 | 3 | 12.4 | 12.4 | 12.4 | 53.5 |
| 3 | 3 | 3 | 3 | 3 | 8.7 | 8.7 | 8.7 | 39.591 |
| 3;0 | 3;0 | 4 | 3 | 3 | 17.8 | 13.8 | 13.8 | 28.302 |
| 6 | 1 | 6 | 6 | 1 | 24.6 | 24.6 | 4.2 | 35.594 |
| 4 | 4 | 4 | 4 | 4 | 23 | 23 | 23 | 22.142 |
| 4;2 | 4;2 | 4 | 4 | 4 | 31.1 | 31.1 | 31.1 | 16.93 |
| 3 | 3 | 3 | 3 | 3 | 17.1 | 17.1 | 17.1 | 23.742 |
| 2 | 2 | 2 | 2 | 2 | 7.5 | 7.5 | 7.5 | 36.426 |

| | | | | | | | | |
|---|---|---|---|---|---|---|---|---|
| 4 | 4 | 4 | 4 | 4 | 50.4 | 50.4 | 50.4 | 12.784 |
| 4;1 | 4;1 | 4 | 4 | 4 | 14.7 | 14.7 | 14.7 | 41.515 |
| 2 | 2 | 2 | 2 | 2 | 12.1 | 12.1 | 12.1 | 30.84 |
| 4 | 4 | 4 | 4 | 4 | 10.2 | 10.2 | 10.2 | 47.716 |
| 2 | 2 | 2 | 2 | 2 | 7.4 | 7.4 | 7.4 | 36.263 |
| 5 | 5 | 5 | 5 | 5 | 6.3 | 6.3 | 6.3 | 117.36 |
| 6 | 6 | 6 | 6 | 6 | 19.9 | 19.9 | 19.9 | 38.534 |
| 2 | 2 | 2 | 2 | 2 | 19.2 | 19.2 | 19.2 | 14.463 |
| 1 | 1 | 1 | 1 | 1 | 2.1 | 2.1 | 2.1 | 69.602 |
| 2 | 2 | 2 | 2 | 2 | 19.5 | 19.5 | 19.5 | 15.423 |
| 2 | 2 | 2 | 2 | 2 | 1.1 | 1.1 | 1.1 | 270.63 |
| 1 | 1 | 1 | 1 | 1 | 8.7 | 8.7 | 8.7 | 14.395 |
| 2 | 2 | 2 | 2 | 2 | 4.4 | 4.4 | 4.4 | 54.861 |
| 4 | 2 | 4 | 4 | 2 | 14.5 | 14.5 | 7.1 | 49.229 |
| 6 | 6 | 6 | 6 | 6 | 9.3 | 9.3 | 9.3 | 81.744 |
| 3;1 | 3;1 | 3 | 3 | 3 | 7.7 | 7.7 | 7.7 | 58.951 |
| 3 | 3 | 3 | 3 | 3 | 8.7 | 8.7 | 8.7 | 56.94 |
| 2;1 | 2;1 | 2 | 2 | 2 | 12.4 | 12.4 | 12.4 | 17.258 |
| 5 | 5 | 5 | 5 | 5 | 16.6 | 16.6 | 16.6 | 50.817 |
| 4 | 4 | 4 | 4 | 4 | 10.5 | 10.5 | 10.5 | 56.447 |
| 1 | 1 | 1 | 1 | 1 | 3 | 3 | 3 | 67.877 |
| 3 | 3 | 3 | 3 | 3 | 21.6 | 21.6 | 21.6 | 16.368 |
| 4 | 4 | 4 | 4 | 4 | 20.6 | 20.6 | 20.6 | 31.35 |
| 3 | 3 | 3 | 3 | 3 | 8.1 | 8.1 | 8.1 | 50.965 |
| 1 | 1 | 1 | 1 | 1 | 15.9 | 15.9 | 15.9 | 7.9331 |
| 4 | 4 | 4 | 4 | 4 | 7.8 | 7.8 | 7.8 | 79.467 |
| 2 | 2 | 2 | 2 | 2 | 12.8 | 12.8 | 12.8 | 33.665 |
| 3 | 3 | 3 | 3 | 3 | 10.8 | 10.8 | 10.8 | 41.569 |
| 3;1;1;1;1;1;1; | 2;0;0;0;0;0; | 3 | 3 | 2 | 9.9 | 9.9 | 6.8 | 40.05 |
| 1;0;0 | 1;0;0 | 2 | 1 | 1 | 6.6 | 3 | 3 | 36.688 |
| 2 | 2 | 2 | 2 | 2 | 5.8 | 5.8 | 5.8 | 51.712 |
| 2 | 2 | 2 | 2 | 2 | 16.3 | 16.3 | 16.3 | 16.941 |
| 1;1;1;1;1;1;1; | 1;1;1;1;1;1; | 1 | 1 | 1 | 5.3 | 5.3 | 5.3 | 23.461 |
| 1 | 1 | 1 | 1 | 1 | 5.4 | 5.4 | 5.4 | 21.397 |
| 2 | 2 | 2 | 2 | 2 | 11.1 | 11.1 | 11.1 | 24.423 |
| 1 | 1 | 2 | 1 | 1 | 9.8 | 5.7 | 5.7 | 27.764 |
| 3 | 3 | 3 | 3 | 3 | 13.2 | 13.2 | 13.2 | 29.995 |
| 5 | 5 | 6 | 5 | 5 | 11 | 9.3 | 9.3 | 96.864 |

| | | | | | | | | |
|---|---|---|---|---|---|---|---|---|
| 2 | 2 | 2 | 2 | 2 | 7.7 | 7.7 | 7.7 | 30.211 |
| 3 | 2 | 3 | 3 | 2 | 8.7 | 8.7 | 5.2 | 39.586 |
| 4 | 4 | 5 | 4 | 4 | 28.7 | 22.7 | 22.7 | 32.922 |
| 4 | 4 | 4 | 4 | 4 | 12.1 | 12.1 | 12.1 | 50.679 |
| 3 | 3 | 3 | 3 | 3 | 13.2 | 13.2 | 13.2 | 35.079 |
| 1 | 1 | 1 | 1 | 1 | 4 | 4 | 4 | 30.188 |
| 2 | 2 | 2 | 2 | 2 | 6.2 | 6.2 | 6.2 | 44.614 |
| 1 | 1 | 1 | 1 | 1 | 8.8 | 8.8 | 8.8 | 27.366 |
| 2 | 2 | 2 | 2 | 2 | 7.5 | 7.5 | 7.5 | 34.006 |
| 3 | 3 | 3 | 3 | 3 | 14.3 | 14.3 | 14.3 | 35.329 |
| 3 | 3 | 3 | 3 | 3 | 10.5 | 10.5 | 10.5 | 48.121 |
| 1 | 1 | 1 | 1 | 1 | 13 | 13 | 13 | 10.803 |
| 2;2 | 2;2 | 2 | 2 | 2 | 14.6 | 14.6 | 14.6 | 16.46 |
| 3 | 3 | 3 | 3 | 3 | 10.4 | 10.4 | 10.4 | 38.434 |
| 1 | 1 | 1 | 1 | 1 | 6.7 | 6.7 | 6.7 | 18.506 |
| 2 | 2 | 2 | 2 | 2 | 13.1 | 13.1 | 13.1 | 19.667 |
| 1 | 1 | 1 | 1 | 1 | 5.4 | 5.4 | 5.4 | 28.087 |
| 2 | 2 | 2 | 2 | 2 | 23.8 | 23.8 | 23.8 | 13.916 |
| 2 | 2 | 2 | 2 | 2 | 8.5 | 8.5 | 8.5 | 33.31 |
| 2 | 2 | 3 | 2 | 2 | 15.7 | 11.8 | 11.8 | 29.174 |
| 4 | 4 | 4 | 4 | 4 | 18.1 | 18.1 | 18.1 | 36.112 |
| 1 | 1 | 1 | 1 | 1 | 4.9 | 4.9 | 4.9 | 55.405 |
| 1 | 1 | 1 | 1 | 1 | 24.7 | 24.7 | 24.7 | 10.366 |
| 2 | 2 | 2 | 2 | 2 | 7.8 | 7.8 | 7.8 | 31.324 |
| 2 | 2 | 2 | 2 | 2 | 12.4 | 12.4 | 12.4 | 19.463 |
| 1 | 1 | 1 | 1 | 1 | 4.6 | 4.6 | 4.6 | 36.249 |
| 1 | 1 | 1 | 1 | 1 | 5.5 | 5.5 | 5.5 | 18.012 |
| 1 | 1 | 1 | 1 | 1 | 3.5 | 3.5 | 3.5 | 40.949 |
| 2 | 2 | 2 | 2 | 2 | 14.8 | 14.8 | 14.8 | 23.423 |
| 2 | 2 | 2 | 2 | 2 | 26.3 | 26.3 | 26.3 | 13.604 |
| 2 | 2 | 2 | 2 | 2 | 10.2 | 10.2 | 10.2 | 40.798 |
| 2 | 2 | 2 | 2 | 2 | 10.5 | 10.5 | 10.5 | 28.024 |
| 3 | 3 | 3 | 3 | 3 | 11.3 | 11.3 | 11.3 | 22.591 |
| 1 | 1 | 1 | 1 | 1 | 2.8 | 2.8 | 2.8 | 43.182 |
| 2 | 2 | 2 | 2 | 2 | 8.7 | 8.7 | 8.7 | 30.241 |
| 1;1 | 1;1 | 1 | 1 | 1 | 8.5 | 8.5 | 8.5 | 18.898 |
| 1 | 1 | 1 | 1 | 1 | 3.4 | 3.4 | 3.4 | 57.214 |
| 3 | 3 | 3 | 3 | 3 | 7.8 | 7.8 | 7.8 | 58.486 |

| | | | | | | | | |
|---|---|---|---|---|---|---|---|---|
| 5 | 5 | 5 | 5 | 5 | 15 | 15 | 15 | 59.875 |
| 2 | 2 | 2 | 2 | 2 | 18.4 | 18.4 | 18.4 | 18.431 |
| 2 | 2 | 2 | 2 | 2 | 16.6 | 16.6 | 16.6 | 16.32 |
| 1 | 1 | 1 | 1 | 1 | 3.9 | 3.9 | 3.9 | 27.843 |
| 2 | 2 | 2 | 2 | 2 | 6.4 | 6.4 | 6.4 | 47.535 |
| 2 | 2 | 2 | 2 | 2 | 7.1 | 7.1 | 7.1 | 31.629 |
| 3;3 | 3;3 | 3 | 3 | 3 | 11.9 | 11.9 | 11.9 | 21.768 |
| 1 | 1 | 1 | 1 | 1 | 3 | 3 | 3 | 47.655 |
| 3 | 3 | 3 | 3 | 3 | 5.5 | 5.5 | 5.5 | 89.321 |
| 2;2;1 | 2;2;1 | 2 | 2 | 2 | 22.7 | 22.7 | 22.7 | 16.773 |
| 2 | 2 | 2 | 2 | 2 | 3.7 | 3.7 | 3.7 | 76.613 |
| 1 | 1 | 1 | 1 | 1 | 4.6 | 4.6 | 4.6 | 43.566 |
| 2 | 2 | 2 | 2 | 2 | 13.7 | 13.7 | 13.7 | 23.207 |
| 3 | 3 | 3 | 3 | 3 | 20.3 | 20.3 | 20.3 | 18.042 |
| 1 | 1 | 1 | 1 | 1 | 1.7 | 1.7 | 1.7 | 92.468 |
| 5 | 5 | 5 | 5 | 5 | 32.9 | 32.9 | 32.9 | 23.489 |
| 1 | 1 | 3 | 1 | 1 | 9.1 | 3.1 | 3.1 | 47.036 |
| 1 | 1 | 1 | 1 | 1 | 9 | 9 | 9 | 13.53 |
| 2;2;2;2;2;2;2; | 2;2;2;2;2;2; | 2 | 2 | 2 | 19.8 | 19.8 | 19.8 | 13.952 |
| 1 | 1 | 1 | 1 | 1 | 5.8 | 5.8 | 5.8 | 23.584 |
| 1 | 1 | 1 | 1 | 1 | 20 | 20 | 20 | 8.3047 |
| 3 | 3 | 3 | 3 | 3 | 13.9 | 13.9 | 13.9 | 39.962 |
| 3;3;1 | 3;3;1 | 3 | 3 | 3 | 22.8 | 22.8 | 22.8 | 19.794 |
| 2 | 2 | 2 | 2 | 2 | 7.4 | 7.4 | 7.4 | 36.091 |
| 1 | 1 | 1 | 1 | 1 | 7.7 | 7.7 | 7.7 | 15.807 |
| 3 | 3 | 3 | 3 | 3 | 7.2 | 7.2 | 7.2 | 59.379 |
| 1 | 1 | 1 | 1 | 1 | 2.2 | 2.2 | 2.2 | 45.191 |
| 2 | 2 | 2 | 2 | 2 | 14.2 | 14.2 | 14.2 | 23.545 |
| 1 | 1 | 1 | 1 | 1 | 0.6 | 0.6 | 0.6 | 159 |
| 4 | 4 | 4 | 4 | 4 | 10.6 | 10.6 | 10.6 | 46.276 |
| 2 | 2 | 2 | 2 | 2 | 8.4 | 8.4 | 8.4 | 41 |
| 3 | 0 | 12 | 3 | 0 | 21.5 | 7.8 | 0 | 60.066 |
| 3 | 3 | 3 | 3 | 3 | 10.3 | 10.3 | 10.3 | 44.965 |
| 1 | 1 | 1 | 1 | 1 | 17.9 | 17.9 | 17.9 | 15.394 |
| 1 | 1 | 1 | 1 | 1 | 0.9 | 0.9 | 0.9 | 110.56 |
| 1 | 1 | 1 | 1 | 1 | 7.1 | 7.1 | 7.1 | 17.695 |
| 2 | 2 | 2 | 2 | 2 | 4.6 | 4.6 | 4.6 | 56.166 |
| 2 | 2 | 2 | 2 | 2 | 3.6 | 3.6 | 3.6 | 63.817 |

| | | | | | | | | |
|---|---|---|---|---|---|---|---|---|
| 1 | 1 | 1 | 1 | 1 | 9.6 | 9.6 | 9.6 | 12.476 |
| 1 | 1 | 1 | 1 | 1 | 7.7 | 7.7 | 7.7 | 13.293 |
| 1 | 1 | 1 | 1 | 1 | 4 | 4 | 4 | 39.93 |
| 1 | 1 | 1 | 1 | 1 | 2.8 | 2.8 | 2.8 | 42.794 |
| 1 | 1 | 1 | 1 | 1 | 9.2 | 9.2 | 9.2 | 23.466 |
| 1 | 1 | 1 | 1 | 1 | 7.4 | 7.4 | 7.4 | 18.658 |
| 3 | 3 | 3 | 3 | 3 | 6.1 | 6.1 | 6.1 | 82.223 |
| 2 | 2 | 2 | 2 | 2 | 12.9 | 12.9 | 12.9 | 20.252 |
| 2 | 2 | 2 | 2 | 2 | 14.8 | 14.8 | 14.8 | 15.164 |
| 2 | 2 | 2 | 2 | 2 | 14.1 | 14.1 | 14.1 | 24.984 |
| 3 | 3 | 3 | 3 | 3 | 12.3 | 12.3 | 12.3 | 44.868 |
| 1;1 | 1;1 | 1 | 1 | 1 | 2.7 | 2.7 | 2.7 | 53.425 |
| 1 | 1 | 1 | 1 | 1 | 2.3 | 2.3 | 2.3 | 54.371 |
| 1 | 1 | 1 | 1 | 1 | 20 | 20 | 20 | 10.834 |
| 3 | 3 | 3 | 3 | 3 | 16.9 | 16.9 | 16.9 | 27.391 |
| 1 | 1 | 1 | 1 | 1 | 5.4 | 5.4 | 5.4 | 25.035 |
| 2 | 2 | 2 | 2 | 2 | 5.1 | 5.1 | 5.1 | 55.804 |
| 2 | 2 | 2 | 2 | 2 | 7.8 | 7.8 | 7.8 | 32.575 |
| 3 | 3 | 3 | 3 | 3 | 11.1 | 11.1 | 11.1 | 51.466 |
| 2 | 2 | 2 | 2 | 2 | 12.1 | 12.1 | 12.1 | 21.878 |
| 2 | 2 | 2 | 2 | 2 | 8.7 | 8.7 | 8.7 | 44.552 |
| 3 | 3 | 3 | 3 | 3 | 17.3 | 17.3 | 17.3 | 35.924 |
| 3 | 3 | 3 | 3 | 3 | 6.3 | 6.3 | 6.3 | 50.317 |
| 1 | 1 | 1 | 1 | 1 | 5 | 5 | 5 | 32.688 |
| 2 | 2 | 2 | 2 | 2 | 12.2 | 12.2 | 12.2 | 28.415 |
| 1 | 1 | 1 | 1 | 1 | 3.8 | 3.8 | 3.8 | 52.39 |
| 2 | 2 | 2 | 2 | 2 | 2.8 | 2.8 | 2.8 | 96.022 |
| 2 | 2 | 2 | 2 | 2 | 14 | 14 | 14 | 17.17 |
| 3 | 3 | 3 | 3 | 3 | 10.2 | 10.2 | 10.2 | 46.108 |
| 2 | 2 | 2 | 2 | 2 | 8 | 8 | 8 | 34.333 |
| 2 | 2 | 2 | 2 | 2 | 6.9 | 6.9 | 6.9 | 29.483 |
| 2 | 2 | 2 | 2 | 2 | 9.1 | 9.1 | 9.1 | 41.487 |
| 1 | 1 | 1 | 1 | 1 | 1.3 | 1.3 | 1.3 | 82.387 |
| 2;1;1;1 | 2;1;1;1 | 2 | 2 | 2 | 17.9 | 17.9 | 17.9 | 17.965 |
| 3 | 3 | 3 | 3 | 3 | 8 | 8 | 8 | 52.337 |
| 1 | 1 | 1 | 1 | 1 | 11.7 | 11.7 | 11.7 | 17.114 |
| 1 | 1 | 1 | 1 | 1 | 2.2 | 2.2 | 2.2 | 57.488 |
| 3 | 3 | 3 | 3 | 3 | 6.1 | 6.1 | 6.1 | 64.699 |

| | | | | | | | | | |
|---|---|---|---|---|---|---|---|---|---|
| | 1 | 1 | 1 | 1 | 1 | 3.2 | 3.2 | 3.2 | 45.745 |
| 2;0 | 2;0 | | 6 | 2 | 2 | 14.9 | 6.1 | 6.1 | 59.024 |
| | 2 | 2 | 2 | 2 | 2 | 6.1 | 6.1 | 6.1 | 43.973 |
| | 3 | 3 | 3 | 3 | 3 | 5.1 | 5.1 | 5.1 | 82.86 |
| 2;2 | 2;2 | | 2 | 2 | 2 | 17.1 | 17.1 | 17.1 | 8.496 |
| | 1 | 1 | 1 | 1 | 1 | 3.7 | 3.7 | 3.7 | 36.375 |
| | 2 | 2 | 2 | 2 | 2 | 14.5 | 14.5 | 14.5 | 18.648 |
| 1;1 | 1;1 | | 1 | 1 | 1 | 10.8 | 10.8 | 10.8 | 12.134 |
| 3;2 | 3;2 | | 3 | 3 | 3 | 10.7 | 10.7 | 10.7 | 50.582 |
| | 2 | 2 | 2 | 2 | 2 | 16.6 | 16.6 | 16.6 | 17.222 |
| | 2 | 2 | 2 | 2 | 2 | 7.3 | 7.3 | 7.3 | 37.572 |
| | 2 | 2 | 2 | 2 | 2 | 11.3 | 11.3 | 11.3 | 20.863 |
| 1;1;1 | 1;1;1 | | 1 | 1 | 1 | 5.7 | 5.7 | 5.7 | 24.033 |
| | 1 | 1 | 1 | 1 | 1 | 4.7 | 4.7 | 4.7 | 36.566 |
| | 1 | 1 | 1 | 1 | 1 | 2.7 | 2.7 | 2.7 | 49.541 |
| | 2 | 2 | 2 | 2 | 2 | 8.2 | 8.2 | 8.2 | 50.118 |
| | 2 | 2 | 2 | 2 | 2 | 16 | 16 | 16 | 24.701 |
| | 1 | 1 | 1 | 1 | 1 | 6.3 | 6.3 | 6.3 | 24.722 |
| | 1 | 1 | 1 | 1 | 1 | 9.7 | 9.7 | 9.7 | 20.478 |
| | 2 | 2 | 2 | 2 | 2 | 5.5 | 5.5 | 5.5 | 57.116 |
| | 3 | 3 | 3 | 3 | 3 | 6.3 | 6.3 | 6.3 | 73.114 |
| | 1 | 1 | 1 | 1 | 1 | 3.3 | 3.3 | 3.3 | 83.418 |
| | 2 | 2 | 2 | 2 | 2 | 6.8 | 6.8 | 6.8 | 49.432 |
| | 2 | 2 | 2 | 2 | 2 | 6.3 | 6.3 | 6.3 | 38.242 |
| | 1 | 1 | 1 | 1 | 1 | 5.7 | 5.7 | 5.7 | 29.555 |
| 2;2 | 2;2 | | 2 | 2 | 2 | 13.5 | 13.5 | 13.5 | 23.384 |
| | 1 | 1 | 1 | 1 | 1 | 23.5 | 23.5 | 23.5 | 7.5217 |
| | 1 | 1 | 1 | 1 | 1 | 7.9 | 7.9 | 7.9 | 22.619 |
| | 1 | 1 | 2 | 1 | 1 | 4.5 | 2.8 | 2.8 | 61.877 |
| | 1 | 1 | 1 | 1 | 1 | 6.3 | 6.3 | 6.3 | 20.108 |
| | 2 | 2 | 2 | 2 | 2 | 7.7 | 7.7 | 7.7 | 40.796 |
| | 3 | 3 | 3 | 3 | 3 | 13.1 | 13.1 | 13.1 | 40.542 |
| | 1 | 1 | 1 | 1 | 1 | 5.9 | 5.9 | 5.9 | 35.608 |
| | 1 | 1 | 1 | 1 | 1 | 2.3 | 2.3 | 2.3 | 61.448 |
| | 3 | 3 | 3 | 3 | 3 | 5.5 | 5.5 | 5.5 | 59.62 |
| | 1 | 1 | 1 | 1 | 1 | 2.5 | 2.5 | 2.5 | 59.425 |
| 3;1;1 | 3;1;1 | | 3 | 3 | 3 | 13.8 | 13.8 | 13.8 | 28.804 |
| 1;1;1 | 1;1;1 | | 7 | 1 | 1 | 23.6 | 3.4 | 3.4 | 49.83 |

| | | | | | | | | |
|---|---|---|---|---|---|---|---|---|
| 2 | 2 | 2 | 2 | 2 | 4.2 | 4.2 | 4.2 | 74.423 |
| 2 | 2 | 2 | 2 | 2 | 6.5 | 6.5 | 6.5 | 64.132 |
| 2 | 2 | 2 | 2 | 2 | 4.9 | 4.9 | 4.9 | 56.65 |
| 1 | 1 | 1 | 1 | 1 | 9.5 | 9.5 | 9.5 | 12.254 |
| 2 | 2 | 2 | 2 | 2 | 28.9 | 28.9 | 28.9 | 11.203 |
| 2 | 2 | 2 | 2 | 2 | 5.1 | 5.1 | 5.1 | 51.691 |
| 2 | 2 | 2 | 2 | 2 | 12.6 | 12.6 | 12.6 | 23.668 |
| 2 | 2 | 2 | 2 | 2 | 5.6 | 5.6 | 5.6 | 41.59 |
| 3 | 2 | 3 | 3 | 2 | 5.2 | 5.2 | 3.8 | 80.272 |
| 1 | 1 | 1 | 1 | 1 | 2.7 | 2.7 | 2.7 | 58.777 |
| 2 | 2 | 2 | 2 | 2 | 6.4 | 6.4 | 6.4 | 53.139 |
| 2 | 2 | 2 | 2 | 2 | 4.1 | 4.1 | 4.1 | 70.942 |
| 2 | 2 | 2 | 2 | 2 | 9.5 | 9.5 | 9.5 | 35.196 |
| 2;1 | 2;1 | 2 | 2 | 2 | 6.8 | 6.8 | 6.8 | 51.109 |
| 2 | 2 | 2 | 2 | 2 | 7.6 | 7.6 | 7.6 | 36.413 |
| 2 | 1 | 2 | 2 | 1 | 7.6 | 7.6 | 3.4 | 46.224 |
| 2 | 2 | 2 | 2 | 2 | 10.6 | 10.6 | 10.6 | 30.772 |
| 2;2 | 2;2 | 2 | 2 | 2 | 7.4 | 7.4 | 7.4 | 40.736 |
| 1 | 1 | 1 | 1 | 1 | 5.9 | 5.9 | 5.9 | 23.693 |
| 2 | 2 | 2 | 2 | 2 | 13 | 13 | 13 | 20.954 |
| 3 | 3 | 3 | 3 | 3 | 1.8 | 1.8 | 1.8 | 262.62 |
| 1 | 1 | 1 | 1 | 1 | 9.3 | 9.3 | 9.3 | 13.757 |
| 1 | 1 | 1 | 1 | 1 | 5.5 | 5.5 | 5.5 | 22.391 |
| 2 | 2 | 2 | 2 | 2 | 3.5 | 3.5 | 3.5 | 99.986 |
| 2 | 2 | 2 | 2 | 2 | 5.2 | 5.2 | 5.2 | 64.135 |
| 1;1;1 | 1;1;1 | 1 | 1 | 1 | 13.7 | 13.7 | 13.7 | 12.582 |
| 2 | 2 | 2 | 2 | 2 | 13.7 | 13.7 | 13.7 | 25.898 |
| 1 | 1 | 1 | 1 | 1 | 6 | 6 | 6 | 20.126 |
| 1 | 1 | 1 | 1 | 1 | 4.2 | 4.2 | 4.2 | 25.357 |
| 1 | 1 | 1 | 1 | 1 | 3.2 | 3.2 | 3.2 | 34.746 |
| 1;1;1 | 1;1;1 | 1 | 1 | 1 | 0.9 | 0.9 | 0.9 | 152.58 |
| 1;1;1;1 | 1;1;1;1 | 1 | 1 | 1 | 1.5 | 1.5 | 1.5 | 111.75 |
| 2 | 2 | 2 | 2 | 2 | 3.9 | 3.9 | 3.9 | 80.852 |
| 2 | 2 | 2 | 2 | 2 | 4.7 | 4.7 | 4.7 | 58.024 |
| 1 | 1 | 1 | 1 | 1 | 2.7 | 2.7 | 2.7 | 73.776 |
| 3;2 | 3;2 | 3 | 3 | 3 | 3.2 | 3.2 | 3.2 | 115.93 |
| 1 | 1 | 1 | 1 | 1 | 5.2 | 5.2 | 5.2 | 26.396 |
| 1 | 1 | 1 | 1 | 1 | 7.7 | 7.7 | 7.7 | 22.127 |

| | | | | | | | | |
|---|---|---|---|---|---|---|---|---|
| 2 | 2 | 2 | 2 | 2 | 17.1 | 17.1 | 17.1 | 8.2178 |
| 2 | 2 | 2 | 2 | 2 | 6.7 | 6.7 | 6.7 | 60.13 |
| 2;1;1 | 2;1;1 | 2 | 2 | 2 | 11.6 | 11.6 | 11.6 | 23.707 |
| 1 | 1 | 1 | 1 | 1 | 9.6 | 9.6 | 9.6 | 15.86 |
| 1 | 1 | 1 | 1 | 1 | 3.7 | 3.7 | 3.7 | 57.839 |
| 2 | 2 | 3 | 2 | 2 | 17.5 | 13.4 | 13.4 | 28.082 |
| 2 | 2 | 2 | 2 | 2 | 9.4 | 9.4 | 9.4 | 39.724 |
| 1 | 1 | 1 | 1 | 1 | 4 | 4 | 4 | 34.814 |
| 2 | 2 | 2 | 2 | 2 | 5.1 | 5.1 | 5.1 | 53.801 |
| 1 | 1 | 1 | 1 | 1 | 7.6 | 7.6 | 7.6 | 17 |
| 2 | 2 | 2 | 2 | 2 | 12.5 | 12.5 | 12.5 | 27.076 |
| 2 | 2 | 2 | 2 | 2 | 5.8 | 5.8 | 5.8 | 37.512 |
| 1 | 1 | 1 | 1 | 1 | 5.4 | 5.4 | 5.4 | 21.539 |
| 2 | 2 | 2 | 2 | 2 | 10.3 | 10.3 | 10.3 | 27.679 |
| 1 | 1 | 1 | 1 | 1 | 10.5 | 10.5 | 10.5 | 11.74 |
| 1;1 | 1;1 | 6 | 1 | 1 | 20.9 | 3.4 | 3.4 | 49.953 |
| 1 | 1 | 1 | 1 | 1 | 3.2 | 3.2 | 3.2 | 41.949 |
| 1 | 1 | 1 | 1 | 1 | 24.7 | 24.7 | 24.7 | 10.35 |
| 3 | 3 | 3 | 3 | 3 | 20.5 | 20.5 | 20.5 | 23.356 |
| 1 | 1 | 1 | 1 | 1 | 6.4 | 6.4 | 6.4 | 31.462 |
| 1 | 1 | 1 | 1 | 1 | 10.6 | 10.6 | 10.6 | 12.259 |
| 2 | 2 | 2 | 2 | 2 | 9.1 | 9.1 | 9.1 | 35.076 |
| 2 | 2 | 2 | 2 | 2 | 5.6 | 5.6 | 5.6 | 54.416 |
| 1 | 1 | 1 | 1 | 1 | 1.7 | 1.7 | 1.7 | 64.066 |
| 1 | 1 | 1 | 1 | 1 | 7.7 | 7.7 | 7.7 | 19.529 |
| 1 | 1 | 2 | 1 | 1 | 18.3 | 8.3 | 8.3 | 20.511 |
| 2 | 2 | 2 | 2 | 2 | 3.9 | 3.9 | 3.9 | 68.996 |
| 1 | 1 | 1 | 1 | 1 | 4.6 | 4.6 | 4.6 | 31.121 |
| 1 | 1 | 1 | 1 | 1 | 4.2 | 4.2 | 4.2 | 39.393 |
| 1 | 1 | 1 | 1 | 1 | 4.3 | 4.3 | 4.3 | 25.535 |
| 1 | 1 | 1 | 1 | 1 | 2.6 | 2.6 | 2.6 | 55.992 |
| 1 | 1 | 1 | 1 | 1 | 4.7 | 4.7 | 4.7 | 28.433 |
| 1 | 1 | 1 | 1 | 1 | 8.1 | 8.1 | 8.1 | 16.537 |
| 1 | 1 | 1 | 1 | 1 | 7 | 7 | 7 | 23.897 |
| 1 | 1 | 1 | 1 | 1 | 6.7 | 6.7 | 6.7 | 22.427 |
| 1 | 1 | 1 | 1 | 1 | 5.2 | 5.2 | 5.2 | 37.567 |
| 1 | 1 | 1 | 1 | 1 | 8.6 | 8.6 | 8.6 | 13.459 |
| 1 | 1 | 1 | 1 | 1 | 6.4 | 6.4 | 6.4 | 31.982 |

| | | | | | | | | |
|---|---|---|---|---|---|---|---|---|
| 2;2 | 2;2 | 2 | 2 | 2 | 5.9 | 5.9 | 5.9 | 42.141 |
| 1 | 1 | 1 | 1 | 1 | 5.8 | 5.8 | 5.8 | 25.673 |
| 3 | 3 | 3 | 3 | 3 | 6.6 | 6.6 | 6.6 | 68.303 |
| 1 | 1 | 1 | 1 | 1 | 4.5 | 4.5 | 4.5 | 42.981 |
| 1 | 1 | 1 | 1 | 1 | 5 | 5 | 5 | 37.67 |
| 1 | 1 | 1 | 1 | 1 | 4.2 | 4.2 | 4.2 | 26.659 |
| 1 | 1 | 1 | 1 | 1 | 3 | 3 | 3 | 55.08 |
| 1 | 1 | 1 | 1 | 1 | 10 | 10 | 10 | 11.284 |
| 1;1 | 1;1 | 1 | 1 | 1 | 4.8 | 4.8 | 4.8 | 34.596 |
| 1 | 1 | 1 | 1 | 1 | 4.3 | 4.3 | 4.3 | 34.36 |
| 1 | 1 | 1 | 1 | 1 | 2.2 | 2.2 | 2.2 | 95.785 |
| 1 | 1 | 1 | 1 | 1 | 4.9 | 4.9 | 4.9 | 28.48 |
| 2;1 | 2;1 | 2 | 2 | 2 | 4.9 | 4.9 | 4.9 | 57.221 |
| 2 | 2 | 2 | 2 | 2 | 20 | 20 | 20 | 14.716 |
| 2 | 2 | 2 | 2 | 2 | 5.9 | 5.9 | 5.9 | 59.38 |
| 1;1 | 1;1 | 1 | 1 | 1 | 4.2 | 4.2 | 4.2 | 30.512 |
| 1 | 1 | 1 | 1 | 1 | 3.1 | 3.1 | 3.1 | 66.193 |
| 1 | 1 | 1 | 1 | 1 | 7 | 7 | 7 | 26.888 |
| 2 | 2 | 2 | 2 | 2 | 8.7 | 8.7 | 8.7 | 33.697 |
| 1 | 1 | 1 | 1 | 1 | 3.1 | 3.1 | 3.1 | 60.54 |
| 1;1;1;1;1;1 | 1;1;1;1;1 | 1 | 1 | 1 | 2.7 | 2.7 | 2.7 | 42.766 |
| 1 | 1 | 1 | 1 | 1 | 3.9 | 3.9 | 3.9 | 43.383 |
| 1 | 1 | 1 | 1 | 1 | 3.1 | 3.1 | 3.1 | 47.573 |
| 2 | 2 | 2 | 2 | 2 | 4.5 | 4.5 | 4.5 | 57.21 |
| 2 | 2 | 2 | 2 | 2 | 7.3 | 7.3 | 7.3 | 37.404 |
| 1 | 1 | 1 | 1 | 1 | 1.7 | 1.7 | 1.7 | 62.639 |
| 1;1 | 1;1 | 1 | 1 | 1 | 1.1 | 1.1 | 1.1 | 87.793 |
| 1 | 1 | 1 | 1 | 1 | 3.2 | 3.2 | 3.2 | 42.486 |
| 1 | 1 | 1 | 1 | 1 | 4.5 | 4.5 | 4.5 | 51.522 |
| 1 | 1 | 1 | 1 | 1 | 7.5 | 7.5 | 7.5 | 16.84 |
| 1;1 | 1;1 | 1 | 1 | 1 | 7.9 | 7.9 | 7.9 | 21.656 |
| 1 | 1 | 1 | 1 | 1 | 2.9 | 2.9 | 2.9 | 76.631 |
| 1 | 1 | 1 | 1 | 1 | 4.8 | 4.8 | 4.8 | 38.746 |
| 1 | 1 | 1 | 1 | 1 | 3.7 | 3.7 | 3.7 | 50.559 |
| 1 | 1 | 1 | 1 | 1 | 14.7 | 14.7 | 14.7 | 10.5 |
| 1 | 1 | 1 | 1 | 1 | 0.8 | 0.8 | 0.8 | 173.1 |
| 1 | 1 | 1 | 1 | 1 | 8.1 | 8.1 | 8.1 | 18.697 |
| 1 | 1 | 1 | 1 | 1 | 4.4 | 4.4 | 4.4 | 31.284 |

| | | | | | | | | |
|---|---|---|---|---|---|---|---|---|
| 1 | 1 | 1 | 1 | 1 | 6.4 | 6.4 | 6.4 | 22.876 |
| 1 | 1 | 1 | 1 | 1 | 12.3 | 12.3 | 12.3 | 18.243 |
| 1 | 1 | 1 | 1 | 1 | 1.4 | 1.4 | 1.4 | 80.472 |
| 1 | 1 | 1 | 1 | 1 | 3.6 | 3.6 | 3.6 | 53.165 |
| 1 | 1 | 1 | 1 | 1 | 8.5 | 8.5 | 8.5 | 31.263 |
| 1 | 1 | 1 | 1 | 1 | 15.9 | 15.9 | 15.9 | 7.8409 |
| 1 | 1 | 1 | 1 | 1 | 5 | 5 | 5 | 29.717 |
| 1 | 1 | 1 | 1 | 1 | 4.9 | 4.9 | 4.9 | 34.208 |
| 1 | 1 | 1 | 1 | 1 | 2.7 | 2.7 | 2.7 | 46.836 |
| 1 | 1 | 1 | 1 | 1 | 2.5 | 2.5 | 2.5 | 50.18 |
| 1 | 1 | 1 | 1 | 1 | 3.1 | 3.1 | 3.1 | 36.983 |
| 1 | 1 | 1 | 1 | 1 | 4.5 | 4.5 | 4.5 | 44.468 |
| 1 | 1 | 1 | 1 | 1 | 10.3 | 10.3 | 10.3 | 14.57 |
| 1 | 1 | 1 | 1 | 1 | 2.6 | 2.6 | 2.6 | 48.84 |
| 1 | 1 | 1 | 1 | 1 | 2.1 | 2.1 | 2.1 | 72.453 |
| 1;1;1 | 1;1;1 | 1 | 1 | 1 | 8 | 8 | 8 | 22.171 |
| 1 | 1 | 1 | 1 | 1 | 12.5 | 12.5 | 12.5 | 12.473 |
| 1 | 1 | 1 | 1 | 1 | 2.5 | 2.5 | 2.5 | 53.524 |
| 1 | 1 | 1 | 1 | 1 | 14 | 14 | 14 | 11.514 |
| 1 | 1 | 1 | 1 | 1 | 6.1 | 6.1 | 6.1 | 22.367 |
| 1 | 1 | 1 | 1 | 1 | 7.4 | 7.4 | 7.4 | 27.325 |
| 1 | 1 | 1 | 1 | 1 | 6.1 | 6.1 | 6.1 | 24.604 |
| 1 | 1 | 1 | 1 | 1 | 5.1 | 5.1 | 5.1 | 25.097 |
| 1 | 1 | 1 | 1 | 1 | 2.6 | 2.6 | 2.6 | 62.942 |
| 1 | 1 | 1 | 1 | 1 | 11.8 | 11.8 | 11.8 | 10.834 |
| 1 | 1 | 1 | 1 | 1 | 1.2 | 1.2 | 1.2 | 138.94 |
| 1 | 1 | 1 | 1 | 1 | 7.5 | 7.5 | 7.5 | 22.836 |
| 1;1 | 1;1 | 1 | 1 | 1 | 6.2 | 6.2 | 6.2 | 24.614 |
| 1 | 1 | 1 | 1 | 1 | 0.6 | 0.6 | 0.6 | 178.19 |
| 1 | 1 | 1 | 1 | 1 | 3.7 | 3.7 | 3.7 | 27.596 |
| 1 | 1 | 1 | 1 | 1 | 2.2 | 2.2 | 2.2 | 68.259 |
| 1 | 1 | 1 | 1 | 1 | 1.6 | 1.6 | 1.6 | 112.59 |
| 1 | 1 | 1 | 1 | 1 | 4.5 | 4.5 | 4.5 | 37.489 |
| 1 | 1 | 1 | 1 | 1 | 4 | 4 | 4 | 48.455 |
| 1 | 1 | 2 | 1 | 1 | 6.2 | 3.1 | 3.1 | 40.45 |
| 1 | 1 | 1 | 1 | 1 | 2.3 | 2.3 | 2.3 | 52.049 |
| 1;1 | 1;1 | 1 | 1 | 1 | 1.9 | 1.9 | 1.9 | 100.07 |
| 1 | 1 | 1 | 1 | 1 | 0.5 | 0.5 | 0.5 | 269.76 |

| | | | | | | | | | |
|---|---|---|---|---|---|---|---|---|---|
| 2 | 2 | 2 | 2 | 2 | 12.6 | 12.6 | 12.6 | 20.7 | |
| 1 | 1 | 1 | 1 | 1 | 2.3 | 2.3 | 2.3 | 57.206 | |
| 2 | 2 | 2 | 2 | 2 | 8.7 | 8.7 | 8.7 | 39.547 | |
| 1 | 1 | 1 | 1 | 1 | 6.9 | 6.9 | 6.9 | 24.146 | |
| 1 | 1 | 1 | 1 | 1 | 1.5 | 1.5 | 1.5 | 81.529 | |
| 1 | 1 | 1 | 1 | 1 | 2.8 | 2.8 | 2.8 | 60.246 | |
| 1 | 1 | 6 | 1 | 1 | 24.6 | 4.2 | 4.2 | 35.575 | |
| 1 | 1 | 1 | 1 | 1 | 15.8 | 15.8 | 15.8 | 15.892 | |
| 1 | 1 | 1 | 1 | 1 | 1.6 | 1.6 | 1.6 | 78.365 | |
| 1 | 1 | 1 | 1 | 1 | 2.6 | 2.6 | 2.6 | 65.443 | |
| 1;1 | 1;1 | 1 | 1 | 1 | 3.6 | 3.6 | 3.6 | 39.503 | |
| 1 | 1 | 1 | 1 | 1 | 3.9 | 3.9 | 3.9 | 55.342 | |
| 1 | 1 | 1 | 1 | 1 | 7.1 | 7.1 | 7.1 | 20.811 | |
| 1 | 1 | 1 | 1 | 1 | 2.9 | 2.9 | 2.9 | 52.562 | |
| 1 | 1 | 1 | 1 | 1 | 4.3 | 4.3 | 4.3 | 29.506 | |
| 1 | 1 | 1 | 1 | 1 | 1.3 | 1.3 | 1.3 | 129.63 | |
| 1 | 1 | 1 | 1 | 1 | 2.9 | 2.9 | 2.9 | 59.366 | |
| 2 | 2 | 2 | 2 | 2 | 21 | 21 | 21 | 16.628 | |
| 1 | 1 | 1 | 1 | 1 | 4.6 | 4.6 | 4.6 | 42.777 | |
| 1 | 1 | 1 | 1 | 1 | 1.6 | 1.6 | 1.6 | 72.683 | |
| 1 | 1 | 1 | 1 | 1 | 6.9 | 6.9 | 6.9 | 21.007 | |
| 1 | 1 | 1 | 1 | 1 | 4.4 | 4.4 | 4.4 | 24.347 | |
| 1 | 1 | 1 | 1 | 1 | 5.1 | 5.1 | 5.1 | 38.438 | |
| 1 | 1 | 1 | 1 | 1 | 2.9 | 2.9 | 2.9 | 43.072 | |
| 1;1;1 | 1;1;1 | 1 | 1 | 1 | 5.3 | 5.3 | 5.3 | 34.834 | |
| 1 | 1 | 1 | 1 | 1 | 13.8 | 13.8 | 13.8 | 11.748 | |
| 1;1 | 1;1 | 1 | 1 | 1 | 3.3 | 3.3 | 3.3 | 33.6 | |
| 1 | 1 | 1 | 1 | 1 | 4.2 | 4.2 | 4.2 | 34.063 | |
| 1 | 1 | 1 | 1 | 1 | 1.3 | 1.3 | 1.3 | 95.737 | |
| 1 | 1 | 1 | 1 | 1 | 11.8 | 11.8 | 11.8 | 12.405 | |
| 1 | 1 | 1 | 1 | 1 | 1.8 | 1.8 | 1.8 | 63.944 | |
| 1;1 | 1;1 | 1 | 1 | 1 | 2.5 | 2.5 | 2.5 | 46.153 | |
| 1 | 1 | 1 | 1 | 1 | 1.4 | 1.4 | 1.4 | 114.53 | |
| 1 | 1 | 1 | 1 | 1 | 4.5 | 4.5 | 4.5 | 55.556 | |
| 1 | 1 | 1 | 1 | 1 | 4.7 | 4.7 | 4.7 | 35.54 | |
| 2 | 2 | 2 | 2 | 2 | 3.3 | 3.3 | 3.3 | 72.911 | |
| 1 | 1 | 1 | 1 | 1 | 8.8 | 8.8 | 8.8 | 21.445 | |
| 1 | 1 | 1 | 1 | 1 | 1.3 | 1.3 | 1.3 | 73.363 | |

| | | | | | | | | |
|---|---|---|---|---|---|---|---|---|
| 1 | 1 | 1 | 1 | 1 | 4.1 | 4.1 | 4.1 | 60.343 |
| 1;1 | 1;1 | 1 | 1 | 1 | 1.9 | 1.9 | 1.9 | 94.469 |
| 1 | 1 | 1 | 1 | 1 | 4.1 | 4.1 | 4.1 | 51.958 |
| 1 | 1 | 1 | 1 | 1 | 2.8 | 2.8 | 2.8 | 70.414 |
| 1 | 1 | 1 | 1 | 1 | 2 | 2 | 2 | 84.659 |
| 1 | 1 | 1 | 1 | 1 | 1.8 | 1.8 | 1.8 | 66.851 |
| 1 | 1 | 1 | 1 | 1 | 1.8 | 1.8 | 1.8 | 52.739 |
| 1;1 | 1;1 | 1 | 1 | 1 | 2.2 | 2.2 | 2.2 | 61.433 |
| 1 | 1 | 1 | 1 | 1 | 6.5 | 6.5 | 6.5 | 28.262 |
| 1 | 1 | 1 | 1 | 1 | 1.6 | 1.6 | 1.6 | 84.488 |
| 1 | 1 | 1 | 1 | 1 | 5.9 | 5.9 | 5.9 | 27.401 |
| 1 | 1 | 1 | 1 | 1 | 7.8 | 7.8 | 7.8 | 17.561 |
| 1 | 1 | 1 | 1 | 1 | 1.9 | 1.9 | 1.9 | 81.289 |
| 1 | 1 | 1 | 1 | 1 | 4.5 | 4.5 | 4.5 | 24.682 |
| 1 | 1 | 1 | 1 | 1 | 3.8 | 3.8 | 3.8 | 63.972 |
| 1 | 1 | 1 | 1 | 1 | 7.4 | 7.4 | 7.4 | 17.79 |
| 1 | 1 | 1 | 1 | 1 | 4.5 | 4.5 | 4.5 | 39.867 |
| 1 | 1 | 1 | 1 | 1 | 1 | 1 | 1 | 122.2 |
| 1 | 1 | 1 | 1 | 1 | 4 | 4 | 4 | 28.38 |
| 2 | 2 | 2 | 2 | 2 | 7.5 | 7.5 | 7.5 | 47.079 |
| 1 | 1 | 1 | 1 | 1 | 2 | 2 | 2 | 57.924 |
| 1 | 1 | 1 | 1 | 1 | 1.9 | 1.9 | 1.9 | 51.294 |
| 1 | 1 | 3 | 1 | 1 | 9.8 | 2.4 | 2.4 | 49.263 |
| 1 | 1 | 2 | 1 | 1 | 6.5 | 2.4 | 2.4 | 47.377 |
| 1 | 1 | 1 | 1 | 1 | 2.7 | 2.7 | 2.7 | 53.542 |
| 1 | 1 | 1 | 1 | 1 | 3.3 | 3.3 | 3.3 | 71.456 |
| 1 | 1 | 1 | 1 | 1 | 1.8 | 1.8 | 1.8 | 99.968 |
| 1 | 1 | 1 | 1 | 1 | 0.7 | 0.7 | 0.7 | 128.98 |
| 1 | 1 | 1 | 1 | 1 | 5.7 | 5.7 | 5.7 | 21.732 |
| 1 | 1 | 1 | 1 | 1 | 1.7 | 1.7 | 1.7 | 65.401 |
| 1 | 1 | 1 | 1 | 1 | 1.3 | 1.3 | 1.3 | 97.207 |
| 1 | 1 | 1 | 1 | 1 | 3.4 | 3.4 | 3.4 | 43.747 |
