# Supplementary material for: Overcoming the blood–brain barrier by Annexin A1-binding peptide to target brain tumours
Source: Br J Cancer. 2020 Sep 14;123(11):1633–43. doi: 10.1038/s41416-020-01066-2 (PMC7686308; doi:10.1038/s41416-020-01066-2)
Supplement: Supplementary file 3 — Supplemental Table 2 [file 41416_2020_1066_MOESM3_ESM.pdf]

Sequence	Proteins	Leading razor protein	Start position	End position	Gene names	Protein names	Unique (Groups)	Unique (Proteins)	Intensity	Potential contaminant	MS/MS Count
AAYLQETGKPLD	P04083	P04083	82	93	ANXA1	Annexin A1	yes	yes	6551300		1
AAYLQETGKPLDET	P04083	P04083	82	95	ANXA1	Annexin A1	yes	yes	0		1
AAYLQETGKPLDETL	P04083	P04083	82	96	ANXA1	Annexin A1	yes	yes	792410		1
AAYLQETGKPLDETLK	P04083	P04083	82	97	ANXA1	Annexin A1	yes	yes	174180000		20
AAYLQETGKPLDETLKK	P04083	P04083	82	98	ANXA1	Annexin A1	yes	yes	297920000		22
ALYEAGER	P04083	P04083	205	212	ANXA1	Annexin A1	yes	yes	0		1
ALYEAGERR	P04083	P04083	205	213	ANXA1	Annexin A1	yes	yes	122150000		1
ATIIDILTK	P04083	P04083	63	71	ANXA1	Annexin A1	yes	yes	337890		1
CQAILDETK	P04083	P04083	324	332	ANXA1	Annexin A1	yes	yes	10565000		2
CQAILDETKGDYEK	P04083	P04083	324	337	ANXA1	Annexin A1	yes	yes	739610		1
DEATIIDILTKR	P04083	P04083	61	72	ANXA1	Annexin A1	yes	yes	169690000		1
DITSDTSGDFR	P04083	P04083	167	177	ANXA1	Annexin A1	yes	yes	3740300000		11
DITSDTSGDFRN	P04083	P04083	167	178	ANXA1	Annexin A1	yes	yes	25980000		2
DLAKDITSDTSGDFR	P04083	P04083	163	177	ANXA1	Annexin A1	yes	yes	23944000		1
GTDVNVFNTILTTR	P04083	P04083	215	228	ANXA1	Annexin A1	yes	yes	7747900000		48
GVDEATIIDILTK	P04083	P04083	59	71	ANXA1	Annexin A1	yes	yes	7401600000		32
GVDEATIIDILTKR	P04083	P04083	59	72	ANXA1	Annexin A1	yes	yes	172230000		3
ILVALCGGN	P04083	P04083	338	346	ANXA1	Annexin A1	yes	yes	424290000		13
KAAYLQETGKPLDETLK	P04083	P04083	81	97	ANXA1	Annexin A1	yes	yes	10101000		1
KGTDVNVFNTILTTR	P04083	P04083	214	228	ANXA1	Annexin A1	yes	yes	18590000		8
KTPAQFDADELR	P04083	P04083	113	124	ANXA1	Annexin A1	yes	yes	8535200		2
PAQFDADELR	P04083	P04083	115	124	ANXA1	Annexin A1	yes	yes	0		1
PSSDVAALHK	P04083	P04083	44	53	ANXA1	Annexin A1	yes	yes	7187100		1
TDVNVFNTILTTR	P04083	P04083	216	228	ANXA1	Annexin A1	yes	yes	2222400		1
TPAQFDADELR	P04083	P04083	114	124	ANXA1	Annexin A1	yes	yes	4166500000		24
TPAQFDADELRAA	P04083	P04083	114	126	ANXA1	Annexin A1	yes	yes	509510		1
TPAQFDADELRAAMK	P04083	P04083	114	128	ANXA1	Annexin A1	yes	yes	18143000		2
TSDTSGDFR	P04083	P04083	169	177	ANXA1	Annexin A1	yes	yes	7606600		1
VFNTILTTR	P04083	P04083	220	228	ANXA1	Annexin A1	yes	yes	2264200		1
YGISLCQAILDETK	P04083	P04083	319	332	ANXA1	Annexin A1	yes	yes	2110500		1
